# PISAD: reference-free intraspecies sample anomalies detection tool based on *k*-mer counting

**DOI:** 10.1093/gigascience/giaf061

**Published:** 2025-06-17

**Authors:** Zhantian Xu, Fan Nie, Jianxin Wang

**Affiliations:** School of Computer Science and Engineering, Central South University, Changsha 410083, China; Xiangjiang Laboratory, Changsha 410205, China; Hunan Provincial Key Lab on Bioinformatics, Central South University, Changsha 410083, China; National Center for Applied Mathematics in Hunan and Key Laboratory of Intelligent Computing and Information Processing of Ministry of Education, Xiangtan University, Hunan 411105, China; School of Computer Science and Engineering, Central South University, Changsha 410083, China; Xiangjiang Laboratory, Changsha 410205, China; Hunan Provincial Key Lab on Bioinformatics, Central South University, Changsha 410083, China; Xinjiang Engineering Research Center of Big Data and Intelligent Software, School of Software, Xinjiang University, Urumqi 830091, China

**Keywords:** sample swap, SNP calling, reference-free, *k*-mer analysis, quality control

## Abstract

**Background:**

Genomic sequencing research often requires the simultaneous analysis of heterogeneous data types across single or multiple individuals, introducing a substantial risk of sample swaps (e.g., labeling errors). Existing methods primarily rely on reference information, requiring the preselection of informative variant sites with a population allele frequency around 0.5, which may be insufficient or unavailable for nonmodel organisms. As research expands to encompass a growing number of new species, a robust quality control tool will become increasingly important.

**Finds:**

We developed PISAD (Phased Intraspecies Sample Anomalies Detection), a tool for validating sample identities in whole-genome sequencing (WGS) data without requiring reference information. It uses a 2-stage approach: first, it performs rapid, reference-free single nucleotide polymorphism (SNP) calling on low-error-rate data from the target individual to create a variant sketch; then, it assesses the concordance of other samples on this sketch to verify relationships. We assessed the performance and efficiency of PISAD on *Homo sapiens, Bos taurus, Gallus gallus, Arctia plantaginis*, and *Pyrus* species.

**Conclusions:**

Our evaluation showed that PISAD achieves a lower data coverage requirement (0.5×) compared to the reference-based tool ntsm and is broadly applicable to multiple diploid species.

## Introduction

Whole-genome sequencing (WGS) studies often involve multiple or single individuals across various experiments using different sequencing technologies (e.g., Illumina, PacBio, Oxford Nanopore Technologies, Hi-C, etc.). For instance, *de novo* assembly often involves sequencing data from multiple technologies [1–3] to improve assembly quality. Moreover, sequencing data from each technology may involve multiple sequencing runs. Each new procedure or handling introduces potential opportunities for sample swap. Even a single sample swap can have severe consequences on downstream analyses. Therefore, confirming the relatedness of samples assumed to come from the same donor is an essential step in quality control (QC), which should be performed as early as possible in the analysis pipeline.

Existing sample swap detection methods can be divided into 2 categories based on the source of the sample: cross-species and same-species. Cross-species swaps have been extensively studied. For example, Mash (RRID:SCR_019135) [4] uses MinHash techniques to rapidly calculate the genomic distance to identify them. However, in same-species swaps, the high genetic similarity among samples can obscure the differences. Current approaches for detecting same-species swaps primarily rely on genotypes at single nucleotide polymorphisms (SNPs), leveraging predetermined variant sites constructed from population-level allele frequency to distinguish between samples [5–12]. For instance, Peddy (RRID:SCR_017287) [6] extracts genotypes at preselected variant sites from VCF files for each sample and uses the kinship calculation method from KING (RRID:SCR_009251) [13] to determine the relationships between samples. At the same time, Somalier [10] accelerates relationship calculation between samples by creating sketches for rapid comparison. NGSCheckMate (RRID:SCR_022994) [9] verifies sample identity in next-generation sequencing (NGS) data by calculating the variant allele fractions at preselected SNP sites using a model-based approach. CrossCheck [8] leverages linkage disequilibrium to achieve improved accuracy in shallow sequencing. ntsm (RRID:SCR_024994) [11] leverages *k*-mer counting and maximum likelihood estimation, making it suitable for low-coverage and heterogeneous whole-genome sequencing data.

While current approaches for detecting sample swaps have been successful across a wide range of applications, most are limited to human samples. Although ntsm introduces a method for extracting informative variant sites and optimistically suggests applicability to other species, its performance may degrade or even fail in species where research is still in its early stages and population-level allele frequency information is limited or unavailable.

To address this problem, we use a reference-free SNP calling approach to construct variation sketches, eliminating the need for predefined variant sites. Currently, 2 main methods are available for reference-free SNP calling. In hybrid approaches, raw reads are assembled into long contigs or scaffolds, and SNPs are then identified by aligning the raw reads to these assembled contigs and mapping them to specific positions [14–16]. The accuracy of this method depends heavily on assembly quality, and the assembly step itself is time-consuming [17]. The second approach processes data directly based on *k*-mer counting. For example, Cortex [18] and DiscoSnp++ (RRID:SCR_002612) [17, 19] construct a de Bruijn graph from raw data and detect specific patterns to call SNPs. ebwt2snp [20,21] uses the extended Burrows–Wheeler transform (eBWT) from reads to identify SNPs as pairs of *k*-mers. Kmer2SNP [22] simplifies the heterozygous SNP calling problem by finding the maximum weight matching in the heterozygous *k*-mer graph. Unlike previous methods, it selects *k*-mers from heterozygous regions based on *k*-mer frequency distribution rather than using all *k*-mers, substantially reducing data volume and largely mitigating the impact of homologous repetitive sequences. However, the existing Kmer2SNP approach is not suitable for low-coverage data, and the speed and precision of SNP calling remain bottlenecks for downstream analysis, prompting us to introduce improvements.

In this work, we developed PISAD, a **P**hased **I**ntraspecies **S**ample **A**nomalies **D**etection tool, suitable for multiple species without reference information. The tool eliminates the need for reference information by using a reference-free SNP calling approach to construct variant sketches. Additionally, we improve the SNP calling method that is an order of magnitude faster and enable the detection of sample swaps using only heterozygous SNP information by refining the calculation of intersample relationships. Our process requires no additional reference information or downstream steps, such as alignment, making it an efficient QC tool for the upstream stage.

## Methods

### Algorithm overview

**Figure 1:**
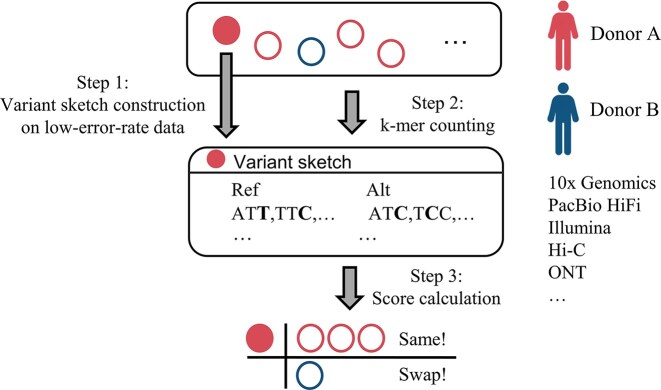
A schematic overview of PISAD. Red and blue circles represent sequencing runs from different donors with heterogeneous data types. The solid circle indicates the data used for variant sketch construction, which, in practice, can be any low-error-rate data (e.g., 10x Genomics, PacBio HiFi, etc.). The mix of blue and red circles represents sample swaps, indicating that samples are incorrectly assigned to the wrong donor.

We developed PISAD, a tool designed to detect anomalies in cohort samples without requiring reference information. The tool operates in 2 primary stages. In stage 1, we performed reference-free SNP calling to construct a variant sketch using low-error-rate data from the target individual. In stage 2, we compared the *k*-mer counts of other cohort samples on the variant sketch to infer relationships between them (Fig. 1). It is important to note that stage 1 of our tool supports only low-error-rate sequencing data, meaning most ONT data (except duplex) cannot be processed. However, stage 2 is compatible with various sequencing technologies. Therefore, at least 1 low-error-rate dataset is required for reliable sketch construction.

### SNP calling

In SNP calling, the first step is to select heterozygous *k*-mers to construct the vertex set. Kmer2SNP uses DSK (RRID:SCR_001246) [23] to count *k*-mer frequencies from raw reads and generate a corresponding *k*-mer histogram file. Then, FindGse [24] is employed to identify the frequency range of heterozygous *k*-mers. Existing genome analysis tools, such as FindGse, are typically designed for high-coverage data ($>30\times$) and are not well suited for shallow sequencing. Therefore, we developed a heuristic algorithm to estimate the range of heterozygous regions under low-coverage conditions.

The algorithm begins by reading the first 1,000 entries from the histogram of *k*-mer abundances, which records the number of distinct *k*-mers for each occurrence frequency and then checks for a sequence of 3 consecutive points that shows an upward trend followed by a downward trend. If such a sequence is found, it is defined as a peak. To filter out noise from small peaks, the algorithm will terminate early if either 95% of the total *k*-mer frequency has been read or if 2 peaks have already been identified. When 2 peaks are found, the first peak is assumed to represent the heterozygous peak. If only 1 peak is identified due to low coverage, an additional parameter is required to indicate whether the heterozygosity rate of the species is greater than 1.2%. This distinction is necessary because, with only 1 peak, the algorithm cannot reliably determine whether it represents a homozygous or heterozygous region. According to GenomeScope (RRID:SCR_017014) [25], when the heterozygosity rate exceeds about 1.2%, the frequency of the heterozygous peak begins to surpass that of the homozygous peak. Once the heterozygous peak value is identified, the heterozygous region is calculated.

**Figure 2:**
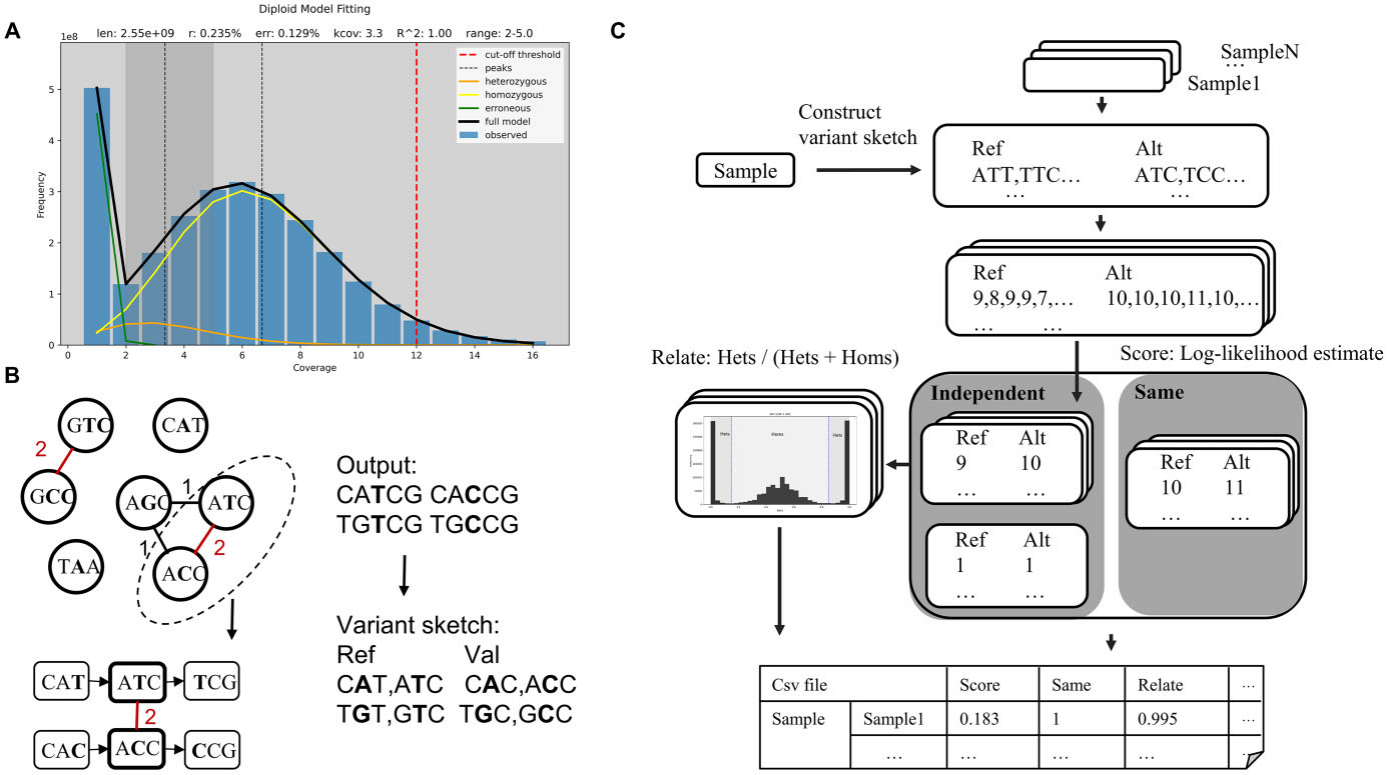
Illustration of key steps. (A) A schematic diagram illustrating the algorithm’s selection of heterozygous regions, using HG002 data at 6× coverage as an example. The dark gray areas represent the designated heterozygous regions, while the top of the figure displays genomic characteristics estimated using 2 negative binomial distributions. (B) Stage 1: Calling isolated SNPs on low-error-rate data to construct a variant sketch. The left-side diagram illustrates *k*-mers located in heterozygous regions (represented as circles). The connecting lines between the circles indicate *k*-mer pairings (with a single nucleotide difference in the middle), and the numbers on these lines represent the support length (i.e., the maximum possible expansion distance). The red-colored SNPs indicate the isolated SNPs ultimately selected through maximum-weight matching. (C) Stage 2: Using *k*-mer counting based on the variant sketch to determine the relationship between samples. The input includes the low-error-rate data used for sketch construction and other samples to be tested. The output is a CSV file listing relationships between samples.

Theoretically, heterozygous region distribution adheres to a Poisson or negative binomial distribution ranging from 0 to infinity [26]. However, directly selecting *k*-mers from these regions (i) greatly increases algorithm runtime and memory consumption and (ii) generates numerous false SNPs from chimeric sequences (overlapping *k*-mers in the SNP sequence actually originating from different reads) and homologous/repetitive sequences. Inspired by the Kmer2SNP concept, we heuristically designate heterozygous peaks of 0.5×–1.5× as heterozygous regions (minimum left boundary of 2 to exclude erroneous *k*-mers), ensuring the capture of most *k*-mers in these regions while maximally reducing runtime, memory usage, and false SNPs. Finally, we fitted the *k*-mer frequency distribution using 2 negative binomial distributions [25, 27] and provided a rough estimate of genomic characteristics to assist users in evaluating the correctness of the algorithm’s selection. The results of heterozygous region selection are shown in Fig. 2A and Supplementary Fig. S1.

After obtaining the *k*-mer data for the heterozygous regions, the next step is to call SNPs in these regions. KmerSNP first identifies *k*-mers within heterozygous regions that differ by only a single nucleotide in the middle and designates them as potential SNPs. It then expands both sides of each potential SNP, where the maximum possible expansion distance is defined as the support length. Finally, the algorithm selects the final SNPs by computing the maximum-weight matching of all potential SNPs (Fig. 2B).

Although it has demonstrated the best performance among reference-free SNP calling tools, we observed that for whole-genome SNP calling, its runtime often exceeds 1 hour, and the precision of SNP calling drops sharply as coverage decreases, which is unacceptable for our requirements. Excessive SNP calling time considerably increases the operational cost of using the tool, while a high number of incorrect SNP calls can severely impact our tool’s performance. To address these issues, we restructured and optimized the algorithm in C++, incorporating parallel-hash [28] for extensive parallel computation. To further enhance SNP calling precision, we selected only SNPs supported by a length of 21. Our improved Kmer2SNP algorithm only calls isolated SNPs, as these SNPs are independent in subsequent analyses. Since no reference genome is available, all called SNPs are heterozygous.

### Sketch construction

After SNP calling, a variant sketch FASTA file is constructed (Fig. 2B). Each called SNP is split into Ref and Alt columns and processed separately using a 21-mer sliding window. Each *k*-mer is hashed, and identical or reverse-complement *k*-mers are removed to ensure the independence. When validating relationships between multiple individuals in large cohorts, the process can be repeated for each individual, and the resulting sketches can be merged into a comprehensive sketch for analysis.

### Variant *k*-mer counting

After obtaining and reading the variant sketch FASTA file, we hash it into a hash table using a reversible hash function. Input sequences in FASTQ format are decomposed into *k*-mers, which are subsequently hashed. Whenever a *k*-mer matches an entry in the hash table, its read count is incremented by 1. These read counts are then used to compute the final score (Fig. 2C). Optionally, the process can be terminated early by specifying an expected coverage threshold to optimize runtime.

### Calculating score

We employed 2 methods to calculate the relatedness coefficient: relatedness score and likelihood score.The relatedness score provides a detailed measurement of the relationship between samples at higher coverage, while the likelihood score is designed to robustly verify whether 2 samples are identical under low-coverage conditions, primarily to detect sample swaps.

To calculate the relatedness score, allele counts at each site for each sample are first converted into genotypes. Each site includes multiple counts for reference (Ref) and alternative (Alt) alleles. The most frequent count is selected as the genotype for each site to obtain a reliable estimate. Next, the Ref/Alt ratio is calculated, then plotting a histogram to identify the cutoff point at the lowest frequency in the histogram. This cutoff is subsequently used to classify sites as heterozygous or homozygous (Fig. 2C).

After determining the genotype for each site, we calculate the relatedness score based on the differences in observed genotypes between each pair of samples. Existing methods, such as KING [13], rely on the IBS0 statistic, which represents the number of loci where a pair of individuals share zero alleles. For related individuals, such as parent-offspring or siblings, their IBS0 should never be zero unless Mendelian inheritance is violated. However, unlike typical scenarios, our variant sketch includes only heterozygous SNPs from each sample, with no information on homozygous SNPs. Therefore, we define the relatedness score calculation as follows:


(1) \begin{eqnarray*}
\frac{Het_{\mathrm{i}}}{Het_{\mathrm{i}}+Hom_{\mathrm{i}}}
\end{eqnarray*}

Here, $i$ represents the sample to be tested, while $\text{Het}_{\mathrm{i}}$ and $\text{Hom}_{\mathrm{i}}$ are the counts of heterozygous and homozygous sites for sample $i$, respectively. Sample $j$ is the reference sample used to create the variant sketch. We only observe the counts of sample $i$, as sample $j$ originates from the SNP calling of the target individual and consists entirely of heterozygous sites. In this context, if 2 samples are identical, sample $i$ should share all heterozygous SNPs with sample $j$, and thus the relatedness score is equal to 1. If there is a parent–offspring or sibling relationship between the samples, they should share half of the heterozygous SNPs, resulting in a relatedness score of 0.5. Due to the absence of homozygous SNP information, our tool is currently unable to detect more distant relationships.

Under low data coverage, distinguishing between heterozygous and homozygous genotypes becomes challenging. Therefore, we largely borrow from the exact method described in the ntsm publication. This method employs maximum likelihood estimation and log-likelihood ratio test to determine whether 2 samples are identical [29]. It assumes 2 models: one in which the samples are independent and another in which they are same. A multinomial-like likelihood function is used, and the log-likelihood ratio between the 2 models is calculated to provide a robust assessment of sample identity (Fig. 2C).

The key differences between our method and ntsm are as follows. First, in the likelihood ratio test, we introduce 2 samples: one is the query sample, while the other is modified such that all ref/alt values are set to 1. This approach incorporates a predefined distribution of identical samples, allowing for a comparative assessment of the query sample. Second, we remove the 2 empirical bias parameters in ntsm, and the final score is the mean log-likelihood ratio across all sites, as follows:


(2) \begin{eqnarray*}
{\overline{\lambda _{\mathrm{LR}}}=-2\log \frac{\mathcal {L} ^{\left( * \right)}}{\mathcal {L} ^{\left( 1 \right)}\cdot \mathcal {L} ^{\left( 2 \right)}}*\frac{1}{N}}
\end{eqnarray*}

Here, $\overline{\lambda _{\mathrm{LR}}}$ represents the mean of log-likelihood ratio test, $N$ represents the effective number of sites, and $\mathcal {L} ^{\left( 1 \right)}$ and $\mathcal {L} ^{\left( 2 \right)}$ represent the likelihood values of the 2 samples when considered independently, respectively. $\mathcal {L} ^{\left( * \right)}$ represents the likelihood value when considered identical. Additionally, it is noteworthy that we only consider sites where the sum of Ref and Alt values is greater than or equal to 2 (which we define as effective sites) to minimize the impact of missing data due to low coverage.

## Results

### Simulation

Simulations were conducted to investigate the impact of various factors on our method. We first assumed that the variant sketch contained 200k heterozygous sites. Then, we used a Poisson distribution to simulate the depth distribution under different coverage levels (0.1×, 0.2×, 0.3×, 0.5×, 1×, 2×, 3×, 4×, 6×, 8×, 10×, 20, 30×).

For the same individual, a binomial model with *P* = 0.5 was used to simulate all sites. For individuals with a first-degree kinship, half of the sites were simulated using the binomial model, while for the other half, the reference (ref) and alternate (alt) alleles were randomly assigned, with one placed at the simulated depth and the other set to zero.

**Figure 3:**
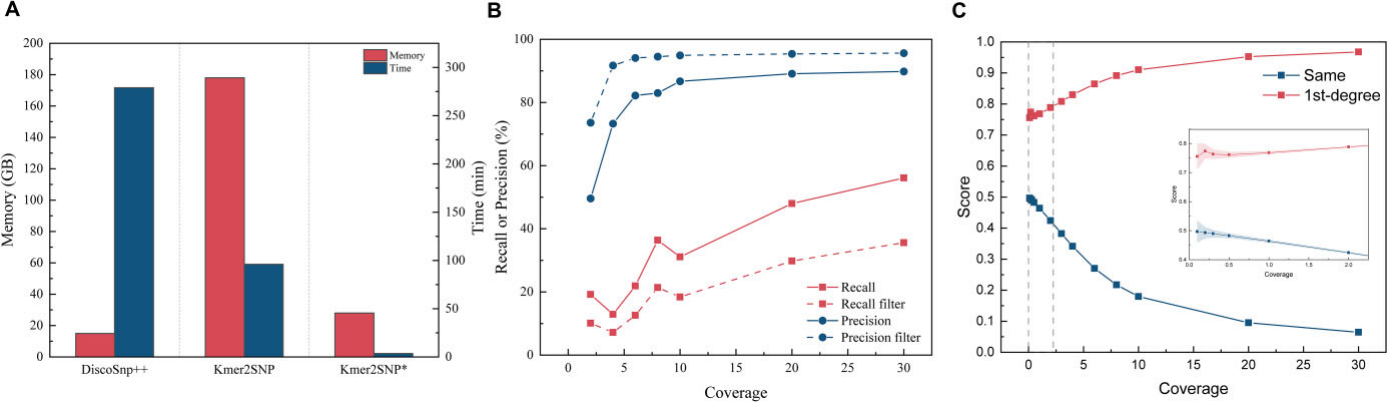
SNP calling and simulation results. (A) The performance of reference-free SNP calling tools. Each tool only calls isolated SNPs, with other settings following default parameters. DiscoSnp++ and Kmer2SNP* use 8 cores, while Kmer2SNP only supports 1 core. Kmer2SNP* only involves optimizations for time and memory consumption, and its SNP calling results remain consistent with Kmer2SNP. The time statistics do not include the *k*-mer counting step (DSK). (B) The results of SNP calling performance after filtering by selecting SNPs supported by a length of 21. (C) Sample swap scores based on simulations. Sequencing depth distribution is simulated using a Poisson distribution, while depth at each heterozygous site follows a binomial distribution with *P* = 0.5. The shaded areas around each line represent the 1st and 99th percentiles of the simulated scores.

Each coverage level was simulated 1,000 times, and the mean, 1st percentile, and 99th percentile values were recorded. As shown in Fig. 3C, the discriminability between the 2 cases improves with increasing coverage. Finally, we set a threshold score of 0.63 to determine whether the 2 samples are identical.

### SNP calling

We evaluated the performance of the improved Kmer2SNP using PacBio High-Fidelity (HiFi) sequencing data from the HG002 sample at a depth of 30×. As shown in Fig. 3A, our tool achieves considerably faster SNP calling, requiring only 3.5 minutes and 28 GB of memory. Compared to the original Kmer2SNP algorithm, this achieves a 25.2-fold increase in speed and a 6.3-fold reduction in memory consumption. Although DiscoSnp++ benefits from using a Bloom filter, which reduces its memory usage to around 15 GB, its runtime of nearly 4.65 hours makes our tool a more efficient choice.

Subsequently, we analyzed the selection of *k*-mer sizes from 2 perspectives. For SNP calling performance, increasing *k* enhanced overall performance, although it also raised memory usage and runtime. Starting at 21-mer, it achieved high-quality results, which then showed gradual improvement as *k* increased further (Supplementary Table S1). In terms of tolerance to high-error data in stage 2, smaller *k*-mers provide greater redundancy to compensate for sequence errors (Fig. 2C). To balance these factors, we chose a 21-mer, which achieves sufficient SNP calls, shorter runtime, and enhanced performance with high-error-rate data.

Finally, we evaluated SNP calling results across different coverages by using seqkit (RRID:SCR_018926) [30] to subsample HG002 PacBio HiFi sequencing data to the depth of 2×, 4×, 6×, 8×, 10×, 20×, and 30×. Figure 3B shows that both recall and precision improve as coverage increases. However, we found that the precision of SNP calling was insufficient, with a large number of erroneous calls occurring at low coverage levels, especially below 10×. After filtering by selecting only SNPs supported by a length of 21, precision remained above 90% for coverages of 4× and higher, at the cost of some recall.

### Impact of SNP sites

**Figure 4:**
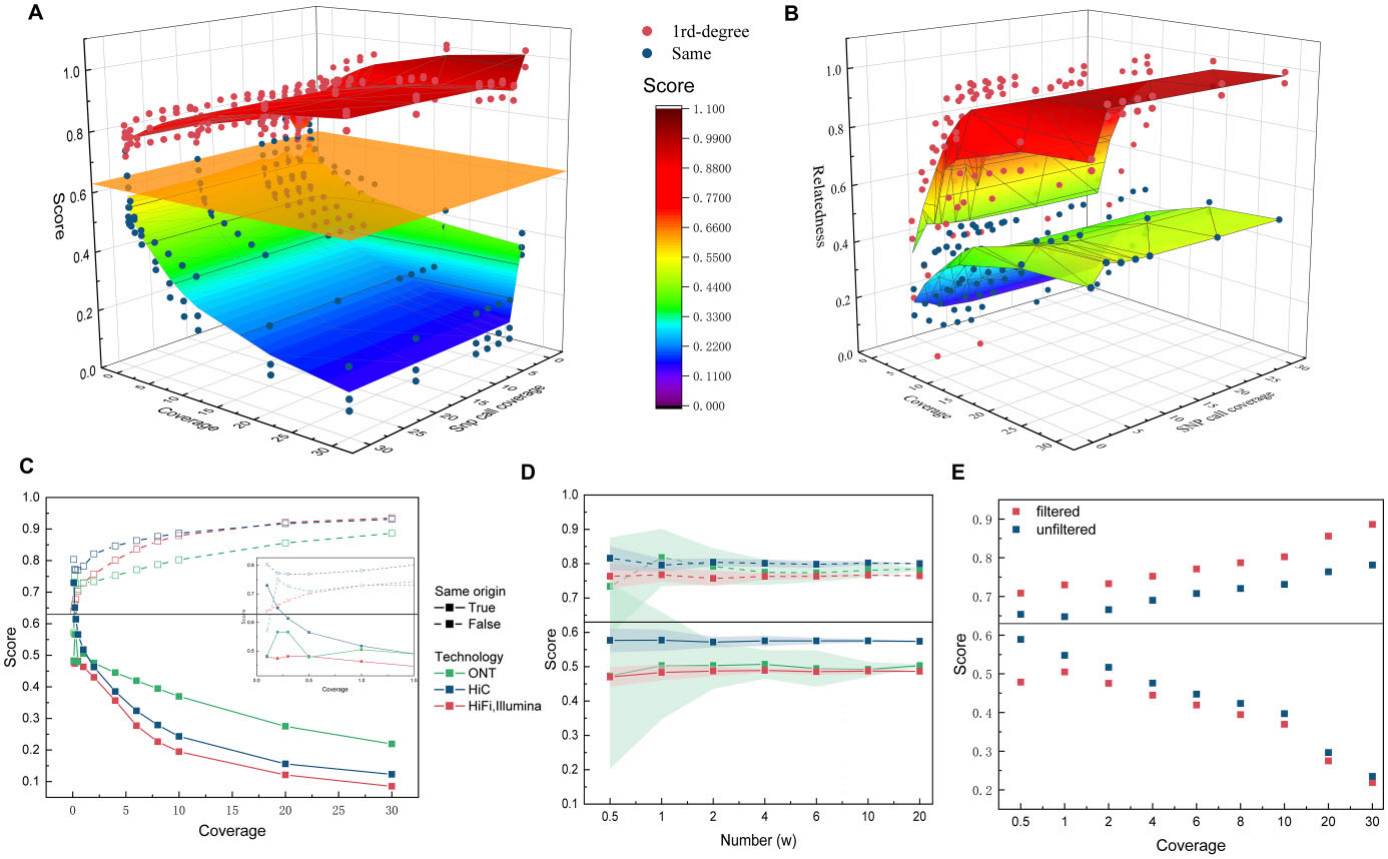
The impact of data coverage. (A) Likelihood score results for different input and SNP calling data coverages. Blue and red points indicate results for the same and different samples. The x-axis represents the coverage of the data being tested, while the y-axis denotes SNP call coverage, referring to the coverage used for SNP calling and variant construction. The orange plane represents the threshold (t = 0.63) used by our method to determine whether samples are identical. (B) Relatedness score results under the same conditions as (A). Blue and red points represent results for first-degree relationships and identical relationships, respectively. (C) Cross section of Fig. 4A with the SNP call coverage axis at a value of 4, where different colors represent different sequencing technologies. (D) The variation in sample swap scores under different numbers of heterozygous SNPs. Each experiment is simulated 10 times, and the shaded area around each line represents the range between the maximum and minimum scores. (E) The results of detecting sample swaps on filtered and unfiltered sketches. The horizontal line in the figure represents the threshold for determining whether samples are identical. To achieve better discrimination, the 2 categories of samples should be as far from the line as possible.

To evaluate the impact of increased precision and reduced recall on SNP calling results after filtering, we first conducted experiments using 4× coverage SNP calls and ONT (R9.4.1, accuracies: 85.6%, 87.7%) data from HG002 and HG003. We chose ONT data due to its high error rate, anticipating that it would present the greatest analytical challenge. As shown in Fig. 4E, the filtered variant sketch displays marked enhanced discriminatory power compared to the unfiltered version, enabling the tool to make more robust assessments.

To further investigate the impact of SNP sites used in stage 1 on the results, we used the previously mentioned HG002 PacBio HiFi data with 2×–30× coverage to create the variant sketch. Meanwhile, we selected downsampled data from HG002 and HG003, including PacBio HiFi, Hi-C, and ONT (R9.4.1) sequencing technologies, with coverage ranging from 0.1× to 30×, as the test data for stage 2.

In the data coverage utilized for SNP calling, we found that neither the likelihood nor the relatedness score is particularly sensitive to coverage levels in stage 1 (Fig. 4A, B). The only substantial performance drop occurred at 2× coverage. As shown in Fig. 3B, 2× coverage is also the only condition where precision falls below 90%, suggesting that an excess of erroneous SNP calls reduces tool performance. For coverages above 2×, although the number of SNP calls increased, this increase provided minimal benefit to tool performance compared with the importance of maintaining high precision.

Meanwhile, we further investigated the number of SNPs used in the variant sketch in stage 1. From all heterozygous SNPs called at 30× coverage in HG002, we randomly sampled 5k, 10k, 20k, 40k, 60k, 100k, and 200k SNP sites, corresponding to 0.25%, 0.5%, 1%, 2%, 3%, 5%, and 10% of the total heterozygous SNPs in HG002. Subsequently, we tested the data from different sequencing technologies at 0.5× coverage. For each case, we performed 10 repetitions and recorded the mean, minimum, and maximum values of the score. As shown in Fig. 4D, the stability of the variant sketch for low-coverage data decreases as the number of SNPs decreases, particularly for ONT data. We attribute this to its high error rate, which significantly reduces the number of effective sites. Therefore, we conservatively estimate that at least approximately 20k sites are required to maintain basic stability, ensuring at least 50 effective sites even for ONT data at a coverage as low as 0.5$\times$. When the number of effective sites falls below 50, we issue an unreliability warning, urging the reselection of more SNP sites for testing. Coincidentally, we observed that tools like NGSCheckMate [9] and somalier [10] also utilize approximately 20,000 SNP sites to assess sample relationships, further underscoring the significance of this site count.

### Impact of stage 2 coverage

In contrast to the minimal impact of stage 1 coverage, we found that data coverage in stage 2 has a considerable effect on the tool’s performance. As coverage increases, the tool’s ability to measure relationships improves accordingly. For calculating relatedness score to assess the relationship between 2 samples, we recommend a minimum coverage of 20× to ensure robust estimates (Fig. 4B). However, if the objective is simply to calculate the likelihood score to determine whether the 2 samples are identical, a much lower coverage level is sufficient. Concurrently, we noted that ONT data posed the greatest challenge in detecting sample swaps, as its elevated error rate diminishes effective site counts, requiring increased coverage for enhanced differentiation. Additionally, we found that the tool performs well across various sequencing data types at coverage levels above 0.5× (Fig. 4A, C).

### Comparisons to ntsm

To evaluate the performance of our tool, we selected well-studied human samples with extensive and reliable reference information. This choice enabled us to compare our tool with the state-of-the-art reference-based sample swap detection tool, ntsm.

To validate sample swaps, we used 20 samples from the Human Pangenome Reference Consortium (HPRC) [31], which includes sequencing data from Illumina, PacBio HiFi, Hi-C, and ONT (R9.4.1) platforms. We then constructed the variant sketch using Illumina data from each sample, while the remaining data were downsampled to coverage levels ranging from 0.1× to 20× to evaluate the performance of our tool and ntsm under varying coverage levels. Details of these datasets are provided in Table 1 and Supplementary Table S2. Additionally, to calculate relatedness, we supplemented this dataset with samples from the 1000 Genomes Project [32], combining it with HPRC sample to create a dataset of 20 family trios (Supplementary Table S1). Each child in the trios has data from multiple sequencing technologies, while the parents have only Illumina data.

#### Detecting the sample swap

**Figure 5:**
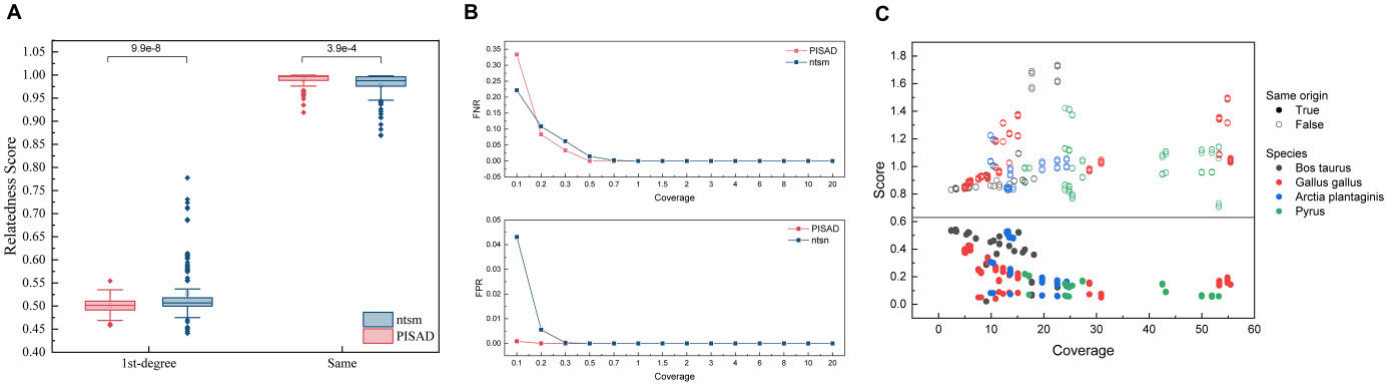
Evaluation results on human and other species. (A) Relatedness score results of ntsm and PISAD for 20 human family trio samples. Levene’s test is used to perform significance analysis on the variance of the data. (B) False -egative rate (FNR) and false -ositive rate( FPR) of PISAD and ntsm at varying raw dataset coverage in 20 human samples. PISAD constructs variant sketches using approximately 15× coverage Illumina data for each sample. ntsm uses the lower coverage between the 2 compared samples as the x-axis value. (C) Results of identifying identical samples in trio data across different species. The x-axis represents the coverage of the sample with the lower coverage between the 2 input samples. The gray horizontal line denotes the threshold for determining sample identity.

We evaluated the performance of both tools across different data coverage levels. We found that both tools could detect samples with coverage higher than 1×. However, our tool was able to further detect samples with coverage as low as 0.5× (Fig. 5B and Supplementary Fig. S3). Not surprisingly, as noise begins to dominate the correlation, performance declines at very shallow depths (< 0.5×). We infer that although our method lacks homozygous SNP sites, which provide better discriminative power in shallow sequencing [33], the sample-specific sites generated by our approach result in a greater number of effective sites compared to the universal sites selected by ntsm. As a result, this leads to improved performance.

#### Calculating relatedness

For the relatedness score, we found that our tool demonstrated tighter grouping compared to ntsm (Fig. 5A). Further analysis revealed that most of the poorly estimated results in ntsm were derived from ONT sequencing, likely due to its high error rates. Our method differs from ntsm in 2 key aspects. First, when merging multiple counts at each site, we use the mode instead of the maximum value. For example, if we observe counts like 8, 9, 8, and 7 at a given site, we take the mode (which would be 8) rather than the maximum (which would be 9). Second, instead of using a fixed threshold to determine the genotype, we determine the threshold by identifying the minimum value of the Ref/(Ref + Alt) spectrum within a certain range (Fig. 2C). We believe these modifications help mitigate the impact of high error rates, leading to a more stable and compact genotype distribution. However, due to the absence of homozygous SNP information in our method, it lacks the capability to distinguish relationships beyond first-degree (parent–offspring and siblings), unlike reference-based tools.

### Extension to other species

To assess the performance of our tool on other species in practical application scenarios, we selected family trio samples from 3 species: *Bos taurus, Gallus gallus, Arctia plantaginis*, and *Pyrus*. The first 2 species were obtained from the Vertebrate Genome Project (VGP) [34], while the third was obtained from a study that performed *de novo* assembly of *A. plantaginis* through trio binning [1]. The last one is derived from a study on Telomere-to-Telomere (T2T) genome assembly of 2 pear hybrid cultivars, “Yuluxiang” (YLX) and “Hongxiangsu” (HXS) [35]. Details of these datasets are provided in Table 1.

**Table 1:** Detailed information on the sample swap experiments for each species

Species^a^	Tissue	Sequencing technology^b^	Depth	Individual	Variant sketch^c^	Sample	Match, unmatch^d^
*Homo sapiens* (0.31%)	Lymphoblastoid cell lines	Illumina, PacBio HiFi, Hi-C, ONT (R9.4.1, 91.1%)	0.1–20×	20	20	840	833, 15,967
*Bos taurus* (1.12%)	Frozen lung	Illumina, Hi-C, ONT (R9.4.1, 85.2%)	2.4–22.6×	3	6	26	52, 104
*Gallus gallus* (1.03%)	Blood	Illumina, Pacbio HiFi, Hi-C, 10× Genomics	4.9–55.4×	3	18	40	230, 490
*Arctia plantaginis* (1.90%)	Pupae	Illumina, PacBio CLR, 10× Genomics	9.8–24.3×	3	12	17	112, 88
*Pyrus* (1.6%, 2.0%)	Leaf	Illumina, PacBio HiFi, ONT (R9.4.1, 93.7%)	17.1–53.2×	5	10	18	47, 133

a The values in parentheses represent the estimated heterozygosity, which was estimated using GenomeScope2 [26] based on offspring sequencing data with coverage exceeding 30×. For Purus, 2 separate estimates were obtained due to the presence of 2 offspring.

b The values in parentheses represent the flow cell version and the average accuracy of the ONT data.

c Represents the number of data used to construct the variant sketch, which includes all Illumina data for humans and all low-error-rate data for other species.

d Represents the number of matching and nonmatching experiments in the total number of experiments, where the total number of experiments is equal to the number of variant sketches multiplied by the number of query samples.

For each species, we first created sketches on all low-error-rate runs. We then validated all data runs against each sketch to evaluate the performance of our tool in real-world scenarios. As shown in Fig. 5C, for species with different heterozygosity, such as *B. taurus, G. gallus, A. plantaginis*, and *Pyrus*, our tool is able to identify all identical and nonidentical samples correctly. Additionally, as coverage increased, the results became more confident.

### Running time

Since a large number of files may need to be checked, the speed of the algorithm is crucial. In stage 1, the time consumption mainly stems from 2 processes: (i) *k-*mer histogram counting, which uses the DSK algorithm to extract *k*-mers located in heterozygous regions, and (ii) calling algorithm, which performs SNP calling based on these filtered *k*-mers. The time required for *k*-mer histogram counting increases with the amount of data (Supplementary Fig. S4A). For the calling algorithm, memory and time consumption largely depend on the number of heterozygous-region *k*-mers identified in the *k*-mer histogram counting phase. At lower data depths, the boundaries of heterozygous regions are less well defined, which introduces a large amount of irrelevant data and increases both time and memory consumption. However, this improves with deeper coverage. In stage 2, we observed that the runtime increases almost linearly with the depth of data (Supplementary Fig. S4B). Additionally, it supports parallel processing of multiple files.

For a typical run of low-error-rate sequencing data, the coverage is approximately 10×. Under these conditions, the total time for stage 1 is roughly 10 minutes. Additionally, our performance evaluation shows that comparable results can be achieved with coverage as low as 0.5x× In this case, the computation time for a single sample in stage 2 is approximately 2 minutes. Therefore, testing a pair of samples requires only 12 minutes.

## Discussion

### Comparisons to reference-free tools

Although our tool, like generic *k*-mer comparison methods such as Mash, does not require reference information, there are significant differences in the workflow. Mash operates by extracting a subset of sequences with some of the smallest hash values from the data for comparison. In contrast, our method essentially extracts SNP information from the data for comparison. This difference leads to 2 key distinctions. First, thanks to the use of SNP information, our method can identify intraspecies sample swaps, whereas Mash is generally limited to distinguishing samples between distinct species. However, our approach is also constrained by the requirements of SNP extraction, which necessitates low-error-rate data with sufficient coverage, even if we can avoid performing SNP calling for each sample by creating sketches. On the other hand, Mash has almost no requirements on the data, making it compatible with various data types. Additionally, in terms of computation, extracting a subset of sequences and performing statistics on them is simpler than performing SNP calling, indexing, and comparing sequences between samples.

### Comparisons to reference-based tools

The primary difference between our tool and reference-based methods like ntsm is that we replace the need for predefined variant sites with a step of reference-free SNP calling and variant sketch construction, allowing our tool to be applied to multiple diploid species rather than being limited to human. According to our performance evaluation, we achieved a lower data coverage requirement compared to ntsm, requiring only 0.5× coverage. However, this comes at the cost of requiring additional high-accuracy sequencing data for constructing the variant sketch.

In terms of time consumption, our method generally performs similarly to ntsm. The main difference is the additional step in stage 1, where SNP calling and sketch construction take place, which can be completed in about 10 minutes. Additionally, unlike stage 2, where *k*-mer counting is required for each sample, to validate a batch of samples and check if they belong to the same individual, we only need one low-error-rate data from the target individual for SNP calling and variant sketch construction.

### Current limitations

In stage 1 of our tool, although we have optimized the reference-free SNP calling algorithm to better accommodate low-coverage data and achieve faster SNP calling, its memory consumption, which often exceeds 40 GB, remains challenging for small-memory servers. Additionally, our SNP calling method is still only suitable for low-error-rate data. Since our approach relies solely on *k*-mers rather than alignment to a reference genome, it has difficulty distinguishing between base errors and true base variants. This limitation means that at least 1 low-error-rate data from the target individual is required to use our tool effectively. Regarding the scope of application, our method primarily focuses on detecting sample swaps in WGS data and has not yet been tested on other data types such as whole-exome data [36], RNA sequencing [37], or chromatin immunoprecipitation sequencing [38]. While we are optimistic about the principle behind it (i.e., the use of SNP information), variations in sequencing regions, such as reduced-representation sequencing, may require a greater number of SNP sites to ensure the tool’s effectiveness. Meanwhile, calling SNPs from such data without reference poses greater challenges to precision compared to WGS data. Due to the absence of designated heterozygous regions, the *k*-mer–based method resulted in a higher incidence of false SNPs from homologous/repetitive sequences, yielding a precision of only 70%–80%. This will also constrain the tool’s resolution capability, warranting further investigation. Additionally, our current study is exclusively focused on diploid species. Polyploid organisms have not been included in this investigation due to the inherent challenges in SNP calling associated with their complex genomic architectures, which warrant separate and more extensive future research.

## Conclusions

We have developed PISAD to detect intraspecies sample swaps in heterogeneous data cohorts without reference information and have demonstrated its effectiveness in multiple diploid species. It achieves excellent performance even for datasets with sequencing depths as low as 0.5× and with multiple sequencing technologies. We believe that our tool, which neither requires additional reference information nor downstream analyses like alignments, can be easily integrated into upstream data production pipelines as an efficient QC process.

## Availability of Source Code and Requirements

- Project name: PISAD
- Project homepage: https://github.com/ZhantianXu/PISAD
- Operating system(s): linux
- Programming language: C++, Python
- Other requirements: Conda
- License: MIT
- BiotoolsID: pisad
- RRID: SCR_026597
- Software Heritage PID:
  swh:1:snp:273180d6cf73f0ab496baa7d9ea7d719a213e175 [39]

## Supplementary Material

giaf061_Supplemental_Files

giaf061_Authors_Response_To_Reviewer_Comments_original_submission

giaf061_Authors_Response_To_Reviewer_Comments_revision_1

giaf061_GIGA-D-24-00517_original_submission

giaf061_GIGA-D-24-00517_Revision_1

giaf061_GIGA-D-24-00517_Revision_2

giaf061_Reviewer_1_Report_Original_submission Jim Shaw -- 12/11/2024

giaf061_Reviewer_2_Report_Original_submission Miles Roberts -- 1/23/2025

giaf061_Reviewer_2_Report_Revision_1 Miles Roberts -- 4/15/2025

## Data Availability

The specific workflow of this work [41] can be found at WorkflowHub. The Illumina, PacBio HiFi, Hi-C, and ONT sequencing data for 20 human family trios are available at Amazon S3 under the HPRC/ and HPRC_PLUS/ directories [42]. Details of the selected samples are shown in Supplementary Table S2. For the coverage experiments, the Illumina, PacBio HiFi, and ONT sequencing data for HG002 and HG003 are available at the National Center for Biotechnology Information (NCBI) [43]. The Hi-C data for HG002 can be accessed at Amazon S3 [44]. The data for *Bos taurus, Gallus gallus*, and *Arctia plantaginis* are available from NCBI under the following project accession numbers: PRJNA677946, PRJNA1149711, PRJNA1150343, and PRJEB36595. The Pyrus data are available in the National Genomics Data Center under BioProject accession PRJCA022120 with CRA accession CRA013997. All additional supporting data are available in the *GigaScience* repository, GigaDB [45].

## Additional Files

**Supplementary Fig. S1**. Schematic diagram of heterozygous region selection for *Homo sapiens* and *Arctia plantaginis* at 2×, 4×, and 6× coverage.

**Supplementary Fig. S2**. Heterozygous SNP calling results by DiscoSNP for HG002 PacBio HiFi sequencing data across varying coverage levels.

**Supplementary Fig. S3**. Detailed results of the sample swap scores for 20 human samples. Each sample includes sequencing data from 3 technologies: Hi-C, HiFi, and ONT. The horizontal line in the figure represents the threshold for determining whether the samples are identical, set at 0.63.

**Supplementary Fig. S4**. The time and memory consumption of the PISAD. Experiments are conducted on a platform with an Intel Xeon Gold 6348 CPU @ 2.60 GHz. (A) The memory and time consumption for reference-free SNP calling with different sequencing depths in stage 1 (using 8 core). The memory consumption during the *k*-mer histogram counting phase depends on the maximum memory setting configured for DSK, which is set to 30 GB here. (B) The time consumption for *k*-mer counting on the sketch with different sequencing depths in stage 2 (using 1 core). The memory consumption is independent of the coverage and mainly depends on the size of the sketch, which is approximately 0.5 GB in this case.

**Supplementary Table S1**. The performance of SNP calling under different *k*-mer sizes (HG002 PacBio HiFi data at 30× coverage).

**Supplementary Table S2**. The IDs of 20 human family trios form the HPRC and 1000 Genome Project.

## Abbreviations

Alt: alternative alleles; bp: base pair; eBWT: extended Burrows–Wheeler transform; FNR: false-negative rate; FPR: false-positive rate; HPRC: Human Pangenome Reference Consortium; MLE: maximum likelihood estimation; ONT: Oxford Nanopore Technology; QC: quality control; Ref: reference alleles; SNP: single nucleotide polymorphism; T2T:Telomere-to-Telomere; tsv: tab-separated values; VCF: variant call format; VGP: Vertebrate Genome Project; WGS: whole-genome sequencing studies.
