# Supplementary material for: PISAD: reference-free intraspecies sample anomalies detection tool based on k-mer counting
Source: Gigascience. 2025 Jun 17;14:giaf061. doi: 10.1093/gigascience/giaf061 (PMC12202988; doi:10.1093/gigascience/giaf061)
Supplement: giaf061_Supplemental_Files [file giaf061_supplemental_files.zip › supplement tables.PDF]

Table S1. The performance of SNP calling under different *k*-mer sizes(HG002 PacBio HiFi data at 30x coverage)

k-mer	Precision(%)	Recall(%)	Memory(GB)	Time(s)
17	89.1	3.5	18.8	94
19	90.3	35.1	30.1	186
21	89.8	56.1	28.2	209
23	89.6	60.7	28.8	212
25	89.7	62.4	29.1	192
27	89.7	63.9	28.7	195
29	89.8	64.9	54.8	233
31	89.8	65.9	54.3	294

Table S2. The IDs of 20 human family trios form the HPRC and 1000 Genome Project

Child	Parents	
HG00438	HG00436	HG00437
HG00621	HG00619	HG00620
HG00673	HG00671	HG00672
HG00733	HG00731	HG00732
HG00735	HG01047	HG00734
HG01258	HG01256	HG01257
HG01361	HG01359	HG01360
HG01891	HG01890	HG01889
HG01928	HG01926	HG01927
HG01952	HG01950	HG01951
HG01978	HG01977	HG01976
HG02055	HG02053	HG02054
HG02080	HG02081	HG02082
HG02630	HG02628	HG02629
HG02717	HG02715	HG02716
HG02886	HG02884	HG02885
HG03492	HG03490	HG03491
HG03516	HG03515	HG03514
HG03540	HG03538	HG03539
HG03098	HG03096	HG03097
