# Supplementary figures and images for: PISAD: reference-free intraspecies sample anomalies detection tool based on k-mer counting
Source: Gigascience. 2025 Jun 17;14:giaf061. doi: 10.1093/gigascience/giaf061 (PMC12202988; doi:10.1093/gigascience/giaf061)

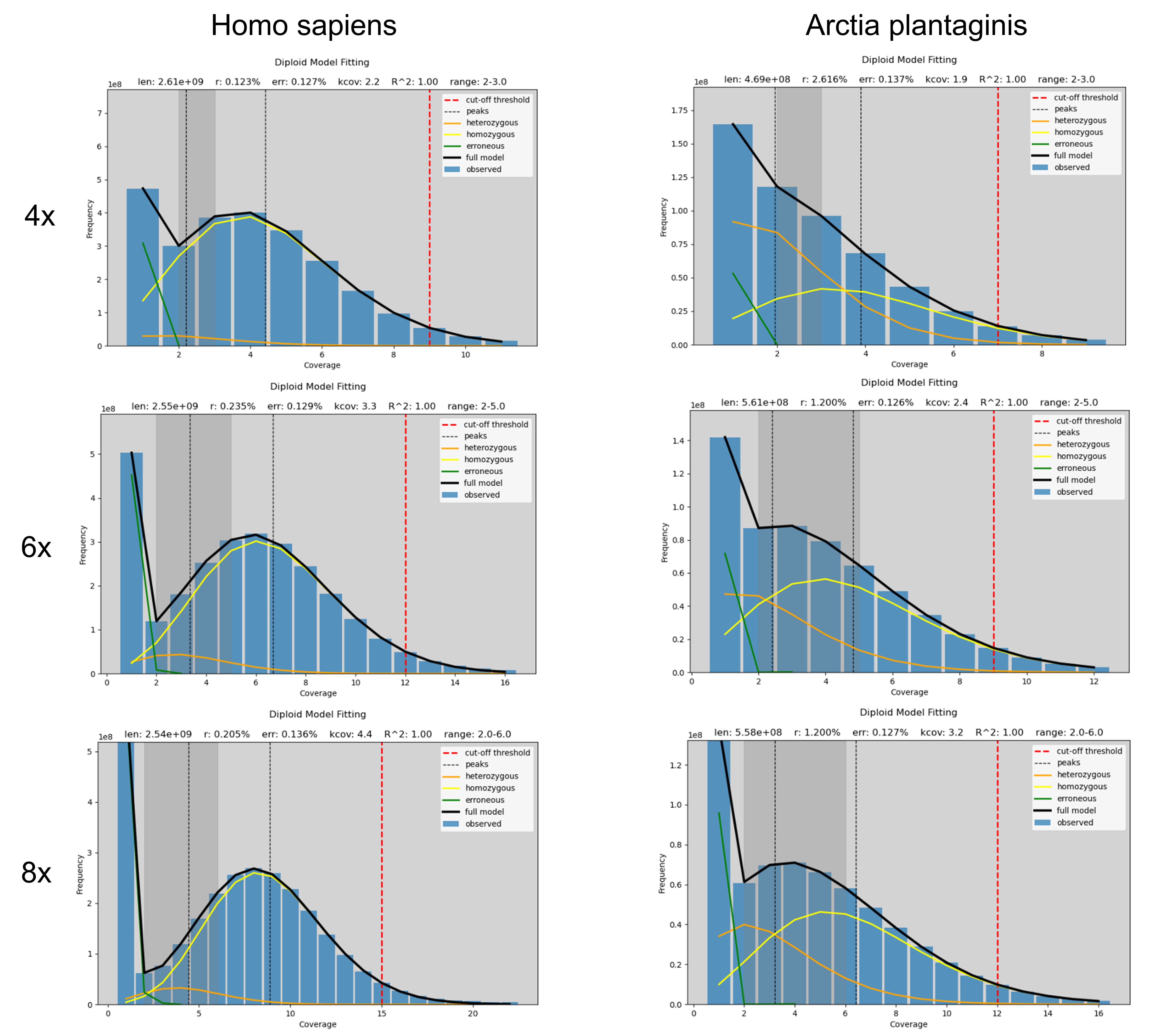

Supplement: giaf061_Supplemental_Files [file giaf061_supplemental_files.zip › figs1.png]

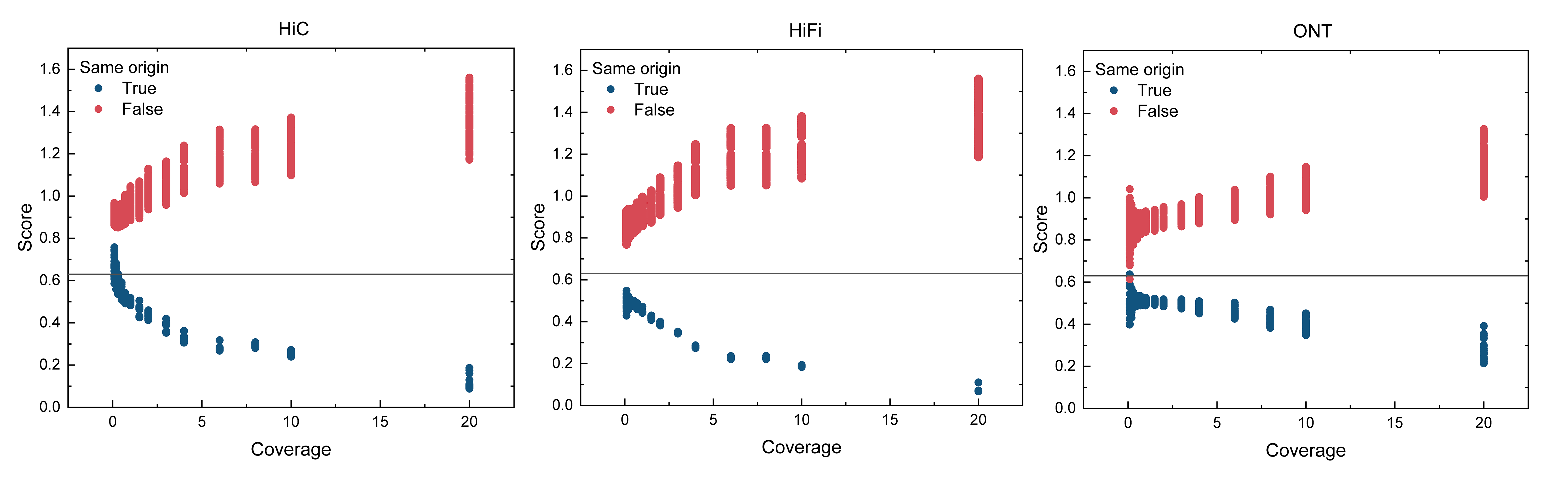

Supplement: giaf061_Supplemental_Files [file giaf061_supplemental_files.zip › figs2.png]

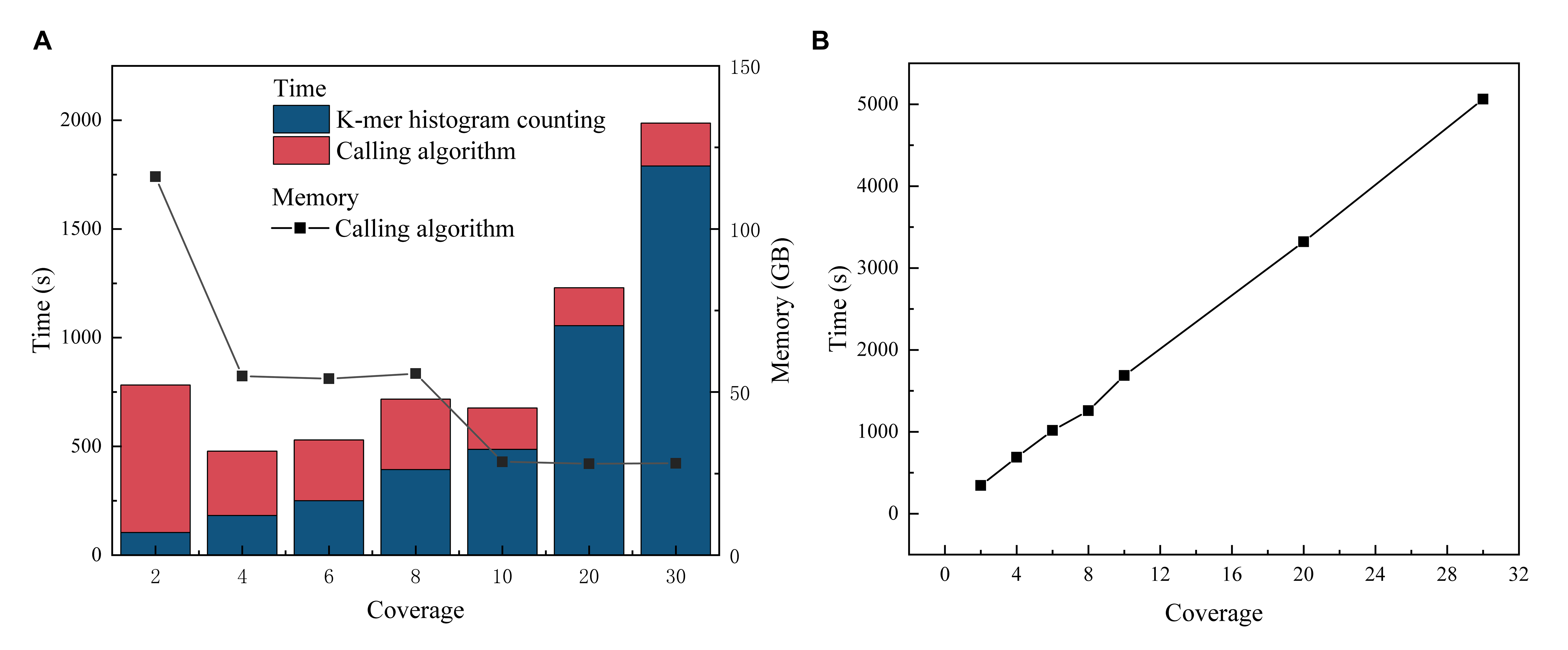

Supplement: giaf061_Supplemental_Files [file giaf061_supplemental_files.zip › figs3.png]

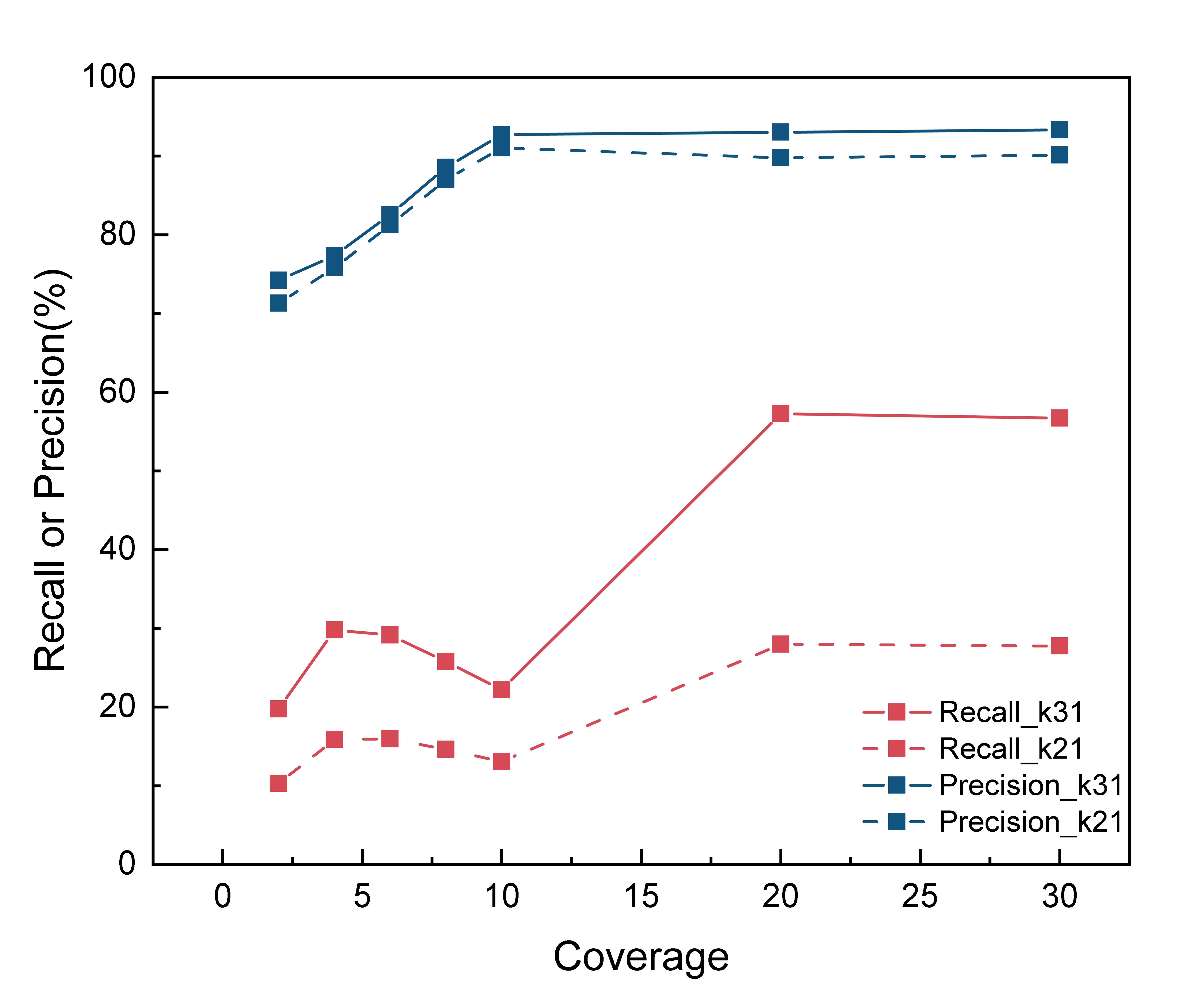

Supplement: giaf061_Supplemental_Files [file giaf061_supplemental_files.zip › figs4.png]
