# Supplementary material for: PISAD: reference-free intraspecies sample anomalies detection tool based on k-mer counting
Source: Gigascience. 2025 Jun 17;14:giaf061. doi: 10.1093/gigascience/giaf061 (PMC12202988; doi:10.1093/gigascience/giaf061)
Supplement: giaf061_GIGA-D-24-00517_original_submission [file giaf061_giga-d-24-00517_original_submission.pdf]

GigaScience

PISAD: reference-free intraspecies sample anomalies detection tool based on k-mer counting

--Manuscript Draft--

**Manuscript Number:**	GIGA-D-24-00517
**Full Title:**	PISAD: reference-free intraspecies sample anomalies detection tool based on k-mer counting
**Article Type:**	Research
**Funding Information:**	National Natural Science Foundation of China (62332020) — Prof. Jianxin Wang
**Abstract:**	Background: Genomic sequencing research often requires the simultaneous analysis of heterogeneous data types across single or multiple individuals, introducing a substantial risk of sample swaps (e.g., labeling errors). Existing methods primarily rely on reference information, requiring the pre-selection of informative variant sites with a population allele frequency around 0.5,<br>which may be insufficient or unavailable for non-model organisms. As research expands to encompass a growing number of new species, a robust quality control tool will become increasingly important.<br>Finds: We developed PISAD, a tool for verifying sample identities directly fromsequencing data. It uses a two-stage approach: first, it performs rapid, reference-free SNP calling on low-error-rate data fromthe target individual to create a variant sketch; then, it assesses the concordance of other samples on this sketch to verify relationships. We assessed the performance and efficiency of<br>PISAD on Homo sapiens, Bos taurus, Gallus gallus and Arctia plantaginis species.<br>Conclusion: Our evaluation showed that PISAD achieves performance comparable to reference-based tools even at coverage as low as 2x and is broadly applicable to diploid species with low heterozygosity.
**Corresponding Author:**	Jianxin Wang<br>Central South University<br>Changsha, -Select- CHINA
**Corresponding Author Secondary Information:**	
**Corresponding Author's Institution:**	Central South University
**Corresponding Author's Secondary Institution:**	
**First Author:**	Zhantian Xu
**First Author Secondary Information:**	
**Order of Authors:**	Zhantian Xu
	Fan Nie
	Jianxin Wang
**Order of Authors Secondary Information:**	
**Additional Information:**	
**Question**	**Response**
Are you submitting this manuscript to a special series or article collection?	No
**Experimental design and statistics**	Yes

Powered by Editorial Manager® and ProduXion Manager® from Aries Systems Corporation

Full details of the experimental design and statistical methods used should be given in the Methods section, as detailed in our Minimum Standards Reporting Checklist. Information essential to interpreting the data presented should be made available in the figure legends.<br><br>Have you included all the information requested in your manuscript?	
**Resources**<br><br>A description of all resources used, including antibodies, cell lines, animals and software tools, with enough information to allow them to be uniquely identified, should be included in the Methods section. Authors are strongly encouraged to cite Research Resource Identifiers (RRIDs) for antibodies, model organisms and tools, where possible.<br><br>Have you included the information requested as detailed in our Minimum Standards Reporting Checklist?	Yes
**Availability of data and materials**<br><br>All datasets and code on which the conclusions of the paper rely must be either included in your submission or deposited in publicly available repositories (where available and ethically appropriate), referencing such data using a unique identifier in the references and in the "Availability of Data and Materials" section of your manuscript.<br><br>Have you have met the above requirement as detailed in our Minimum Standards Reporting Checklist?	Yes
GigaScience has policies and guidelines in place for the use of generative AI-writing tools such as ChatGPT. If you have used such writing tools to assist with	Yes

writing the manuscript this must be declared and cited in the text. Authors should not list AI-writing tools and other AI-assisted technologies as an author or co-author and should acknowledge that they are fully responsible for text generated or refined by AI-writing tools.<p><br><br>A summary of use (particularly in the introduction or among methods) needs to be included at the end of the paper, and the outputs should also be included as a supplementary file hosted in GigaDB or other open repositories. Please read our guidelines for more information. <p><br><br>By submitting to GigaScience, you are aware of the journal's AI-writing tools policy, and if you have declared use of such tools below, you have acknowledged this where appropriate in your manuscript and have made a summary of use and outputs available. <p><br><b>AI-assisted writing tools have been used in the preparation of this manuscript?	

Manuscript Click here to access/download;Manuscript;PISAD.pdf

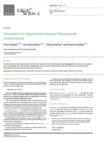

GigaScience, 2023, 1–9

doi: xx.xxxx/xxxx
Manuscript in Preparation
Paper

PAPER

PISAD: reference-free intraspecies sample anomalies detection tool based on k-mer counting

Zhantian Xu[1], Fan Nie[2] and Jianxin Wang[1,*]

[1]School of Computer Science and Engineering, Central South University, Changsha 410083, China and [2]National Center for Applied Mathematics in Hunan and Key Laboratory of Intelligent Computing and Information Processing of Ministry of Education, Xiangtan University, Xiangtan 411105, China

[*]Jianxin Wang, School of Computer Science and Engineering, Central South University, Changsha 410083, China. E-mail: jxwang@mail.csu.edu.cn

Abstract

Background: Genomic sequencing research often requires the simultaneous analysis of heterogeneous data types across single or multiple individuals, introducing a substantial risk of sample swaps (e.g., labeling errors). Existing methods primarily rely on reference information, requiring the pre-selection of informative variant sites with a population allele frequency around 0.5, which may be insufficient or unavailable for non-model organisms. As research expands to encompass a growing number of new species, a robust quality control tool will become increasingly important.
Finds: We developed PISAD, a tool for verifying sample identities directly from sequencing data. It uses a two-stage approach: first, it performs rapid, reference-free SNP calling on low-error-rate data from the target individual to create a variant sketch; then, it assesses the concordance of other samples on this sketch to verify relationships. We assessed the performance and efficiency of PISAD on *Homo sapiens*, *Bos taurus*, *Gallus gallus* and *Arctia plantaginis* species.
Conclusion: Our evaluation showed that PISAD achieves performance comparable to reference-based tools even at coverage as low as 2x and is broadly applicable to diploid species with low heterozygosity.

Key words: Sample swap, SNP calling, Reference-free, K-mer analysis, Quality control.

Introduction

Whole-genome sequencing (WGS) studies often involve multiple or single individuals across various experiments using different sequencing technologies(e.g., Illumina, PacBio, Oxford Nanopore Technologies, Hi-C, etc.). For instance, de novo assembly often involves sequencing data from multiple technologies[1, 2, 3] to improve assembly quality. Moreover, sequencing data from each technology may involve multiple sequencing runs. Each new procedure or handling introduces potential opportunities for sample swap. Even a single sample swap can have severe consequences on downstream analyses. Therefore, confirming the relatedness of samples assumed to come from the same donor is an essential step in quality control(QC), which should be performed as early as possible in the analysis pipeline.

Existing sample swap detection methods can be divided into two categories based on the source of the sample: cross-species and same-species. Cross-species swaps have been extensively studied. For example, Mash[4] uses MinHash techniques to rapidly calculate the genomic distance to identify them. However, in same-species swaps, the high genetic similarity among samples can obscure the differences. Current approaches for detecting same-species swaps primarily rely on genotypes at single nucleotide polymorphisms (SNPs), leveraging predetermined variant sites constructed from population-level allele frequency to distinguish between samples[5, 6, 7, 8, 9, 10, 11, 12]. For instance, Peddy[6] extracts genotypes at preselected variant sites from VCF files for each sample and uses the kinship calculation method from KING[13] to determine the relationships between samples. At the same time, Somalier[10] accelerates relationship calculation between samples by creating sketches for rapid comparison. NGSCheckMate[9] verifies sample identity in next-generation sequencing(NGS) data by

Compiled on: November 13, 2024.
Draft manuscript prepared by the author.

calculating the variant allele fractions at preselected SNP sites using a model-based approach. CrossCheck[8] leverages linkage disequilibrium to achieve improved accuracy in shallow sequencing. ntsm[11] leverages k-mer counting and maximum likelihood estimation, making it suitable for low-coverage and heterogeneous whole-genome sequencing data.

While current approaches for detecting sample swaps have been successful across a wide range of applications, most are limited to human samples. Although ntsm introduces a method for extracting informative variant sites and optimistically suggests applicability to other species, its performance may degrade or even fail in species where research is still in its early stages and population-level allele frequency information is limited or unavailable.

To address this problem, we use a reference-free SNP calling approach to construct variation sketches, eliminating the need for predefined variant sites. Currently, two main methods are available for reference-free SNP calling. In hybrid approaches, raw reads are assembled into long contigs or scaffolds, and SNPs are then identified by aligning the raw reads to these assembled contigs and mapping them to specific positions [14, 15, 16]. The accuracy of this method depends heavily on assembly quality, and the assembly step itself is time-consuming [17]. The second approach processes data directly based on k-mer counting. For example, DiscoSnp++ [17, 18] constructs a de Bruijn graph from raw data and detects specific patterns to call SNPs. ebwt2snp [19, 20] uses the extended Burrows-Wheeler Transform (eBWT) from reads to identify SNPs as pairs of k-mers. Kmer2SNP [21] simplifies the heterozygous SNP calling problem by finding the maximum weight matching in the heterozygous k-mer graph. It selects k-mers from heterozygous regions based on the k-mer frequency distribution rather than using all k-mers, considerably reducing the amount of data to be processed and improving efficiency. However, the precision and speed of SNP calling in the existing Kmer2SNP algorithm remain limiting factors for downstream analyses, prompting us to make several improvements.

In this work, we developed a reference-free tool for detecting sample swaps that is adaptable to multiple species. The tool eliminates the need for reference information by using a reference-free SNP calling approach to construct variant sketches. Additionally, we improve the SNP calling method that is an order of magnitude faster and enable the detection of sample swaps using only heterozygous SNP information by refining the calculation of inter-sample relationships. Our process requires no additional reference information or downstream steps, such as alignment, making it an efficient QC tool for the upstream stage.

Methods

Algorithm overview

We developed PISAD, a tool designed to detect anomalies in cohort samples without requiring reference information. The tool operates in two primary stages. In stage 1, we performed reference-free SNP calling to construct a variant sketch using low-error-rate data from the target individual. In stage 2, we compared the k-mer counts of other cohort samples on the variant sketch to infer relationships between them (Fig. 1).

SNP calling

In SNP calling, the first step is to select heterozygous k-mers to construct the vertex set. Kmer2SNP uses DSK[22] to count k-mer frequencies from raw reads and generate a corresponding k-mer histogram file. Then, FindGse[23] is employed to identify the frequency range of heterozygous k-mers. However, existing genome analysis methods like FindGse are typically suited only for high-

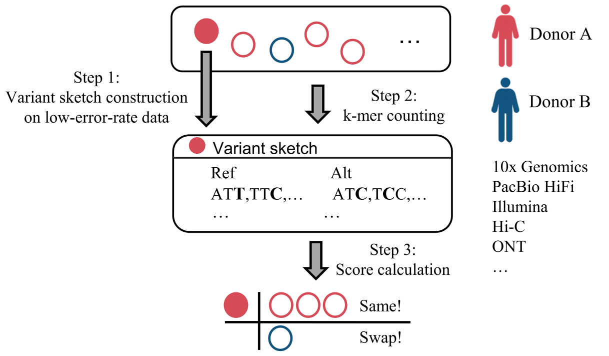

Figure 1. A schematic overview of PISAD. Red and blue circles represent sequencing runs from different donors with heterogeneous data types. The solid circle indicates the data used for variant sketch construction, which, in practice, can be any low-error-rate data (e.g., 10x Genomics, PacBio HiFi, etc.). The mix of blue and red circles represents sample swaps, indicating that samples are incorrectly assigned to the wrong donor.

coverage data around 30x.

To address this limitation, we designed an algorithm to detect heterozygous regions at lower coverage. The algorithm begins by reading the first 1000 points of the k-mer histogram and then checks for a sequence of three consecutive points that shows an upward trend followed by a downward trend. If such a sequence is found, it is defined as a peak. To filter out noise from small peaks, the algorithm will terminate early if either 95% of the total k-mer frequency has been read or if two peaks have already been identified. When two peaks are found, the first peak is assumed to represent the heterozygous peak. If only one peak is identified due to low coverage, an additional parameter is required to indicate whether the heterozygosity rate of the species is greater than 1.2%. This distinction is necessary because, with only one peak, the algorithm cannot reliably determine whether it represents a homozygous or heterozygous region. According to GenomeScope[24], when the heterozygosity rate exceeds about 1.2%, the frequency of the heterozygous peak begins to surpass that of the homozygous peak. Once the heterozygous peak value is identified, the heterozygous region is calculated as follows:

$$\begin{cases} (\lfloor 0.5value \rfloor, \lfloor 1.5value \rfloor) & value > 2, \\ (\lfloor 0.5value + 1 \rfloor, \lfloor 1.5value \rfloor) & value = 2, \\ (1, 2) & value = 1. \end{cases} \quad (1)$$

When the value of the heterozygous peak is well-separated from the error peak, a range of 0.5 to 1.5 times the heterozygous peak value is selected. If the heterozygous peak is closer to the error peak, the left boundary is adjusted to maintain some distance from the error peak. In cases where the heterozygous and error peaks overlap, a portion of the error peak must be included to ensure that a sufficient number of k-mers are selected.

After obtaining the k-mer data for the heterozygous regions, the next step is to call SNPs in these regions. Although Kmer2SNP has demonstrated the best performance among reference-free SNP calling tools, we observed that for whole-genome SNP calling, its runtime often exceeds one hour, and the precision of SNP calling drops sharply as coverage decreases, which is unacceptable for our requirements. Excessive SNP calling time considerably increases the operational cost of using the tool, while a high number of incorrect SNP calls can severely impact our tool's performance. To address these issues, we restructured and optimized the algorithm in C++, incorporating parallel-hash [25] for extensive parallel computation. To further enhance SNP calling precision, we selected

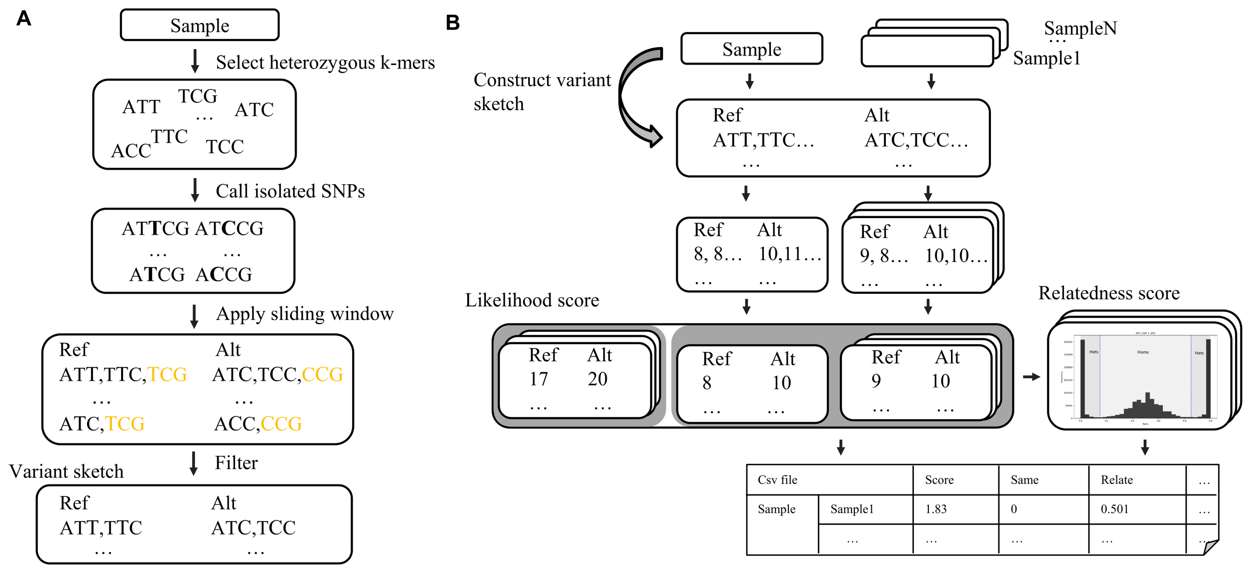

Figure 2. Illustration of key steps. (A) Stage 1: Calling isolated SNPs on low-error-rate data to construct a variant sketch. (B) Stage 2: Using k-mer counting based on the variant sketch to determine the relationship between samples. The input includes the low-error-rate data used for sketch construction and other samples to be tested. The output is a CSV file listing relationships between samples.

only SNPs supported by a length of 21. Our improved Kmer2SNP algorithm only calls isolated SNPs, as these SNPs are independent in subsequent analyses. Since no reference genome is available, all called SNPs are heterozygous.

Sketch construction

After SNP calling, a variant sketch FASTA file is constructed (Fig. 2A). Each called SNP is split into Ref and Alt columns and processed separately using a 21-mer sliding window. Each k-mer is hashed, and identical or reverse-complement k-mers are removed to ensure the independence. When validating relationships between multiple individuals in large cohorts, the process can be repeated for each individual, and the resulting sketches can be merged into a comprehensive sketch for analysis.

Variant k-mer counting

After obtaining and reading the variant sketch FASTA file, we hash it into a hash table using a reversible hash function. Input sequences in FASTQ format are decomposed into k-mers, which are subsequently hashed. Whenever a k-mer matches an entry in the hash table, its read count is incremented by one. These read counts are then used to compute the final score (Fig. 2B). Optionally, the process can be terminated early by specifying an expected coverage threshold to optimize runtime.

Calculating Score

We employed two methods to calculate the relatedness coefficient: relatedness score and likelihood score.The relatedness score provides a detailed measurement of the relationship between samples at higher coverage, while the likelihood score is designed to robustly verify whether two samples are identical under low coverage conditions, primarily to detect sample swaps.

To calculate the relatedness score, allele counts at each site for each sample are first converted into genotypes. Each site includes multiple counts for reference (Ref) and alternative (Alt) alleles. The most frequent count is selected as the genotype for each site to obtain a reliable estimate. Next, the Ref/Alt ratio is calculated, then plotting a histogram to identify the cut-off point at the lowest frequency in the histogram. This cut-off is subsequently used to classify sites as heterozygous or homozygous (Fig. 2B).

After determining the genotype for each site, we calculate the relatedness score based on the differences in observed genotypes between each pair of samples. Existing methods, such as KING[13], rely on the IBS0 statistic, which represents the number of loci where a pair of individuals share zero alleles. For related individuals, such as parent-offspring or siblings, their IBS0 should never be zero unless Mendelian inheritance is violated. However, unlike typical scenarios, our variant sketch includes only heterozygous SNPs from each sample, with no information on homozygous SNPs. Therefore, we define the relatedness score calculation as follows:

$$\frac{Het_i}{Het_i + Hom_i} \tag{2}$$

Here, i represents the sample to be tested, while Het_i and Hom_i are the counts of heterozygous and homozygous sites for sample i, respectively. Sample j is the reference sample used to create the variant sketch. We only observe the counts of sample i, as sample j originates from the SNP calling of the target individual and consists entirely of heterozygous sites. In this context, if two samples are identical, sample i should share all heterozygous SNPs with sample j, and thus the relatedness score is equal to 1. If there is a parent-offspring or sibling relationship between the samples, they should share half of the heterozygous SNPs, resulting in a relatedness score of 0.5. Due to the absence of homozygous SNP information, our tool is currently unable to detect more distant relationships.

Under low data coverage, distinguishing between heterozygous and homozygous genotypes becomes challenging. Therefore, we largely borrow from the exact method described in the ntsm publication. This method employs maximum likelihood estimation[26] to determine whether two samples are identical. It assumes two models: one in which the samples are independent and another in which they are same. A multinomial-like likelihood function is used, and the log-likelihood ratio between the two models is calculated to provide a robust assessment of sample identity (Fig. 2B). We modified the calculation of the final score in ntsm by applying different weights to the coverage of the two samples. This adjustment

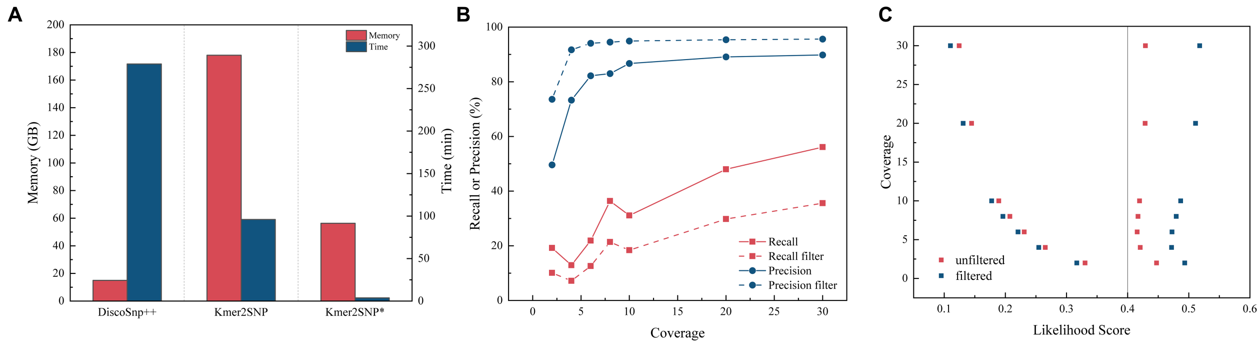

Figure 3. Impact of data coverage. (A) The performance of reference-free SNP calling tools. Each tool only calls isolated SNPs, with other settings following default parameters. DiscoSnp++ and Kmer2SNP* use 8 cores, while Kmer2SNP only supports 1 core. Kmer2SNP* only involves optimizations for time and memory consumption, and its SNP calling results remain consistent with Kmer2SNP. The time statistics do not include the k-mer counting step(DSK). (B) The results of SNP calling performance after filtering by selecting SNPs supported by a length of 21. (C) The results of detecting sample swaps on filtered and unfiltered sketches. The vertical line in the figure represents the threshold for determining whether samples are identical. To achieve better discrimination, the two categories of samples should be as far from the line as possible.

was made because the sample coverage from the target individual has a smaller impact on the results (see the Results section). Our modified formula for the likelihood score is:

$$\frac{\lambda_{LR}}{N \cdot {C_1}^{0.3}{C_2}^{0.2}} \quad (3)$$

Here, λ_{LR} represents the log-likelihood result, N represents the number of sites, and C_1 and C_2 represent the coverage of two samples being compared: specifically, C_1 is the low-error-rate sample from the target individual, and C_2 is each of the other samples being tested. Additionally, we exclude sites where the sum of ref and alt counts was less than 2 to minimize the impact of missing data due to low coverage.

Results

SNP calling

We evaluated the performance of the improved Kmer2SNP using PacBio High-Fidelity (HiFi) sequencing data from the HG002 sample at a depth of 30x. As shown in Fig. 3A, our tool achieves considerably faster SNP calling, requiring only 3.8 minutes and 53.6 GB of memory. Compared to the original Kmer2SNP algorithm, this achieves a 25.2-fold increase in speed and a 3.3-fold reduction in memory consumption. Although DiscoSnp++ benefits from using a Bloom filter, which reduces its memory usage to around 15 GB, its runtime of nearly 4.65 hours makes our tool a more efficient choice.

Subsequently, we analyzed the selection of k-mer sizes from two perspectives. For SNP calling performance, increasing k enhanced overall performance, though it also raised memory usage and runtime. Starting at 21-mer, it achieved high-quality results, which then showed gradual improvement as k increased further (Table 1). In terms of tolerance to high-error data in stage 2, smaller k-mers provide greater redundancy to compensate for sequence errors (Fig. 2B). To balance these factors, we chose a 21-mer, which achieves sufficient SNP calls, shorter runtime, and enhanced performance with high-error-rate data.

Finally, we evaluated SNP calling results across different coverages by using seqkit[27] to subsample HG002 PacBio HiFi sequencing data to the depth(x) of 2, 4, 6, 8, 10, 20, and 30. Fig. 3B shows that both recall and precision improve as coverage increases. However, we found that the precision of SNP calling was insufficient, with a large number of erroneous calls occurring at low coverage levels, especially below 10x. After filtering by selecting only SNPs supported by a length of 21, precision remained above 90% for coverages of 4x and higher, at the cost of some recall. To evaluate the

Table 1. The performance of SNP calling under different k-mer sizes

k-mer	Precision(%)	Recall(%)	Memory(GB)	Time(s)
17	89.1	3.5	23	100
19	90.3	35.1	41	168
21	89.8	56.1	41	178
23	89.6	60.7	42	195
27	89.7	63.9	43	211
31	89.8	65.9	78	290

impact of increased precision and reduced recall on SNP calling results after filtering, we conducted experiments using 4x coverage SNP calls and ONT data from HG002 and HG003. We chose ONT data due to its high error rate, anticipating that it would present the greatest analytical challenge. As shown in Fig. 3C, the filtered variant sketch displays marked enhanced discriminatory power compared to the unfiltered version, enabling the tool to make more robust assessments.

Impact of data coverage

To investigate the impact of data coverage on the results, we selected sequencing data from HG002 and HG003, including Illumina, PacBio HiFi, Hi-C, and ONT sequencing technologies. Since our tool involves a two-stage process, where stage 1 performs SNP calling to create a variant sketch on low-error-rate data (Fig. 2A), and stage 2 uses k-mer counts to evaluate other data against the variant sketch (Fig. 2B), We subsampled sequencing data at different fold coverages (from 2× to 30×) at each stage.

In the data coverage utilized for SNP calling, we found that neither the likelihood nor the relatedness score is particularly sensitive to coverage levels in stage 1 (Fig. 4A,B). The only substantial performance drop occurred at 2x coverage. As shown in Fig. 3B, 2x coverage is also the only condition where precision falls below 90%, suggesting that an excess of erroneous SNP calls reduces tool performance. For coverages above 2x, although the number of SNP calls increased, this increase provided minimal benefit to tool performance compared with the importance of maintaining high precision.

In contrast to the minimal impact of stage 1 coverage, we found that data coverage in stage 2 has a considerable effect on the tool's performance. As coverage increases, the tool's ability to measure relationships improves accordingly. For calculating relatedness score to assess the relationship between two samples, we recommend a minimum coverage of 20x to ensure robust estimates (Fig. 4B).

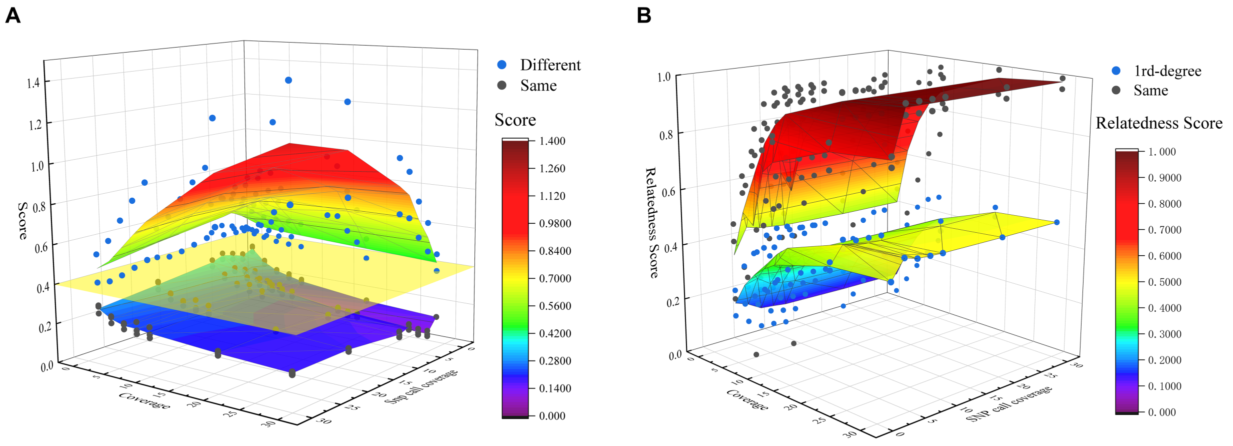

Figure 4. The impact of data coverage. (A) Likelihood score results for different input and SNP calling data coverages. Blue and gray points represent results for the different and same samples. The yellow plane represents the threshold of our method for determining whether samples are identical, set at 0.4. (B) Relatedness score results under the same conditions as (A). Blue and gray points represent results for first-degree relationships and identical relationships, respectively.

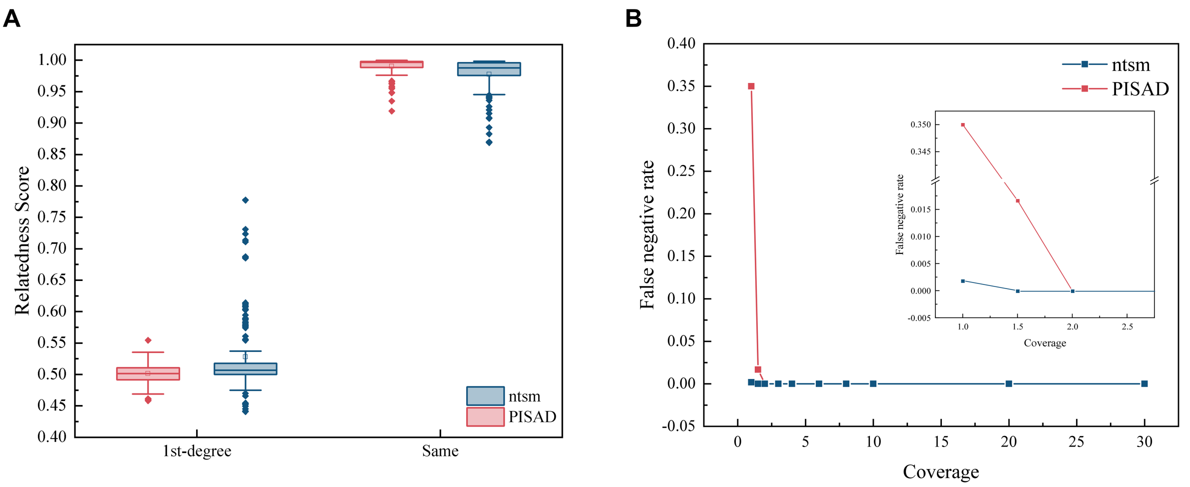

Figure 5. Evaluation results on human samples. (A) Relatedness score results of ntsm and PISAD for 20 human family trio samples. (B) The false negative rate (FNR) of PISAD and ntsm at varying raw dataset coverage. PISAD constructs variant sketches using approximately 15x coverage Illumina data for each sample. ntsm uses the lower coverage between the two samples being compared as the coverage on the x-axis.

However, if the objective is simply to calculate the likelihood score to determine whether the two samples are identical, a much lower coverage level is sufficient (Fig. 4A). We ultimately selected a threshold of 0.4 to determine whether the two samples were identical.

Comparisons to ntsm

To evaluate the performance of our tool, we selected well-studied human samples with extensive and reliable reference information. This choice enabled us to compare our tool with the state-of-the-art reference-based sample swap detection tool, ntsm.

To validate sample swaps, we used 20 samples from the Human Pangenome Reference Consortium (HPRC) [28], which includes sequencing data from Illumina, PacBio HiFi, Hi-C, and ONT platforms. We then subsampled these data to depths of 1x, 1.5x, 2x, 3x, 4x, 6x, 8x, 10x, 20x, and 30x to evaluate the performance of our tool and ntsm under varying coverage levels. Additionally, to calculate relatedness, we supplemented this dataset with samples from the 1000 Genomes Project [29], combining it with HPRC sample to create a dataset of 20 family trios(Supplementary Table S1). Each child in the trios has data from multiple sequencing technologies, while the parents have only Illumina data.

Detecting the sample swap

We evaluated the performance of both tools across different data coverage levels. Even at a low coverage of 1x, we found that the false positive rate (FPR) for both tools remained consistently at 0, maintaining a conservative estimate. At the same time, for the false negative rate (FNR), ntsm misclassified only one identical sample at 1x coverage and achieved stable estimates at 1.5x and above. In contrast, our tool required a minimum coverage of 2x to achieve stable results (Fig. 5B). We attribute this difference primarily to the absence of homozygous variant sites in our method. At shallow coverage, heterozygous sites are more susceptible to misclassification due to insufficient coverage, while homozygous sites provide better discriminative power[30].

Calculating relatedness

For the relatedness score, we found that our tool demonstrated tighter grouping compared to ntsm (Fig. 5A). Further analysis re-

Table 2. Detailed information about other species

Species	Heterozygosity[a]	Sequencing technology	Proband, Sire, Dam	Stage1 coverage[b]	Coverage[c]
Bos taurus	1.12%	Illumina, Hi-C, ONT	22, 2, 2	9.1 - 22.6x	2.4 - 22.6x
Gallus gallus	1.03%	Illumina, Pacbio HiFi, Hi-C, 10x Genomics	23, 7, 10	7.5 - 30.8x	4.9 - 55.4x
Arctia plantaginis	1.90%	Illumina, Pacbio CLR, 10x Genomics	13, 2, 2	9.8 - 24.3x	9.8 - 24.3x

[a] Heterozygosity estimated using Genomescope2.0[31] with at least 30x data.

[b] The coverage range for all sequencing data used to call SNPs and construct sketches. Coverage calculated as total base pairs divided by genome size.

[c] The coverage range for all data to be detected.

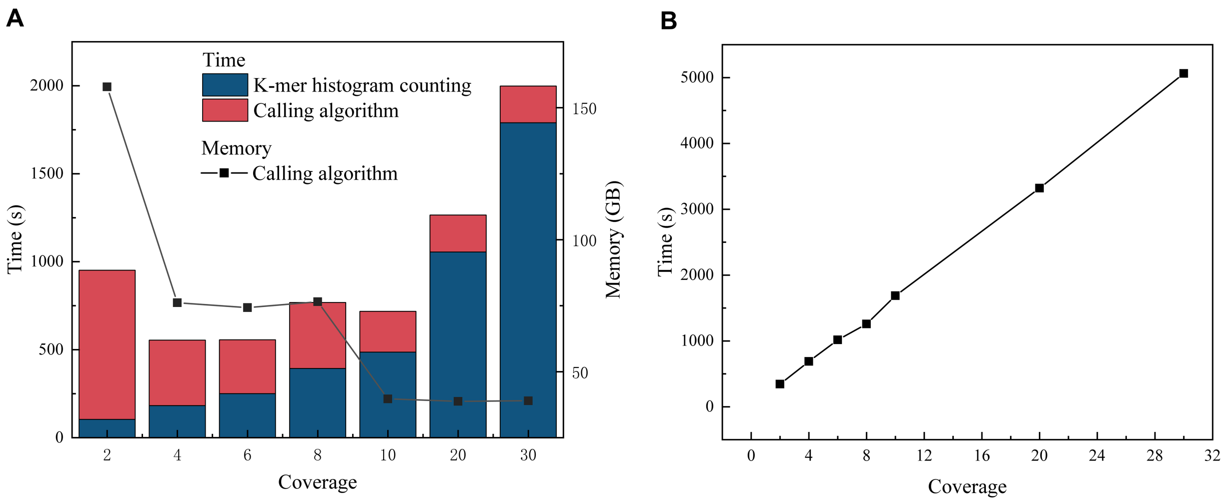

Figure 6. The time and memory consumption of the PISAD. Experiments are conducted on a platform with an Intel(R) Xeon(R) Gold 6348 CPU @ 2.60GHz. (A) The memory and time consumption for reference-free SNP calling with different sequencing depths in stage 1 (using 8 core). The memory consumption during the k-mer histogram counting phase depends on the maximum memory setting configured for DSK, which is set to 30 GB here. (B) The time consumption for k-mer counting on the sketch with different sequencing depths in stage 2 (using 1 core). The memory consumption is independent of the coverage and mainly depends on the size of the sketch, which is approximately 0.5GB in this case.

vealed that most of the poorly estimated results in ntsm were derived from ONT sequencing, likely due to its high error rates. Our approach appears to mitigate the impact of high-error data by consolidating multiple counts at each site into a single value using the mode rather than the maximum, and by leveraging the ref/alt frequency distribution to classify genotypes. However, due to the absence of homozygous SNP information in our method, it lacks the capability to distinguish relationships beyond 1st-degree (parent-offspring and siblings), unlike reference-based tools.

Extension to other species

To assess the performance of our tool on other species in practical application scenarios, we selected family trio samples from three species: *Bos taurus*, *Gallus gallus*, and *Arctia plantaginis*. The first two species were obtained from the Vertebrate Genome Project (VGP)[32], while the third was obtained from a study that performed de novo assembly of *Arctia plantaginis* through trio binning[1]. Details of these datasets are provided in Table 2.

For each species, we first created sketches on all low-error-rate runs. We then validated all data runs against each sketch to evaluate the performance of our tool in real-world scenarios. As shown in Fig. 7, for species with different heterozygosity, such as *Bos taurus*, *Gallus gallus*, and *Arctia plantaginis*, our tool is able to identify all identical and non-identical samples correctly. Additionally, as coverage increased, the results became more confident.

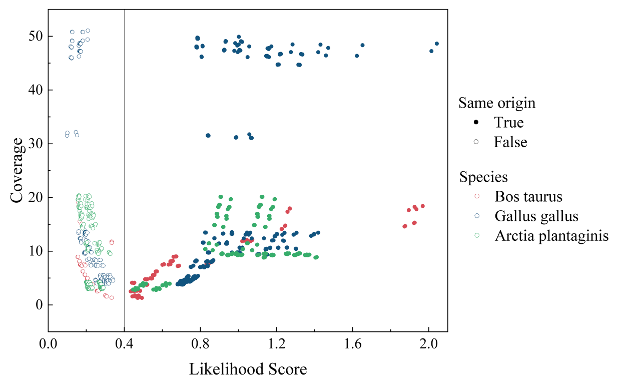

Figure 7. Results of determining whether samples are identical in trio data across different species. The vertical axis represents the coverage of the sample with the lower coverage among the two input samples. The gray vertical line indicates the threshold for determining whether samples are identical.

Running time

Since a large number of files may need to be checked, the speed of the algorithm is crucial. In stage 1, the time consumption mainly stems from two processes: (1) k-mer histogram counting, which uses the DSK algorithm to extract k-mers located in heterozygous regions, and (2) calling algorithm, which performs SNP calling based on these filtered k-mers. The time required for k-mer his-

togram counting increases with the amount of data (Fig. 6A) .For the calling algorithm, memory and time consumption largely depend on the number of heterozygous-region k-mers identified in the k-mer histogram counting phase. At lower data depths, the boundaries of heterozygous regions are less well-defined, which introduces a large amount of irrelevant data and increases both time and memory consumption. However, this improves with deeper coverage. In stage 2, we observed that the runtime increases almost linearly with the depth of data (Fig. 6B).

For a typical run of low-error-rate sequencing data, the coverage is approximately 10x. Under these conditions, the total time for stage 1 is roughly 10 minutes. Additionally, our performance evaluation shows that comparable results can be achieved with coverage as low as 2x. In this case, the computation time for a single sample in stage 2 is approximately 6 minutes. Considering that calculations in stage 2 can be performed in parallel for each sample, a test run on a pair of WGS FASTQ files takes only about 16 minutes.

Disscussion

Comparisons to reference-free tools

Although our tool, like generic k-mer comparison methods such as Mash, does not require reference information, there are significant differences in the workflow. Mash operates by extracting a subset of sequences with the some smallest hash values from the data for comparison. In contrast, our method essentially extracts SNP information from the data for comparison. This difference leads to two key distinctions. First, thanks to the use of SNP information, our method can identify intraspecies sample swaps, whereas Mash is generally limited to distinguishing samples between distinct species. However, our approach is also constrained by the requirements of SNP extraction, which necessitates low-error-rate data with sufficient coverage, even if we can avoid performing SNP calling for each sample by creating sketches. On the other hand, Mash has almost no requirements on the data, making it compatible with various data types. Additionally, in terms of computation, extracting a subset of sequences and performing statistics on them is simpler than performing SNP calling, indexing, and comparing sequences between samples.

Comparsions to reference-base tools

The primary difference between our tool and reference-based methods like ntsm is that we replace the need for pre-defined variant sites with a step of reference-free SNP calling and variant sketch construction, allowing our tool to be applied to multiple diploid species rather than being limited to human. However, based on our performance evaluations, our tool requires slightly higher coverage compared to ntsm due to the lack of homozygous SNP information and reference-based prior knowledge. For species with comprehensive and complete reference information, we still recommend using reference-based tools like ntsm, as they incorporate prior knowledge and perform better on shallow sequencing data. However, for species that lack complete reference information, particularly population-level allele frequency information, such as newly assembled diploid species in the VGP project, our method is a superior choice.

In terms of time consumption, our method generally performs similarly to ntsm. The main difference is the additional step in stage 1, where SNP calling and sketch construction take place, which can be completed in about 10 minutes. Additionally, unlike stage 2, where k-mer counting is required for each sample, to validate a batch of samples and check if they belong to the same individual, we only need one low-error-rate data from the target individual for SNP calling and variant sketch construction.

Current limitations

In stage 1 of our tool, although we have optimized the reference-free SNP calling algorithm to better accommodate low-coverage data and achieve faster SNP calling, its memory consumption, which often exceeds 40 GB, remains challenging for small-memory servers. Additionally, our SNP calling method is still only suitable for low-error-rate data. Since our approach relies solely on k-mers rather than alignment to a reference genome, it has difficulty distinguishing between base errors and true base variants. This limitation means that at least one low-error-rate data from the target individual is required to use our tool effectively. Regarding the scope of application, our method primarily focuses on detecting sample swaps in WGS data and has not yet been tested on other data types such as whole-exome data[33], RNA sequencing[34], or ChIP sequencing[35]. While we are optimistic about the principle behind it (i.e., the use of SNP information), further validation is needed due to differences in sequencing regions and coverage across these data types.

Conclusion

We have developed PISAD to detect intraspecies sample swaps in heterogeneous data cohorts without reference information and have demonstrated its effectiveness in multiple diploid species. It achieves excellent performance even for datasets with sequencing depths as low as 2x and with multiple sequencing technologies. We believe that our tool, which neither requires additional reference information nor downstream analyses like alignments, can be easily integrated into upstream data production pipelines as an efficient QC process.

Availability of source code and requirements

Lists the following:

- Project name: PISAD
- Project home page: `https://github.com/ZhantianXu/PISAD`
- Operating system(s): linux
- Programming language: C++, Python
- Other requirements: TBD
- License: MIT

Data availability

The Illumina, PacBio HiFi, Hi-C, and ONT sequencing data for 20 human family trios are available at Amason S3 under the HPRC/ and HPRC_PLUS/ directories[36]. Details of the selected samples are shown in Supplementary Table S1.

For the coverage experiments, the Illumina, PacBio HiFi, and ONT sequencing data for HG002 and HG003 are available at the National Center for Biotechnology Information (NCBI)[37]. The Hi-C data for HG002 can be accessed at at Amason S3[38].

The data for species *Bos taurus*, *Gallus gallus*, and *Arctia plantaginis* are available from NCBI under the following project accession numbers: PRJNA677946, PRJNA1149711, PRJNA1150343, and PRJEB36595.

Declarations

List of abbreviations

WGS: whole-genome sequencing studies; SNP: single nucleotide polymorphism; eBWT:extended Burrows-Wheeler Transform; Ref: reference alleles; Alt: alternative alleles; bp: base pair; HPRC: Human Pangenome Reference Consortium; MLE: maximum likeli-

hood estimation; ONT: Oxford Nanopore Technology; FPR: false positive rate; FNR: false negative rate; QC: quality control; tsv: tab-separated values; VCF: variant call format;VGP: Vertebrate Genome Project.

Consent for publication

Not applicable

Competing Interests

The authors declare they have no competing interests.

Funding

This work is supported by the National Natural Science Foundation of China (No. 62332020).

Author's Contributions

J.X.W., F.N. and Z.T.X conceived and designed this project. Z.T.X implemented PISAD. Z.T.X and F.N. performed the evaluations and bioinformatics analysis. Z.T.X drafted the manuscript, J.X.W and F.N. contributed to proofreading. All authors read and approved the final manuscript.

Disclosure of use of AI-assisted tools including generative AI

The authors declare that AI tools (ChatGPT - 4o) were used solely for language refinement, and the manuscript has been reviewed by all authors to ensure the accuracy of its content[39].

Acknowledgments

We are grateful for resources from the High-Performance Computing Center of Central South University. The authors thank the anonymous reviewers for their valuable suggestions.

References

1. Yen EC, McCarthy SA, Galarza JA, Generalovic TN, Pelan S, Nguyen P, et al. A haplotype-resolved, de novo genome assembly for the wood tiger moth (Arctia plantaginis) through trio binning. GigaScience 2020;9(8):giaa088.
2. Cheng H, Concepcion GT, Feng X, Zhang H, Li H. Haplotype-resolved de novo assembly using phased assembly graphs with hifiasm. Nature methods 2021;18(2):170–175.
3. Kronenberg ZN, Rhie A, Koren S, Concepcion GT, Peluso P, Munson KM, et al. Extended haplotype-phasing of long-read de novo genome assemblies using Hi-C. Nature Communications 2021;12(1):1935.
4. Ondov BD, Treangen TJ, Melsted P, Mallonee AB, Bergman NH, Koren S, et al. Mash: fast genome and metagenome distance estimation using MinHash. Genome biology 2016;17:1–14.
5. Bergmann EA, Chen BJ, Arora K, Vacic V, Zody MC. Conpair: concordance and contamination estimator for matched tumor–normal pairs. Bioinformatics 2016;32(20):3196–3198.
6. Pedersen BS, Quinlan AR. Who's who? Detecting and resolving sample anomalies in human DNA sequencing studies with peddy. The American Journal of Human Genetics 2017;100(3):406–413.
7. Schröder J, Corbin V, Papenfuss AT. HYSYS: have you swapped your samples? Bioinformatics 2017;33(4):596–598.
8. Javed N, Farjoun Y, Fennell TJ, Epstein CB, Bernstein BE, Shoresh N. Detecting sample swaps in diverse NGS data types using linkage disequilibrium. Nature Communications 2020;11(1):3697.
9. Lee S, Lee S, Ouellette S, Park WY, Lee EA, Park PJ. NGSCheckMate: software for validating sample identity in next-generation sequencing studies within and across data types. Nucleic acids research 2017;45(11):e103–e103.
10. Pedersen BS, Bhetariya PJ, Brown J, Kravitz SN, Marth G, Jensen RL, et al. Somalier: rapid relatedness estimation for cancer and germline studies using efficient genome sketches. Genome medicine 2020;12:1–9.
11. Chu J, Rong J, Feng X, Li H. ntsm: an alignment-free, ultra-low-coverage, sequencing technology agnostic, intraspecies sample comparison tool for sample swap detection. GigaScience 2024;13:giae024.
12. Wang PP, Parker WT, Branford S, Schreiber AW. BAM-matcher: a tool for rapid NGS sample matching. Bioinformatics 2016;32(17):2699–2701.
13. Manichaikul A, Mychaleckyj JC, Rich SS, Daly K, Sale M, Chen WM. Robust relationship inference in genome-wide association studies. Bioinformatics 2010;26(22):2867–2873.
14. Bankevich A, Nurk S, Antipov D, Gurevich AA, Dvorkin M, Kulikov AS, et al. SPAdes: a new genome assembly algorithm and its applications to single-cell sequencing. Journal of computational biology 2012;19(5):455–477.
15. Li H, Durbin R. Fast and accurate short read alignment with Burrows–Wheeler transform. bioinformatics 2009;25(14):1754–1760.
16. Van der Auwera GA, Carneiro MO, Hartl C, Poplin R, Del Angel G, Levy-Moonshine A, et al. From FastQ data to high-confidence variant calls: the genome analysis toolkit best practices pipeline. Current protocols in bioinformatics 2013;43(1):11–10.
17. Uricaru R, Rizk G, Lacroix V, Quillery E, Plantard O, Chikhi R, et al. Reference-free detection of isolated SNPs. Nucleic acids research 2015;43(2):e11–e11.
18. Peterlongo P, Riou C, Drezen E, Lemaitre C. DiscoSnp++: de novo detection of small variants from raw unassembled read set (s). BioRxiv 2017;p. 209965.
19. Prezza N, Pisanti N, Sciortino M, Rosone G. SNPs detection by eBWT positional clustering. Algorithms for Molecular Biology 2019;14:1–13.
20. Prezza N, Pisanti N, Sciortino M, Rosone G. Variable-order reference-free variant discovery with the Burrows-Wheeler Transform. BMC bioinformatics 2020;21:1–20.
21. Li Y, Patel H, Lin Y. Kmer2SNP: reference-free SNP calling from raw reads based on matching. In: 2020 IEEE International Conference on Bioinformatics and Biomedicine (BIBM) IEEE; 2020. p. 208–212.
22. Rizk G, Lavenier D, Chikhi R. DSK: k-mer counting with very low memory usage. Bioinformatics 2013;29(5):652–653.
23. Sun H, Ding J, Piednoël M, Schneeberger K. findGSE: estimating genome size variation within human and Arabidopsis using k-mer frequencies. Bioinformatics 2018;34(4):550–557.
24. Vurture GW, Sedlazeck FJ, Nattestad M, Underwood CJ, Fang H, Gurtowski J, et al. GenomeScope: fast reference-free genome profiling from short reads. Bioinformatics 2017;33(14):2202–2204.
25. Popovitch G, A family of header-only, very fast and memory-friendly hashmap and btree containers.p;. Accessed: 13/11/2024. https://github.com/greg7mdp/parallel-hashmap.
26. Fisher RA. On the mathematical foundations of theoretical statistics. Philosophical transactions of the Royal Society of London Series A, containing papers of a mathematical or physical character 1922;222(594-604):309–368.

27. Shen W, Le S, Li Y, Hu F. SeqKit: a cross-platform and ultrafast toolkit for FASTA/Q file manipulation. PloS one 2016;11(10):e0163962.
28. Liao WW, Asri M, Ebler J, Doerr D, Haukness M, Hickey G, et al. A draft human pangenome reference. Nature 2023;617(7960):312–324.
29. Consortium GP, Auton A, Brooks L, Durbin R, Garrison E, Kang H. A global reference for human genetic variation. Nature 2015;526(7571):68–74.
30. Hemstrom W, Grummer JA, Luikart G, Christie MR. Next-generation data filtering in the genomics era. Nature Reviews Genetics 2024;p. 1–18.
31. Ranallo-Benavidez TR, Jaron KS, Schatz MC. GenomeScope 2.0 and Smudgeplot for reference-free profiling of polyploid genomes. Nature communications 2020;11(1):1432.
32. Rhie A, McCarthy SA, Fedrigo O, Damas J, Formenti G, Koren S, et al. Towards complete and error-free genome assemblies of all vertebrate species. Nature 2021;592(7856):737–746.
33. Albert TJ, Molla MN, Muzny DM, Nazareth L, Wheeler D, Song X, et al. Direct selection of human genomic loci by microarray hybridization. Nature methods 2007;4(11):903–905.
34. Wang Z, Gerstein M, Snyder M. RNA-Seq: a revolutionary tool for transcriptomics. Nature reviews genetics 2009;10(1):57–63.
35. Johnson DS, Mortazavi A, Myers RM, Wold B. Genome-wide mapping of in vivo protein-DNA interactions. Science 2007;316(5830):1497–1502.
36. Human pangenomics stored in Amazon Simple Storage Service (Amazon S3);. Accessed: 13/11/2024. https://s3-us-west-2.amazonaws.com/human-pangenomics/index.html?prefix=working.
37. Ashkenazim trio data stored in the National Center for Biotechnology Information (NCBI);. Accessed: 13/11/2024. https://ftp-trace.ncbi.nlm.nih.gov/ReferenceSamples/giab/data/AshkenazimTrio.
38. The HG002 Hi-C sequencing data stored in Amazon Simple Storage Service (Amazon S3);. Accessed: 13/11/2024. https://s3-us-west-2.amazonaws.com/human-pangenomics/index.html?prefix=NHGRI_UCSC_panel/HG002/hpp_HG002_NA24385_son_v1/hic/downsampled.
39. OpenAI(2024), ChatGPT (GPT-4o, November 13 Version) [Large language model]. Polish the sentence;. Accessed: 13/11/2024. https://chat.openai.com/chat.

Click here to access/download
Supplementary Material
TableS1_Supplementary Material.pdf
