# Supplementary material for: PISAD: reference-free intraspecies sample anomalies detection tool based on k-mer counting
Source: Gigascience. 2025 Jun 17;14:giaf061. doi: 10.1093/gigascience/giaf061 (PMC12202988; doi:10.1093/gigascience/giaf061)
Supplement: giaf061_GIGA-D-24-00517_Revision_1 [file giaf061_giga-d-24-00517_revision_1.pdf]

GigaScience

PISAD: reference-free intraspecies sample anomalies detection tool based on k-mer counting

--Manuscript Draft--

**Manuscript Number:**	GIGA-D-24-00517R1
**Full Title:**	PISAD: reference-free intraspecies sample anomalies detection tool based on k-mer counting
**Article Type:**	Research
**Funding Information:**	National Natural Science Foundation of China (62332020) — Prof. Jianxin Wang
**Abstract:**	Background: Genomic sequencing research often requires the simultaneous analysis of heterogeneous data types across single or multiple individuals, introducing a substantial risk of sample swaps (e.g., labeling errors). Existingmethods primarily rely on reference information, requiring the pre-selection of informative variant sites with a population allele frequency around 0.5, which may be insufficient or unavailable for non-model organisms. As research expands to encompass a growing number of new species, a robust quality control tool will become increasingly important.<br>Finds: We developed PISAD, a tool for validating sample identities in whole-genome sequencing (WGS) data without requiring reference information. It uses a two-stage approach: first, it performs rapid, reference-free SNP calling on low-error-rate data fromthe target individual to create a variant sketch; then, it assesses the concordance of other samples on this sketch to verify relationships. We assessed the performance and efficiency of PISAD on Homo sapiens, Bos taurus, Gallus gallus, Arctia plantaginis and Pyrus species.<br>Conclusion: Our evaluation showed that PISAD achieves a lower data coverage requirement (0.5×) compared to the reference-based tool ntsmand is broadly applicable tomultiple diploid species.
**Corresponding Author:**	Jianxin Wang<br>Central South University<br>Changsha, -Select- CHINA
**Corresponding Author Secondary Information:**	
**Corresponding Author's Institution:**	Central South University
**Corresponding Author's Secondary Institution:**	
**First Author:**	Zhantian Xu
**First Author Secondary Information:**	
**Order of Authors:**	Zhantian Xu
	Fan Nie
	Jianxin Wang
**Order of Authors Secondary Information:**	
**Response to Reviewers:**	Responses to the Reviewers' Comments<br><br>" PISAD: reference-free intraspecies sample anomalies detection tool based on k-mer counting "<br><br>By Zhantian Xu, Fan Nie, and Jianxin Wang<br><br>Dear Editors ,<br><br>We would like to thank all the reviewers and AE for careful reading, constructive

suggestions, and comments, which have significantly improved the presentation of our paper. We have revised the manuscript accordingly. The manuscript has also been double-checked, and the typos and grammar errors we found have been corrected. We hope that our revised manuscript is satisfactory.

In the following, we give our point-wise response. We copy the original review comments and mark them in italics and blue. Our responses are given in roman font. The changes in the manuscript are highlighted with red color.

All authors have read and approved the final version of the manuscript.

Thanks very much for your consideration!

Professor Jianxin Wang
School of Computer Science and Engineering,
Central South University
Changsha, Hunan, P.R. China
Email: jxwang@mail.csu.edu.cn

Answers to Reviewer #1

Comments:
"In PISAD, Xu et al. created a new software and algorithm for quickly detecting the similarity of two high-throughput sequencing samples for similar organisms at the sub-species level. This is similar to existing sketching approaches, but their innovation lies in using a reference-free SNP calling technique to avoid using a reference genome. This allows PISAD to work on various diploid organism sequencing projects that do not have good reference genomes -- an important task.
Overall, I found the paper relatively clear, although I was confused at some places. The methodology was well-motivated. The experimentation was thorough and well thought out. I have a few comments below that may improve the paper and its presentation.''
Response:
We greatly appreciate your careful consideration of our manuscript and their positive remarks. Thank you for your valuable comments, which motivate us to improve our work. Next, we will address your comments one by one.

Major Comments:
1."I feel strongly that software for a methodological paper should have a very simple install. This means either through (1) a package manager (conda), or (2) as a static binary (least preferable), or (3) have minimal dependencies for compiling. PISAD does not appear to be available in conda (the authors mention "recommend use conda to install", but I don't see PISAD on bioconda). Furthermore, it has non-trivial dependencies (hdf5, boost, DSK) that are not readily available on average linux machines. Please consider adding at least a self-contained bioconda install.
Some other notes:
- I think practitioners prefer dashed commands (--) versus positional commands. The commandline interface is a bit cumbersome
- Based on the GitHub README, can variant calling be multithreaded?''
Response:
We sincerely appreciate your feedback regarding the installation process. To simplify installation, PISAD is now available on Bioconda and can be installed directly using: Conda(conda install bioconda::pisad).
We have revised the command-line interface to adopt a standardized dashed command format for improved usability. The GitHub README has been comprehensively updated with clearer instructions and more detailed usage examples. Variant calling supports multi-threading, and the README has been refined to explicitly clarify its usage, minimizing potential confusion.

2."The authors confine themselves to diploid animal genomes. I think it would be very interesting if PISAD could work for 1) diploid plants and 2) allopolyploid genomes. Allopolyploids often "contain" two diploid genomes in a sense. Allopolyploid genomes

may be difficult for this algorithm, but many plant geneticists would be interested in at least diploid plants. Effectiveness on diploid plants would allow this paper to reach a wider audience.. ‘’

Response:

We sincerely appreciate your insightful suggestions. To broaden the applicability of PISAD, we have conducted additional experiments on the diploid plant Pyrus and extended the tool’s functionality to diploid plants. We have incorporated the Pyrus results into Figure 5C.

Regarding allopolyploid genomes, SNP calling in polyploids remains a complex challenge, even in reference-based approaches, making reference-free SNP calling even more difficult. As a result, this study primarily focuses on diploid species. However, we recognize the potential of extending PISAD to polyploid genomes and consider this an important direction for future research. We have expanded the discussion in Section Current Limitations to address these aspects more comprehensively.

“Additionally, our current study is exclusively focused on diploid species. Polyploid organisms have not been included in this investigation due to the inherent challenges in SNP calling associated with their complex genomic architectures, which warrant separate and more extensive future research.”

Minor Comments:

1.“I think Cortex (Iqbal et al., Nature Genetics 2012) should be cited in addition to DiscoSNP, since it's one of the first approaches. ‘’

Response:

We appreciate your suggestion and have incorporated a citation to Cortex in Section Introduction as follows:

"For example, Cortex and DiscoSnp++ construct a de Bruijn graph from raw data and detect specific patterns to call SNPs."

2.“The authors could make it more explicit that stage 1 does not work for Nanopore data ("high" error rate) but stage 2 works for Nanopore, perhaps at the end of the "Algorithm overview" section. ‘’

Response:

Thanks for your valuable suggestion. To enhance clarity, we have added the following statement at the end of Section Algorithm Overview:

"It is important to note that Stage 1 of our tool supports only low-error-rate sequencing data, meaning most ONT data (except duplex) cannot be processed. However, Stage 2 is compatible with various sequencing technologies. Therefore, at least one low-error-rate dataset is required for reliable sketch construction."

3.“The algorithm begins by reading the first 1000 points of the k-mer histogram…" what are the first 1000 points? Is the x-axis the k-mer frequency and y-axis is # of kmers? Is 1000 the k-mer count? Please clarify this sentence. ‘’

Response:

Thanks for your valuable suggestion. To prevent misunderstandings, we have revised the sentence in section SNP Calling as follows:

"reading the first 1000 entries from the histogram of k-mer abundances, which records the number of distinct k-mers for each occurrence frequency."

The x-axis and y-axis in Figure 2A represent k-mer occurrence frequency and the number of distinct k-mers, respectively.

4.“Then, FindGse[23] is employed to identify the frequency range of heterozygous k-mer…" the authors then state that they designed their own algorithm to detect heterozygous k-mers. I'm a bit confused about if FindGse is being used at all?”

Aside: perhaps the paper "Skmer: assembly-free and alignment-free sample identification using genome skims" may be of interest for low-coverage k-mer statistics.

Response:

Thanks for your valuable suggestion. We sincerely apologize for the confusion. FindGse was not used in our approach, as our evaluation showed that it is not suitable for low-coverage data. Instead, we developed a heuristic method to extract heterozygous regions from shallow sequencing data. To clarify this, we have revised the description in the SNP Calling section as follows:

"Existing genome analysis tools, such as FindGse, are typically designed for high-

Powered by Editorial Manager® and ProduXion Manager® from Aries Systems Corporation

coverage data (>30×) and are not well-suited for shallow sequencing. Therefore, we developed a heuristic algorithm to estimate the range of heterozygous regions under low-coverage conditions."
We also appreciate your suggestion regarding Skmer. After reviewing this study, along with related works such as GenomeScope, we incorporated curve fitting using two negative binomial distributions to estimate genomic characteristics. Additionally, we visualized these results to assist users in making assessments (Figure 2A).

5.“The terms "SNPs supported by a length of…" and "isolated" SNPs need to be explained more. I believe these are terms used in the Kmer2SNP paper, but they are confusing without context. I'm not sure about the definition of "supported‘’
Response:
Thanks for your valuable suggestion. The phrase "SNPs supported by a length of" refers to the maximum possible expansion distance on both sides of an SNP. "Isolated SNP" refers to SNPs that are separated by at least (k−1)/2 bases, where k is the k-mer length.

To improve clarity, we have added a brief explanation of the complete SNP calling process and relevant terminology in Section SNP Calling. Additionally, Figure 2B has been included to illustrate key components. The revised description states:
"KmerSNP first identifies k-mers within heterozygous regions that differ by only a single nucleotide in the middle and designates them as potential SNPs. It then expands both sides of each potential SNP, where the maximum possible expansion distance is defined as the support length. Finally, the algorithm selects the final SNPs by computing the maximum-weight matching of all potential SNPs."

6.“We chose ONT data due to its high error rate, anticipating that it would present the greatest analytical challenge…" this paragraph appears to be slightly out of place since it's in the "SNP calling" section. But it talks about likelihood scores. ‘’
Response:
We acknowledge the misplaced placement of this paragraph within Section SNP Calling. To improve the logical flow, we have introduced a new section titled "Impact of SNP Site" and relocated the content accordingly.

7.“Can the authors mention the error rates of these ONT reads being used? ONT read error rates is highly variable and it can range from 95-99%, so a specific number is helpful ‘’
Response:
We appreciate your suggestion and have added the specific error rates associated with the ONT reads used in our study. The flow cell version and the accuracy of the ONT data are now specified, and more detailed experimental information has been included in Table 1.

8.“Fig. 4 is interesting but hard to read. Here are some issues:
- The blue and gray colours are not easy to differentiate for me. Using different markers (e.g. a square and circle) would help.
- The axis labels are hard to read, because they are flattened. E.g. "SNP call coverage" is not legible.
- If there's enough space, 2-d projections of Fig. 4 onto Coverage = 30 or SNP call coverage = 30 would help readers a lot and make the author's points more clear, since 3-d can be hard to visualize.‘’
Response:
Thank you for your feedback. We have made the following revisions to improve the clarity of Figure 4:
(1) The blue and gray colors have been replaced with more distinguishable colors (blue and red) to better differentiate the datasets.
(2) We have adjusted the axis labels to ensure they are more legible and added clear explanations of the x and y axes in the figure legend.
(3) A 2D projection of Figure 4A with the SNP call coverage axis at a value of 4 has been added as Figure 4C to assist readers in interpreting the data more easily.

Powered by Editorial Manager® and ProduXion Manager® from Aries Systems Corporation

9.“As a comment, the 0.4 likelihood score seems arbitrary and unsatisfying. I'm not sure if the authors can do anything about this, however. I'm curious if the authors have thought about a theoretical basis for this value. ‘’
Response:
Thank you for your thoughtful comment. We recognize the concern regarding the 0.4 likelihood score, and we have made some modifications to address this.
As shown in Figure 2C, we revised the original sample swap score calculation to eliminate two empirical bias parameters and adjusted the input for the target individual's sketch to a sample with Ref/Alt values both set to 1. As shown in Figure5B, this change reduces the coverage requirement from 1.5x to 0.5x, which is lower than the 1x requirement of NTSM method. Details of this modification can be found in Section Calculation Score.
Additionally, to provide a more solid theoretical foundation, we have incorporated a simulation-based approach. By modeling scenarios with both identical and distinct samples, we derived a theoretical threshold for our method. This threshold of 0.63 was validated experimentally and is shown to perform well in real-world conditions, as demonstrated in Figures 3C and 4A, 4C.

10.“Fig. 7: low likelihood scores indicate different origin. In Fig. 4: low likelihood scores indicate the same origin. Is this an error, or am I misunderstanding? ‘’
Response:
Thank you for pointing this out. You are correct that the labels in Figure 7 in the original manuscript were reversed. In fact, a low likelihood score represents the same origin. To resolve this inconsistency, we have corrected the labels and swapped the x and y axes in this figure to align with the other figures. The revised version is now shown in Figure 5C in the revised manuscript.

11.“Our approach appears to mitigate the impact of high-error data by consolidating multiple counts at each site into a single value using the mode rather than the maximum, and by leveraging the ref/alt frequency distribution to classify genotypes." I didn't quite understand this. Maybe give an example to explain this further. ‘’
Response:
Thank you for your feedback. To make our methodology clearer, we have revised the description in Section Calculating Relatedness as following:
"Our method differs from ntsm in two key aspects. First, when merging multiple counts at each site, we use the mode instead of the maximum value. For example, if we observe counts like 8, 9, 8, 7 at a given site, we take the mode (which would be 8) rather than the maximum (which would be 9). Second, instead of using a fixed threshold to determine the genotype, we determine the threshold by identifying the minimum value of the Ref/(Ref+Alt) spectrum within a certain range. We believe these modifications help mitigate the impact of high error rates, leading to a more stable and compact genotype distribution."

Answers to Reviewer #2

Comments:
“This manuscript presents a new tool, PISAD, for detecting sample swaps in low-error-rate WGS sequencing data. This is an important problem and PISAD uses k-mer counting instead of read alignment for this task, making it potentially faster than other tools and more applicable to non-model species with no public genome assembly.
The associated code repository is well documented, and the code appears to be well written. I appreciated the authors providing a full report of how they used ChatGPT to refine their language. I tried to review it as much as I could and I found one case where chatgpt removed an important detail from the original writing, but the authors appeared to add the detail back into the submitted version of the manuscript. I take this as evidence that the chatgpt output was well scrutinized.
My major comments mostly refer to ensuring that claims in the abstract reflect the evidence in the paper and including more details in the methods. I think it is a little difficult to evaluate some of the claims in the paper without more detail in the methods. I hope my feedback is helpful. I didn't see line numbers, so I did my best to describe where my comments point to’’
Response:

Powered by Editorial Manager® and ProduXion Manager® from Aries Systems Corporation

Thank you for your thoughtful and constructive comments. We appreciate your recognition of our framework, PISAD, and its contributions to sample swaps detection.

Major Comments:

1.“One claim in the abstract is that PISAD works for low-error-rate data, but some other common types of low-error rate data were not tested in the manuscript (e.g. reduced-representation seq, GBS, pool-seq etc.). I think it would help to further clarify in the abstract the types of data PISAD was tested on, because lots of population-level datasets in non-model species (which seem to be the focus for PISAD) use RAD-seq or similar methods, which are technically low-error-rate but are generally low coverage. It is acknowledged in the current limitations section that extending to data types like RAD-seq is a future research direction, but most readers will only look at the abstract.’’

Response:

Thank you for your insightful comment. We apologize for the lack of clarity in the abstract. To address this, we have revised the abstract to specify that PISAD is currently applicable to WGS data only:

"We developed PISAD, a tool for validating sample identities in whole-genome sequencing (WGS) data without requiring reference information."

We also conducted preliminary tests on data types like RAD-seq and found that calling SNPs from such low-coverage data without reference posed greater challenges to precision. Specifically, the k-mer-based method resulted in a higher incidence of false SNPs, with a precision of only 70%–80%. As such, we acknowledge that extending PISAD to RAD-seq and similar low-coverage data types is a valuable direction for future research. This has also been clarified in Section Current Limitations.

"Meanwhile, calling SNPs from such data without reference poses greater challenges to precision compared to WGS data. Due to the absence of designated heterozygous regions, the k-mer-based method resulted in a higher incidence of false SNPs from homologous/repetitive sequences, yielding a precision of only 70%–80%. This will also constrain the tool’s resolution capability, warranting further investigation."

2.“The abstract also says that PISAD performs similarly to reference-based tools (plural), but as far as I can tell only one reference-based tool (ntsm) was investigated. It would be nice if the abstract said what tools were used for comparison.’’

Response:

Thank you for your comment. You are correct that we only compared PISAD with NTSM, as it is the only reference-based tool we are aware of that supports both fastq sequencing data and high-error-rate data such as ONT. To avoid any misunderstanding, we have revised it in Abstract as follows:

"Our evaluation showed that PISAD achieves a lower data coverage requirement (0.5×) compared to the reference-based tool NTSM and is broadly applicable to multiple diploid species."

3.“Under the "SNP calling" section in methods, 2nd paragraph, there is an algorithm to determine a range of coverage values that is likely predominated by k-mers from heterozygous regions and it is claimed that this algorithm helps identify heterozygous k-mer regions in lower coverage datasets. If I understand correctly, the algorithm is to define peaks as a sequence of 3 k-mer frequencies with an upward trend, followed by 3 frequencies with a downward trend and the first such peak is usually taken as the mean of the heterozygous k-mer distribution. I am a little skeptical whether this works because it seems like a small fluctuation in k-mer frequencies due to sampling error could create a false peak. There is also a set of values for calculating the bounds of the heterozygous k-mer peak that seems somewhat arbitrary. Are these bounds configurable by a user? Will PISAD output any type of histogram with the coverage cutoffs marked so that users can confirm that the automaticheterozygous peak identification gives reasonable results? I think it would be helpful to have an example k-mer histogram from a low coverage dataset showing how this algorithm identifies peaks. I am also a little unsure whether it is necessary to only search for k-mers in the heterozygous peak, besides potentially a gain in computational speed. The distribution of heterozygous k-mer frequencies should follow a Poisson or negative binomial distribution from 0 to infinity, so there could be true heterozygous k-mers outside the peak.’’

Response:

Thank you for your detailed comment. We appreciate your concerns regarding the

	algorithm for identifying heterozygous k-mer regions.<br>We first address your concern about the potential presence of false peaks. Our algorithm restricts peak identification to the region where the cumulative k-mer frequency sum remains below the 95% threshold. This design significantly reduces the probability of false positive peaks, as illustrated in Figure 2A. The rationale behind this approach is twofold: First, k-mers within this high-frequency region (constituting 95% of the cumulative spectrum) exhibit robust frequency signals that are inherently resistant to sampling variations. Second, while low-frequency regions (beyond the 95% threshold) may be prone to noise-induced fluctuations, these areas are explicitly excluded from our peak detection framework, thereby enhancing the overall reliability of our results.<br>Next, we sincerely apologize for any confusion caused regarding the selection of heterozygous regions and the reasoning behind it. We have now made comprehensive revisions in the SNP calling section, as detailed below.<br>“Theoretically, heterozygous region distribution adheres to a Poisson or negative binomial distribution ranging from 0 to infinity. However, directly selecting k-mers from these regions 1) greatly increases algorithm runtime and memory consumption and 2) generates numerous false SNPs from chimeric sequences (overlapping k-mers in the SNP sequence actually originating from different reads) and homologous/repetitive sequences. Inspired by the Kmer2SNP concept, we heuristically designate heterozygous peaks of 0.5×–1.5× as heterozygous regions (minimum left boundary of 2 to exclude erroneous k-mers), ensuring the capture of most k-mers in these regions while maximally reducing runtime, memory usage, and false SNPs.”<br>Moreover, to further improve the reliability and usability of this algorithm module, we allow users to specify any parameters and provide histograms with clear annotations of heterozygous regions and other relevant information for user verification. Additionally, drawing inspiration from methods like Genomescope and skmer, we incorporated curve fitting using two negative binomial distributions to estimate genomic characteristics, facilitating further user validation (Figure 2A). For more detailed heterozygous region selection results across various coverage depths, please refer to Supplemental Figure 1.<br>“Finally, we fitted the k-mer frequency distribution using two negative binomial distributions and provided a rough estimate of genomic characteristics to assist users in evaluating the correctness of the algorithm’s selection. The results of heterozygous region selection are shown in Figure 2A and Supplemental Figure 1.”<br><br>4.“I think describing the SNP calling process, once heterozygous k-mers are identified, in more detail would be helpful. It appears the manuscript presents an optimized version of a previous algorithm, kmer2snp, which sounds like a great advance by itself and perhaps warrants mentioning in the abstract. I read about kmer2snp and it seems the algorithm essentially identifies k-mer pairs that differ at their central bp as putative SNPs, but this is not really described in the current manuscript. Providing a brief description of the algorithm would be helpful.’’<br>Response:<br>Thank you for your suggestion. We have added a brief explanation of the complete SNP calling process and relevant terminology in Section SNP Calling as following:<br>“KmerSNP first identifies k-mers within heterozygous regions that differ by only a single nucleotide in the middle, designating them as potential SNPs. It then expands both sides of each potential SNP, where the maximum possible expansion distance is defined as the support length. Finally, the algorithm refines SNP selection by computing the maximum-weight matching of all potential SNPs.”<br>Additionally, Figure 2B has been included to illustrate key components.<br><br>5.“The methods section would benefit from having a section that describes the benchmarking datasets in more detail. Some details of the datasets are included in the "data availability" section and table 2, but I cannot tell just by reading the ID numbers what the underlying biology is of the samples. What tissue are we looking at? Are these cancer samples? How many samples are used for each test?’’<br>Response:<br>Thank you for your suggestion. We have revised the table to include more detailed biological characteristics of the samples, including tissue type, whether the samples are from cancer or non-cancer sources, and the number of samples used in each test. This updated table has now been relocated to Table 1 in the revised manuscript for

Powered by Editorial Manager® and ProduXion Manager® from Aries Systems Corporation

better clarity.

6.“Table 1: What coverage level was used for the comparison in this table? This seems to be a main determinant of precision and recall.’’
Response:
Thank you for your comment. Table 1 in the original manuscript used data from HG002 PacBio HiFi with 30× coverage. This table focuses on illustrating the impact of k-mer length on the results. We have now added the coverage information to the table legend for clarity, and the table has been relocated to Supplementary Table 1.

7.“It is claimed that in Fig. 5A the new tool of the manuscript has lower variance in relatedness score than ntsm (under heading for "calculating relatedness" in results). This claim should be backed by some kind of statistical test. It's most likely significant just from a glance.’’
Response:
Thank you for your valuable suggestion. We have conducted a Levene’s test for variance in the data and updated Figure 5A to include the results after significance testing to confirm the observed differences.

Minor Comments:
1.“Does the name PISAD mean anything? I was expecting this name to be an acronym, but I didn't see one in the paper.”
Response:
Thank you for your observation. We have added an explanation of the meaning of PISAD in the Section Introduction as follows:
"In this work, we developed PISAD, a Phased Intraspecies Sample Anomalies Detection tool, suitable for multiple species without reference information."

2.“I am curious why the likelihood score cutoff of 0.4 appears to work so well. I don't think this question needs to be answered for the tool to be useful (significance score cutoffs are always somewhat arbitrary) but I am curious if the choice of cutoff has something to do with the coverage levels used and the error rates of the datasets?”
Response:
Thank you for your thoughtful comment. We recognize the concern regarding the 0.4 likelihood score, and we have made some modifications to address this.
As shown in Figure 2C, we revised the original sample swap score calculation to eliminate two empirical bias parameters and adjusted the input for the target individual's sketch to a sample with Ref/Alt values both set to 1. As shown in Figure 5B, this change reduces the coverage requirement from 1.5x to 0.5x, which is lower than the 1x requirement of NTSM method. Details of this modification can be found in Section Calculation Score.
Additionally, to provide a more solid theoretical foundation, we have incorporated a simulation-based approach. By modeling scenarios with both identical and distinct samples, we derived a theoretical threshold for our method. This threshold of 0.63 was validated experimentally and is shown to perform well in real-world conditions, as demonstrated in Figures 3C and 4A, 4C.
Regarding threshold selection, our analysis reveals two key observations from Figures 3C and 4C: (1) increasing sequencing coverage enhances the discriminative power between identical and different samples, while (2) higher error rates reduce this differentiation capability. Based on these findings, we established the identification threshold at 0.63, corresponding to the average similarity value between same and different samples under the most challenging condition (0.1× coverage) in our simulations. This conservative approach ensures robust sample discrimination even in low-coverage scenarios. Furthermore, to comprehensively evaluate the impact of sequencing errors, we have incorporated additional analysis in Section Impact of Stage 2 Coverage, specifically examining how error rates influence identification accuracy.
“Meanwhile, we observsed that in sample swap detection, ONT data presented the greatest challenge in distinguishing samples. Its relatively high error rate reduces the number of effective sites, necessitating higher coverage for improved discrimination.”

3.“Low precision seems like it could be an issue, though perhaps it is not. PISAD has a recall/sensitivity of about 50 % even at high coverage (Fig. 3B). In other words, only 50

Powered by Editorial Manager® and ProduXion Manager® from Aries Systems Corporation

	% of the SNPs that are truly present in the sample are being called. At first glance, this seems to be lower than similar reference free SNP calling (see discosnp papers). My expectation is that if enough SNPs can be called to distinguish samples this is not an issue. However, the manuscript mainly examines whole genome data, and I would expect this to be more of a problem for reduced representation datasets (RAD-seq, GBS, exome-seq, RNA-seq), where only a small portion of the genome is sequenced and thus only a small portion of SNPs are sampled."<br>Response:<br>Thank you for your comment. We appreciate the raised concerns about sufficient SNP sites and have introduced corresponding improvements to tackle this challenge.<br>First, we would like to clarify the performance of similar reference-free SNP calling tools in whole-genome sequencing, such as DiscoSnp. We conducted the evaluation under the same conditions as in Figure 3B, with the results presented in Supplementary Figure 2. The comparative analysis reveals that both DiscoSNP and Kmer2SNP demonstrate comparable performance in heterozygous SNP detection, with recall rates plateauing at 50-60% even at high sequencing coverage. Notably, this performance falls substantially below the 80% recall rate reported in the original DiscoSNP study. This discrepancy likely stems from fundamental differences in evaluation frameworks - while previous reports used simplified single-chromosome simulations, our whole-genome assessment more accurately reflects real-world complexity. We hypothesize that in whole-genome scenarios, the presence of cross-chromosomal k-mers interferes with the formation of characteristic symmetrical bubble structures, thereby impairing detection sensitivity for heterozygous variants.<br><br>Secondly, we acknowledge the potential concerns regarding the sufficiency of SNP sites. To address this issue, we have supplemented Section Impact of SNP Sites with a new experiment designed to systematically evaluate the tool's performance stability under varying SNP site quantities through simulation studies (Figure 4D):<br>"Meanwhile, we further investigated the number of SNPs used in the variant sketch in stage 1. From all heterozygous SNPs called at 30× coverage in HG002, we randomly sampled 5k, 10k, 20k, 40k, 60k, 100k, and 200k SNP sites, corresponding to 0.25%, 0.5%, 1%, 2%, 3%, 5%, and 10% of the total heterozygous SNPs in HG002. Subsequently, we tested the data from different sequencing technologies at 0.5× coverage. For each case, we performed ten repetitions and recorded the mean, minimum, and maximum values of the score. As shown in Figure 4D, the stability of the variant sketch for low-coverage data decreases as the number of SNPs decreases, particularly for ONT data. We attribute this to its high error rate, which significantly reduces the number of effective sites. Therefore, we conservatively estimate that at least approximately 20k sites are required to maintain basic stability, ensuring at least 50 effective sites even for ONT data at a coverage as low as 0.5×. When the number of effective sites falls below 50, we issue an unreliability warning, urging the reselection of more SNP sites for testing. Coincidentally, we observed that tools like NGSCheckMate and somalier also utilize approximately 20,000 SNP sites to assess sample relationships, further underscoring the significance of this site count."
**Additional Information:**	
**Question**	**Response**
Are you submitting this manuscript to a special series or article collection?	No
**Experimental design and statistics**<br><br><br>Full details of the experimental design and statistical methods used should be given in the Methods section, as detailed in our Minimum Standards Reporting Checklist. Information essential to interpreting the data presented should be made available in the figure legends.	Yes

Powered by Editorial Manager® and ProduXion Manager® from Aries Systems Corporation

Have you included all the information requested in your manuscript?	
**Resources**<br><br>A description of all resources used, including antibodies, cell lines, animals and software tools, with enough information to allow them to be uniquely identified, should be included in the Methods section. Authors are strongly encouraged to cite Research Resource Identifiers (RRIDs) for antibodies, model organisms and tools, where possible.<br><br>Have you included the information requested as detailed in our Minimum Standards Reporting Checklist?	Yes
**Availability of data and materials**<br><br>All datasets and code on which the conclusions of the paper rely must be either included in your submission or deposited in publicly available repositories (where available and ethically appropriate), referencing such data using a unique identifier in the references and in the "Availability of Data and Materials" section of your manuscript.<br><br>Have you have met the above requirement as detailed in our Minimum Standards Reporting Checklist?	Yes
GigaScience has policies and guidelines in place for the use of generative AI-writing tools such as ChatGPT. If you have used such writing tools to assist with writing the manuscript this must be declared and cited in the text. Authors should not list AI-writing tools and other AI-assisted technologies as an author or co-author and should acknowledge that they are fully responsible for text generated or refined by AI-writing tools.<p>	Yes

A summary of use (particularly in the introduction or among methods) needs to be included at the end of the paper, and the outputs should also be included as a supplementary file hosted in GigaDB or other open repositories. Please read our guidelines for more information. <p><br><br>By submitting to GigaScience, you are aware of the journal's AI-writing tools policy, and if you have declared use of such tools below, you have acknowledged this where appropriate in your manuscript and have made a summary of use and outputs available. <p><br><b>AI-assisted writing tools have been used in the preparation of this manuscript?	

Powered by Editorial Manager® and ProduXion Manager® from Aries Systems Corporation

Manuscript
Click here to access/download;Manuscript;PISAD_Revision.pdf

GigaScience, 2023, 1–12

doi: xx.xxxx/xxxx
Manuscript in Preparation
Paper

PAPER

PISAD: reference-free intraspecies sample anomalies detection tool based on k-mer counting

Zhantian Xu[1], Fan Nie[2] and Jianxin Wang[1,*]

[1]School of Computer Science and Engineering, Central South University, Changsha 410083, China and [2]National Center for Applied Mathematics in Hunan and Key Laboratory of Intelligent Computing and Information Processing of Ministry of Education, Xiangtan University, Xiangtan 411105, China

[*]Jianxin Wang, School of Computer Science and Engineering, Central South University, Changsha 410083, China. E-mail: jxwang@mail.csu.edu.cn

Abstract

Background: Genomic sequencing research often requires the simultaneous analysis of heterogeneous data types across single or multiple individuals, introducing a substantial risk of sample swaps (e.g., labeling errors). Existing methods primarily rely on reference information, requiring the pre-selection of informative variant sites with a population allele frequency around 0.5, which may be insufficient or unavailable for non-model organisms. As research expands to encompass a growing number of new species, a robust quality control tool will become increasingly important.
Finds: We developed PISAD, a tool for validating sample identities in whole-genome sequencing (WGS) data without requiring reference information. It uses a two-stage approach: first, it performs rapid, reference-free SNP calling on low-error-rate data from the target individual to create a variant sketch; then, it assesses the concordance of other samples on this sketch to verify relationships. We assessed the performance and efficiency of PISAD on *Homo sapiens*, *Bos taurus*, *Gallus gallus*, *Arctia plantaginis* and *Pyrus* species.
Conclusion: Our evaluation showed that PISAD achieves a lower data coverage requirement (0.5×) compared to the reference-based tool ntsm and is broadly applicable to multiple diploid species.

Key words: Sample swap, SNP calling, Reference-free, K-mer analysis, Quality control.

Introduction

Whole-genome sequencing (WGS) studies often involve multiple or single individuals across various experiments using different sequencing technologies(e.g., Illumina, PacBio, Oxford Nanopore Technologies, Hi-C, etc.). For instance, de novo assembly often involves sequencing data from multiple technologies[1, 2, 3] to improve assembly quality. Moreover, sequencing data from each technology may involve multiple sequencing runs. Each new procedure or handling introduces potential opportunities for sample swap. Even a single sample swap can have severe consequences on downstream analyses. Therefore, confirming the relatedness of samples assumed to come from the same donor is an essential step in quality control(QC), which should be performed as early as possible in the analysis pipeline.

Existing sample swap detection methods can be divided into two categories based on the source of the sample: cross-species and same-species. Cross-species swaps have been extensively studied. For example, Mash[4] uses MinHash techniques to rapidly calculate the genomic distance to identify them. However, in same-species swaps, the high genetic similarity among samples can obscure the differences. Current approaches for detecting same-species swaps primarily rely on genotypes at single nucleotide polymorphisms (SNPs), leveraging predetermined variant sites constructed from population-level allele frequency to distinguish between samples[5, 6, 7, 8, 9, 10, 11, 12]. For instance, Peddy[6] extracts genotypes at preselected variant sites from VCF files for each sample and uses the kinship calculation method from KING[13] to determine the relationships between samples. At the same time, Somalier[10] accelerates relationship calculation between samples

Compiled on: April 2, 2025.
Draft manuscript prepared by the author.

by creating sketches for rapid comparison. NGSCheckMate[9] verifies sample identity in next-generation sequencing(NGS) data by calculating the variant allele fractions at preselected SNP sites using a model-based approach. CrossCheck[8] leverages linkage disequilibrium to achieve improved accuracy in shallow sequencing. ntsm[11] leverages *k*-mer counting and maximum likelihood estimation, making it suitable for low-coverage and heterogeneous whole-genome sequencing data.

While current approaches for detecting sample swaps have been successful across a wide range of applications, most are limited to human samples. Although ntsm introduces a method for extracting informative variant sites and optimistically suggests applicability to other species, its performance may degrade or even fail in species where research is still in its early stages and population-level allele frequency information is limited or unavailable.

To address this problem, we use a reference-free SNP calling approach to construct variation sketches, eliminating the need for predefined variant sites. Currently, two main methods are available for reference-free SNP calling. In hybrid approaches, raw reads are assembled into long contigs or scaffolds, and SNPs are then identified by aligning the raw reads to these assembled contigs and mapping them to specific positions [14, 15, 16]. The accuracy of this method depends heavily on assembly quality, and the assembly step itself is time-consuming [17]. The second approach processes data directly based on *k*-mer counting. For example, Cortex[18] and DiscoSnp++ [17, 19] construct a de Bruijn graph from raw data and detects specific patterns to call SNPs. ebwt2snp [20, 21] uses the extended Burrows-Wheeler Transform (eBWT) from reads to identify SNPs as pairs of *k*-mers. Kmer2SNP [22] simplifies the heterozygous SNP calling problem by finding the maximum weight matching in the heterozygous *k*-mer graph. Unlike previous methods, it selects *k*-mers from heterozygous regions based on *k*-mer frequency distribution rather than using all *k*-mers, substantially reducing data volume and largely mitigating the impact of homologous/repetitive sequences. However, the existing Kmer2SNP approach is not suitable for low-coverage data, and the speed and precision of SNP calling remain bottlenecks for downstream analysis, prompting us to introduce improvements.

In this work, we developed PISAD, a **P**hsaed **I**ntraspecies **S**ample **A**nomalies **D**etection tool, suitable for multiple species without reference information. The tool eliminates the need for reference information by using a reference-free SNP calling approach to construct variant sketches. Additionally, we improve the SNP calling method that is an order of magnitude faster and enable the detection of sample swaps using only heterozygous SNP information by refining the calculation of inter-sample relationships. Our process requires no additional reference information or downstream steps, such as alignment, making it an efficient QC tool for the upstream stage.

Methods

Algorithm overview

We developed PISAD, a tool designed to detect anomalies in cohort samples without requiring reference information. The tool operates in two primary stages. In stage 1, we performed reference-free SNP calling to construct a variant sketch using low-error-rate data from the target individual. In stage 2, we compared the *k*-mer counts of other cohort samples on the variant sketch to infer relationships between them (Fig. 1). It is important to note that Stage 1 of our tool supports only low-error-rate sequencing data, meaning most ONT data (except duplex) cannot be processed. However, Stage 2 is compatible with various sequencing technologies. Therefore, at least one low-error-rate dataset is required for reliable sketch construction.

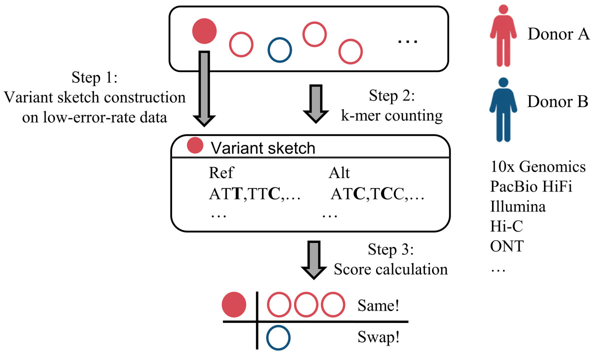

Figure 1. A schematic overview of PISAD. Red and blue circles represent sequencing runs from different donors with heterogeneous data types. The solid circle indicates the data used for variant sketch construction, which, in practice, can be any low-error-rate data (e.g., 10x Genomics, PacBio HiFi, etc.). The mix of blue and red circles represents sample swaps, indicating that samples are incorrectly assigned to the wrong donor.

SNP calling

In SNP calling, the first step is to select heterozygous *k*-mers to construct the vertex set. Kmer2SNP uses DSK[23] to count *k*-mer frequencies from raw reads and generate a corresponding *k*-mer histogram file. Then, FindGse[24] is employed to identify the frequency range of heterozygous *k*-mers. Existing genome analysis tools, such as FindGse, are typically designed for high-coverage data (>30×) and are not well-suited for shallow sequencing. Therefore, we developed a heuristic algorithm to estimate the range of heterozygous regions under low-coverage conditions.

The algorithm begins by reading the first 1000 entries from the histogram of *k*-mer abundances, which records the number of distinct *k*-mers for each occurrence frequency, and then checks for a sequence of three consecutive points that shows an upward trend followed by a downward trend. If such a sequence is found, it is defined as a peak. To filter out noise from small peaks, the algorithm will terminate early if either 95% of the total *k*-mer frequency has been read or if two peaks have already been identified. When two peaks are found, the first peak is assumed to represent the heterozygous peak. If only one peak is identified due to low coverage, an additional parameter is required to indicate whether the heterozygosity rate of the species is greater than 1.2%. This distinction is necessary because, with only one peak, the algorithm cannot reliably determine whether it represents a homozygous or heterozygous region. According to GenomeScope[25], when the heterozygosity rate exceeds about 1.2%, the frequency of the heterozygous peak begins to surpass that of the homozygous peak. Once the heterozygous peak value is identified, the heterozygous region is calculated.

Theoretically, heterozygous region distribution adheres to a Poisson or negative binomial distribution ranging from 0 to infinity. However, directly selecting *k*-mers from these regions 1) greatly increases algorithm runtime and memory consumption and 2) generates numerous false SNPs from chimeric sequences (overlapping *k*-mers in the SNP sequence actually originating from different reads) and homologous/repetitive sequences. Inspired by the Kmer2SNP concept, we heuristically designate heterozygous peaks of 0.5×–1.5× as heterozygous regions (minimum left boundary of 2 to exclude erroneous *k*-mers), ensuring the capture of most *k*-mers in these regions while maximally reducing runtime, memory usage, and false SNPs. Finally, we fitted the *k*-mer frequency distribution using two negative binomial distributions[25, 26] and provided a rough estimate of genomic characteristics to assist users in evaluating the correctness of the algorithm's selection. The results of heterozygous region selection are shown in Fig. 2A, Fig. S1.

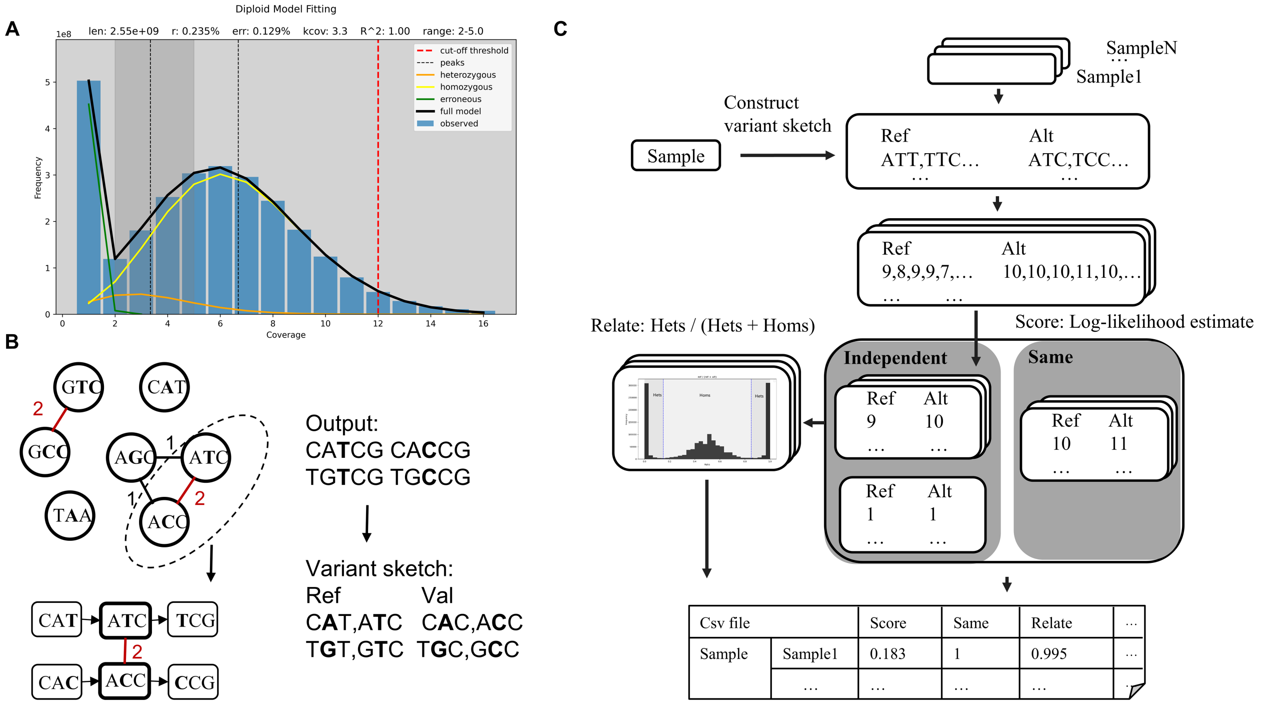

Figure 2. Illustration of key steps. (A) A schematic diagram illustrating the algorithm's selection of heterozygous regions, using HG002 data at 6× coverage as an example. The dark gray areas represent the designated heterozygous regions, while the top of the figure displays genomic characteristics estimated using two negative binomial distributions. (B) Stage 1: Calling isolated SNPs on low-error-rate data to construct a variant sketch. The left-side diagram illustrates *k*-mers located in heterozygous regions (represented as circles). The connecting lines between the circles indicate *k*-mer pairings (with a single nucleotide difference in the middle), and the numbers on these lines represent the support length (i.e., the maximum possible expansion distance). The red-colored SNPs indicate the isolated SNPs ultimately selected through maximum-weight matching. (C) Stage 2: Using *k*-mer counting based on the variant sketch to determine the relationship between samples. The input includes the low-error-rate data used for sketch construction and other samples to be tested. The output is a CSV file listing relationships between samples.

After obtaining the *k*-mer data for the heterozygous regions, the next step is to call SNPs in these regions. KmerSNP first identifies *k*-mers within heterozygous regions that differ by only a single nucleotide in the middle and designates them as potential SNPs. It then expands both sides of each potential SNP, where the maximum possible expansion distance is defined as the support length. Finally, the algorithm selects the final SNPs by computing the maximum-weight matching of all potential SNPs (Fig. 2B).

Although it has demonstrated the best performance among reference-free SNP calling tools, we observed that for whole-genome SNP calling, its runtime often exceeds one hour, and the precision of SNP calling drops sharply as coverage decreases, which is unacceptable for our requirements. Excessive SNP calling time considerably increases the operational cost of using the tool, while a high number of incorrect SNP calls can severely impact our tool's performance. To address these issues, we restructured and optimized the algorithm in C++, incorporating parallel-hash [27] for extensive parallel computation. To further enhance SNP calling precision, we selected only SNPs supported by a length of 21. Our improved Kmer2SNP algorithm only calls isolated SNPs, as these SNPs are independent in subsequent analyses. Since no reference genome is available, all called SNPs are heterozygous.

Sketch construction

After SNP calling, a variant sketch FASTA file is constructed (Fig. 2B). Each called SNP is split into Ref and Alt columns and processed separately using a 21-mer sliding window. Each *k*-mer is hashed, and identical or reverse-complement *k*-mers are removed to ensure the independence. When validating relationships between multiple individuals in large cohorts, the process can be repeated for each individual, and the resulting sketches can be merged into a comprehensive sketch for analysis.

Variant *k*-mer counting

After obtaining and reading the variant sketch FASTA file, we hash it into a hash table using a reversible hash function. Input sequences in FASTQ format are decomposed into *k*-mers, which are subsequently hashed. Whenever a *k*-mer matches an entry in the hash table, its read count is incremented by one. These read counts are then used to compute the final score (Fig. 2C). Optionally, the process can be terminated early by specifying an expected coverage threshold to optimize runtime.

Calculating Score

We employed two methods to calculate the relatedness coefficient: relatedness score and likelihood score.The relatedness score provides a detailed measurement of the relationship between samples at higher coverage, while the likelihood score is designed to robustly verify whether two samples are identical under low coverage conditions, primarily to detect sample swaps.

To calculate the relatedness score, allele counts at each site for each sample are first converted into genotypes. Each site includes multiple counts for reference (Ref) and alternative (Alt) alleles. The most frequent count is selected as the genotype for each site to obtain a reliable estimate. Next, the Ref/Alt ratio is calculated, then plotting a histogram to identify the cut-off point at the lowest frequency in the histogram. This cut-off is subsequently used to classify sites as heterozygous or homozygous (Fig. 2C).

After determining the genotype for each site, we calculate the relatedness score based on the differences in observed genotypes

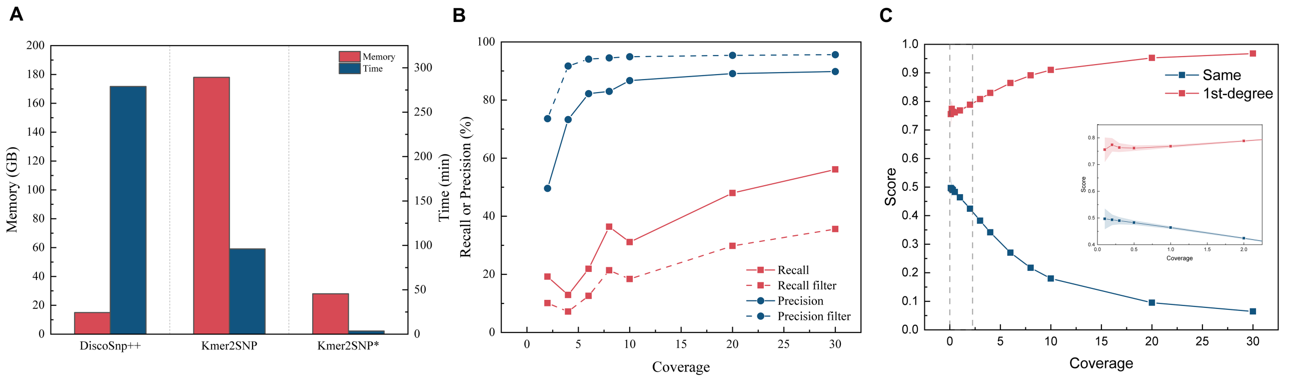

Figure 3. SNP calling and simulation results. (A) The performance of reference-free SNP calling tools. Each tool only calls isolated SNPs, with other settings following default parameters. DiscoSnp++ and Kmer2SNP* use 8 cores, while Kmer2SNP only supports 1 core. Kmer2SNP* only involves optimizations for time and memory consumption, and its SNP calling results remain consistent with Kmer2SNP. The time statistics do not include the *k*-mer counting step(DSK). (B) The results of SNP calling performance after filtering by selecting SNPs supported by a length of 21.(C) Sample swap scores based on simulations. Sequencing depth distribution is simulated using a Poisson distribution, while depth at each heterozygous site follows a binomial distribution with p=0.5. The shaded areas around each line represent the 1st and 99th percentiles of the simulated scores.

between each pair of samples. Existing methods, such as KING[13], rely on the IBS0 statistic, which represents the number of loci where a pair of individuals share zero alleles. For related individuals, such as parent-offspring or siblings, their IBS0 should never be zero unless Mendelian inheritance is violated. However, unlike typical scenarios, our variant sketch includes only heterozygous SNPs from each sample, with no information on homozygous SNPs. Therefore, we define the relatedness score calculation as follows:

$$\frac{Het_i}{Het_i + Hom_i} \qquad (1)$$

Here, i represents the sample to be tested, while Het_i and Hom_i are the counts of heterozygous and homozygous sites for sample i, respectively. Sample j is the reference sample used to create the variant sketch. We only observe the counts of sample i, as sample j originates from the SNP calling of the target individual and consists entirely of heterozygous sites. In this context, if two samples are identical, sample i should share all heterozygous SNPs with sample j, and thus the relatedness score is equal to 1. If there is a parent-offspring or sibling relationship between the samples, they should share half of the heterozygous SNPs, resulting in a relatedness score of 0.5. Due to the absence of homozygous SNP information, our tool is currently unable to detect more distant relationships.

Under low data coverage, distinguishing between heterozygous and homozygous genotypes becomes challenging. Therefore, we largely borrow from the exact method described in the ntsm publication. This method employs maximum likelihood estimation and log-likelihood ratio test to determine whether two samples are identical[28]. It assumes two models: one in which the samples are independent and another in which they are same. A multinomial-like likelihood function is used, and the log-likelihood ratio between the two models is calculated to provide a robust assessment of sample identity (Fig. 2C).

The key differences between our method and ntsm are as follows. First, in the likelihood ratio test, we introduce two samples: one is the query sample, while the other is modified such that all ref/alt values are set to 1. This approach incorporates a predefined distribution of identical samples, allowing for a comparative assessment of the query sample. Second, we remove the two empirical bias parameters in ntsm, and the final score is the mean log-likelihood ratio across all sites, as follows:

$$\overline{\lambda_{\mathrm{LR}}} = -2\log\frac{\mathcal{L}^{(*)}}{\mathcal{L}^{(1)}\cdot\mathcal{L}^{(2)}} * \frac{1}{N} \qquad (2)$$

Here, $\overline{\lambda_{\mathrm{LR}}}$ represents the mean of log-likelihood ratio test, N represents the effective number of sites, $\mathcal{L}^{(1)}$ and $\mathcal{L}^{(2)}$ represent the likelihood values of the two samples when considered independently, respectively. $\mathcal{L}^{(*)}$ represents the likelihood value when considered identical. Additionally, it is noteworthy that we only consider sites where the sum of Ref and Alt values is greater than or equal to 2 (which we define as effective sites) to minimize the impact of missing data due to low coverage.

Results

Simulation

Simulations were conducted to investigate the impact of various factors on our method. We first assumed that the variant sketch contained 200k heterozygous sites. Then, we used a Poisson distribution to simulate the depth distribution under different coverage levels (0.1, 0.2, 0.3, 0.5, 1, 2, 3, 4, 6, 8, 10, 20, 30x).

For the same individual, a binomial model with p = 0.5 was used to simulate all sites. For individuals with a first-degree kinship, half of the sites were simulated using the binomial model, while for the other half, the reference (ref) and alternate (alt) alleles were randomly assigned, with one placed at the simulated depth and the other set to zero.

Each coverage level was simulated 1000 times, and the mean, 1st percentile, and 99th percentile values were recorded. As shown in Fig. 3C, the discriminability between the two cases improves with increasing coverage. Finally, we set a threshold score of 0.63 to determine whether the two samples are identical.

SNP calling

We evaluated the performance of the improved Kmer2SNP using PacBio High-Fidelity (HiFi) sequencing data from the HG002 sample at a depth of 30x. As shown in Fig. 3A, our tool achieves considerably faster SNP calling, requiring only 3.5 minutes and 28 GB of memory. Compared to the original Kmer2SNP algorithm, this achieves a 25.2-fold increase in speed and a 6.3-fold reduction in memory consumption. Although DiscoSnp++ benefits from using a Bloom filter, which reduces its memory usage to around 15 GB, its runtime of nearly 4.65 hours makes our tool a more efficient choice.

Subsequently, we analyzed the selection of k-mer sizes from two perspectives. For SNP calling performance, increasing k enhanced overall performance, though it also raised memory usage and runtime. Starting at 21-mer, it achieved high-quality results,

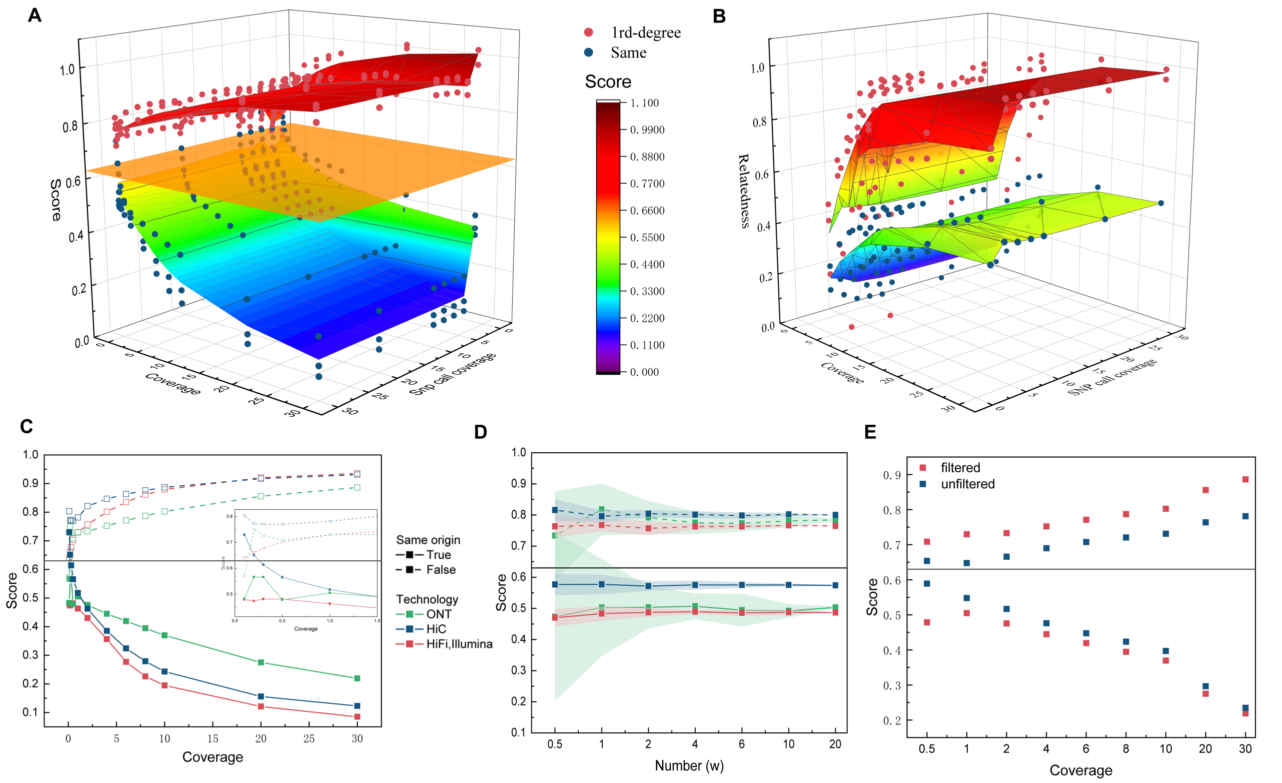

Figure 4. The impact of data coverage. (A) Likelihood score results for different input and SNP calling data coverages. Blue and red points indicate results for the same and different samples. The x-axis represents the coverage of the data being tested, while the y-axis denotes SNP call coverage, referring to the coverage used for SNP calling and variant construction. The orange plane represents the threshold (t=0.63) used by our method to determine whether samples are identical . (B) Relatedness score results under the same conditions as (A). Blue and red points represent results for first-degree relationships and identical relationships, respectively. (C)Cross-section of Figure 4A with the SNP call coverage axis at a value of 4, where different colors represent different sequencing technologies. (D) The variation in sample swap scores under different numbers of heterozygous SNPs. Each experiment is simulated ten times, and the shaded area around each line represents the range between the maximum and minimum scores. (E) The results of detecting sample swaps on filtered and unfiltered sketches. The horizontal line in the figure represents the threshold for determining whether samples are identical. To achieve better discrimination, the two categories of samples should be as far from the line as possible.

which then showed gradual improvement as k increased further (Table S1). In terms of tolerance to high-error data in stage 2, smaller k-mers provide greater redundancy to compensate for sequence errors (Fig. 2C). To balance these factors, we chose a 21-mer, which achieves sufficient SNP calls, shorter runtime, and enhanced performance with high-error-rate data.

Finally, we evaluated SNP calling results across different coverages by using seqkit[29] to subsample HG002 PacBio HiFi sequencing data to the depth(x) of 2, 4, 6, 8, 10, 20, and 30. Fig. 3B shows that both recall and precision improve as coverage increases. However, we found that the precision of SNP calling was insufficient, with a large number of erroneous calls occurring at low coverage levels, especially below 10x. After filtering by selecting only SNPs supported by a length of 21, precision remained above 90% for coverages of 4x and higher, at the cost of some recall.

Impact of SNP sites

To evaluate the impact of increased precision and reduced recall on SNP calling results after filtering, we first conducted experiments using 4x coverage SNP calls and ONT(R9.4.1, accuracies: 85.6%, 87.7%) data from HG002 and HG003. We chose ONT data due to its high error rate, anticipating that it would present the greatest analytical challenge. As shown in Fig. 4E, the filtered variant sketch displays marked enhanced discriminatory power compared to the unfiltered version, enabling the tool to make more robust assessments.

To further investigate the impact of SNP sites used in Stage 1 on the results, we used the previously mentioned HG002 PacBio HiFi data with 2×–30× coverage to create the variant sketch. Meanwhile, we selected downsampled data from HG002 and HG003, including PacBio HiFi, Hi-C, and ONT(R9.4.1) sequencing technologies, with coverage ranging from 0.1× to 30×, as the test data for Stage 2.

In the data coverage utilized for SNP calling, we found that neither the likelihood nor the relatedness score is particularly sensitive to coverage levels in stage 1 (Fig. 4A,B). The only substantial performance drop occurred at 2x coverage. As shown in Fig. 3B, 2x coverage is also the only condition where precision falls below 90%, suggesting that an excess of erroneous SNP calls reduces tool performance. For coverages above 2x, although the number of SNP calls increased, this increase provided minimal benefit to tool performance compared with the importance of maintaining high precision.

Meanwhile, we further investigated the number of SNPs used in the variant sketch in stage 1. From all heterozygous SNPs called at 30× coverage in HG002, we randomly sampled 5k, 10k, 20k, 40k, 60k, 100k, and 200k SNP sites, corresponding to 0.25%, 0.5%, 1%, 2%, 3%, 5%, and 10% of the total heterozygous SNPs in HG002. Subsequently, we tested the data from different sequencing technologies at 0.5× coverage. For each case, we performed ten repetitions and recorded the mean, minimum, and maximum values of the score. As shown in Fig. 4D, the stability of the variant sketch for low-coverage data decreases as the number of SNPs decreases, particularly for ONT data. We attribute this to its high error rate, which significantly reduces the number of effective sites. Therefore, we conservatively estimate that at least approximately 20k sites are required to maintain basic stability, ensuring at least 50 effective sites even for ONT data at a coverage as low as 0.5×. When

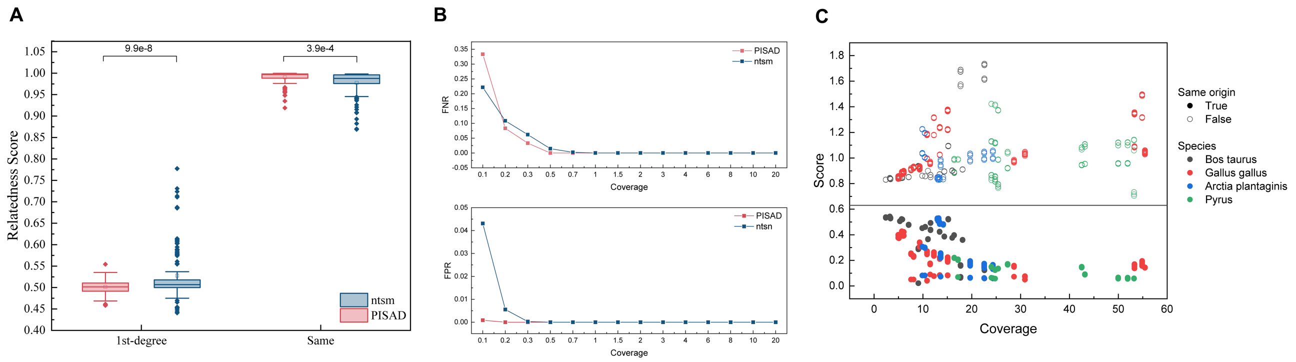

Figure 5. Evaluation results on human and other species. (A) Relatedness score results of ntsm and PISAD for 20 human family trio samples. Levene's test is used to perform significance analysis on the variance of the data. (B) False negative rate (FNR) and false positive rate(FPR) of PISAD and ntsm at varying raw dataset coverage in 20 human samples. PISAD constructs variant sketches using approximately 15x coverage Illumina data for each sample. ntsm uses the lower coverage between the two compared samples as the x-axis value. (C) Results of identifying identical samples in trio data across different species. The x-axis represents the coverage of the sample with the lower coverage between the two input samples. The gray horizontal line denotes the threshold for determining sample identity.

Table 1. Detailed information on the sample swap experiments for each species.

Species[a]	Tissue	Sequencing technology[b]	Depth	Individual	Variant sketch[c]	Sample	match, unmatch[d]
Homo sapiens(0.31%)	Lymphoblastoid cell lines	Illumina, PacBio HiFi, Hi-C, ONT(R9.4.1, 91.1%)	0.1 - 20x	20	20	840	833, 15967
Bos taurus(1.12%)	Frozen lung	Illumina, Hi-C, ONT(R9.4.1, 85.2%)	2.4 - 22.6x	3	6	26	52, 104
Gallus gallus(1.03%)	Blood	Illumina, Pacbio HiFi, Hi-C, 10x Genomics	4.9 - 55.4x	3	18	40	230, 490
Arctia plantaginis(1.90%)	Pupae	Illumina, Pacbio CLR, 10x Genomics	9.8 - 24.3x	3	12	17	112, 88
Pyrus(1.6%, 2.0%)	Leaf	Illumina, Pacbio HiFi, ONT(R9.4.1,93.7%)	17.1 - 53.2x	5	10	18	47, 133

[a] The values in parentheses represent the estimated heterozygosity, which was estimated using GenomeScope2[30] based on offspring sequencing data with coverage exceeding 30×. For Purus, two separate estimates were obtained due to the presence of two offspring.

[b] The values in parentheses represent the flow cell version and the average accuracy of the ONT data.

[c] It represents the number of data used to construct the variant sketch, which includes all Illumina data for humans and all low-error-rate data for other species.

[d] It represents the number of matching and non-matching experiments in the total number of experiments, where the total number of experiments is equal to the number of variant sketches multiplied by the number of query samples.

the number of effective sites falls below 50, we issue an unreliability warning, urging the reselection of more SNP sites for testing. Coincidentally, we observed that tools like NGSCheckMate[9] and somalier[10] also utilize approximately 20,000 SNP sites to assess sample relationships, further underscoring the significance of this site count.

Impact of stage 2 coverage

In contrast to the minimal impact of stage 1 coverage, we found that data coverage in stage 2 has a considerable effect on the tool's performance. As coverage increases, the tool's ability to measure relationships improves accordingly. For calculating relatedness score to assess the relationship between two samples, we recommend a minimum coverage of 20x to ensure robust estimates (Fig. 4B). However, if the objective is simply to calculate the likelihood score to determine whether the two samples are identical, a much lower coverage level is sufficient. Concurrently, we noted that ONT data posed the greatest challenge in detecting sample swaps, as its elevated error rate diminishes effective site counts, requiring increased coverage for enhanced differentiation. Additionally, we found that the tool performs well across various sequencing data types at coverage levels above 0.5×(Fig. 4A,C).

Comparisons to ntsm

To evaluate the performance of our tool, we selected well-studied human samples with extensive and reliable reference information. This choice enabled us to compare our tool with the state-of-the-art reference-based sample swap detection tool, ntsm.

To validate sample swaps, we used 20 samples from the Human Pangenome Reference Consortium (HPRC) [31], which includes sequencing data from Illumina, PacBio HiFi, Hi-C, and ONT(R9.4.1) platforms. We then constructed the variant sketch using Illumina data from each sample, while the remaining data were downsampled to coverage levels ranging from 0.1× to 20× to evaluate the performance of our tool and ntsm under varying coverage levels. Details of these datasets are provided in Table S2. Additionally, to calculate relatedness, we supplemented this dataset with samples from the 1000 Genomes Project [32], combining it with HPRC sample to create a dataset of 20 family trios(Supplementary Table S1). Each child in the trios has data from multiple sequencing technologies, while the parents have only Illumina data.

Detecting the sample swap

We evaluated the performance of both tools across different data coverage levels. We found that both tools could detect samples with coverage higher than 1×. However, our tool was able to further detect samples with coverage as low as 0.5×(Fig. 5B, Fig. S3). Not surprisingly, as noise begins to dominate the correlation, performance declines at very shallow depths (<0.5×). We infer that although our method lacks homozygous SNP sites, which provide better discrim-

inative power in shallow sequencing[33], the sample-specific sites generated by our approach result in a greater number of effective sites compared to the universal sites selected by ntsm. As a result, this leads to improved performance.

Calculating relatedness

For the relatedness score, we found that our tool demonstrated tighter grouping compared to ntsm (Fig. 5A). Further analysis revealed that most of the poorly estimated results in ntsm were derived from ONT sequencing, likely due to its high error rates. Our method differs from ntsm in two key aspects. First, when merging multiple counts at each site, we use the mode instead of the maximum value. For example, if we observe counts like 8, 9, 8, 7 at a given site, we take the mode (which would be 8) rather than the maximum (which would be 9). Second, instead of using a fixed threshold to determine the genotype, we determine the threshold by identifying the minimum value of the Ref/(Ref+Alt) spectrum within a certain range (Fig. 2C). We believe these modifications help mitigate the impact of high error rates, leading to a more stable and compact genotype distribution. However, due to the absence of homozygous SNP information in our method, it lacks the capability to distinguish relationships beyond 1st-degree (parent-offspring and siblings), unlike reference-based tools.

Extension to other species

To assess the performance of our tool on other species in practical application scenarios, we selected family trio samples from three species: *Bos taurus*, *Gallus gallus*, *Arctia plantaginis* and *Pyrus*. The first two species were obtained from the Vertebrate Genome Project (VGP)[34], while the third was obtained from a study that performed de novo assembly of *Arctia plantaginis* through trio binning[1]. The last one is derived from a study on T2T genome assembly of two pear hybrid cultivars, 'Yuluxiang' (YLX) and 'Hongxiangsu' (HXS)[35]. Details of these datasets are provided in Table S2.

For each species, we first created sketches on all low-error-rate runs. We then validated all data runs against each sketch to evaluate the performance of our tool in real-world scenarios. As shown in Fig. 5C, for species with different heterozygosity, such as *Bos taurus*, *Gallus gallus*, *Arctia plantaginis* and *Pyrus*. our tool is able to identify all identical and non-identical samples correctly. Additionally, as coverage increased, the results became more confident.

Running time

Since a large number of files may need to be checked, the speed of the algorithm is crucial. In stage 1, the time consumption mainly stems from two processes: (1) k-mer histogram counting, which uses the DSK algorithm to extract k-mers located in heterozygous regions, and (2) calling algorithm, which performs SNP calling based on these filtered k-mers. The time required for k-mer histogram counting increases with the amount of data (Fig. S4A) .For the calling algorithm, memory and time consumption largely depend on the number of heterozygous-region k-mers identified in the k-mer histogram counting phase. At lower data depths, the boundaries of heterozygous regions are less well-defined, which introduces a large amount of irrelevant data and increases both time and memory consumption. However, this improves with deeper coverage. In stage 2, we observed that the runtime increases almost linearly with the depth of data (Fig. S4B). Additionally, it supports parallel processing of multiple files.

For a typical run of low-error-rate sequencing data, the coverage is approximately 10x. Under these conditions, the total time for stage 1 is roughly 10 minutes. Additionally, our performance evaluation shows that comparable results can be achieved with coverage as low as 0.5x. In this case, the computation time for a single sample in stage 2 is approximately 2 minutes. Therefore, testing a pair of samples requires only 12 minutes.

Disscussion

Comparisons to reference-free tools

Although our tool, like generic k-mer comparison methods such as Mash, does not require reference information, there are significant differences in the workflow. Mash operates by extracting a subset of sequences with the some smallest hash values from the data for comparison. In contrast, our method essentially extracts SNP information from the data for comparison. This difference leads to two key distinctions. First, thanks to the use of SNP information, our method can identify intraspecies sample swaps, whereas Mash is generally limited to distinguishing samples between distinct species. However, our approach is also constrained by the requirements of SNP extraction, which necessitates low-error-rate data with sufficient coverage, even if we can avoid performing SNP calling for each sample by creating sketches. On the other hand, Mash has almost no requirements on the data, making it compatible with various data types. Additionally, in terms of computation, extracting a subset of sequences and performing statistics on them is simpler than performing SNP calling, indexing, and comparing sequences between samples.

Comparsions to reference-base tools

The primary difference between our tool and reference-based methods like ntsm is that we replace the need for pre-defined variant sites with a step of reference-free SNP calling and variant sketch construction, allowing our tool to be applied to multiple diploid species rather than being limited to human.According to our performance evaluation, we achieved a lower data coverage requirement compared to ntsm, requiring only 0.5× coverage. However, this comes at the cost of requiring additional high-accuracy sequencing data for constructing the variant sketch.

In terms of time consumption, our method generally performs similarly to ntsm. The main difference is the additional step in stage 1, where SNP calling and sketch construction take place, which can be completed in about 10 minutes. Additionally, unlike stage 2, where k-mer counting is required for each sample, to validate a batch of samples and check if they belong to the same individual, we only need one low-error-rate data from the target individual for SNP calling and variant sketch construction.

Current limitations

In stage 1 of our tool, although we have optimized the reference-free SNP calling algorithm to better accommodate low-coverage data and achieve faster SNP calling, its memory consumption, which often exceeds 40 GB, remains challenging for small-memory servers. Additionally, our SNP calling method is still only suitable for low-error-rate data. Since our approach relies solely on k-mers rather than alignment to a reference genome, it has difficulty distinguishing between base errors and true base variants. This limitation means that at least one low-error-rate data from the target individual is required to use our tool effectively. Regarding the scope of application, our method primarily focuses on detecting sample swaps in WGS data and has not yet been tested on other data types such as whole-exome data[36], RNA sequencing[37], or ChIP sequencing[38]. While we are optimistic about the principle behind it (i.e., the use of SNP information), variations in sequencing regions, such as reduced-representation seq, may require a greater number of SNP sites to ensure the tool's effectiveness. Meanwhile, calling SNPs from such data without reference poses greater challenges to precision compared to WGS data. Due to the absence of designated heterozygous regions, the k-mer-based method resulted in a higher incidence of false SNPs from homologous/repetitive sequences, yielding a precision of only 70%–80%. This will also constrain the tool's resolution capability, warranting further inves-

tigation. Additionally, our current study is exclusively focused on diploid species. Polyploid organisms have not been included in this investigation due to the inherent challenges in SNP calling associated with their complex genomic architectures, which warrant separate and more extensive future research.

Conclusion

We have developed PISAD to detect intraspecies sample swaps in heterogeneous data cohorts without reference information and have demonstrated its effectiveness in multiple diploid species. It achieves excellent performance even for datasets with sequencing depths as low as 0.5x and with multiple sequencing technologies. We believe that our tool, which neither requires additional reference information nor downstream analyses like alignments, can be easily integrated into upstream data production pipelines as an efficient QC process.

Availability of source code and requirements

Lists the following:

- Project name: PISAD
- Project home page: https://github.com/ZhantianXu/PISAD
- Operating system(s): linux
- Programming language: C++, Python
- Other requirements: TBD
- License: MIT
- BiotoolsID: pisad
- RRID: SCR_026598

Data availability

The specific workflow of this work can be found at workflowhub.[39]. The Illumina, PacBio HiFi, Hi-C, and ONT sequencing data for 20 human family trios are available at Amason S3 under the HPRC/ and HPRC_PLUS/ directories[40]. Details of the selected samples are shown in Supplementary Table S2.

For the coverage experiments, the Illumina, PacBio HiFi, and ONT sequencing data for HG002 and HG003 are available at the National Center for Biotechnology Information (NCBI)[41]. The Hi-C data for HG002 can be accessed at at Amason S3[42].

The data for species *Bos taurus*, *Gallus gallus*, and *Arctia plantaginis* are available from NCBI under the following project accession numbers: PRJNA677946, PRJNA1149711, PRJNA1150343, and PRJEB36595. The Pyrus data are available in the National Genomics Data Centerunder BioProject accession PRJCA022120 with CRA accession CRA013997.

Declarations

List of abbreviations

WGS: whole-genome sequencing studies; SNP: single nucleotide polymorphism; eBWT:extended Burrows-Wheeler Transform; Ref: reference alleles; Alt: alternative alleles; bp: base pair; HPRC: Human Pangenome Reference Consortium; MLE: maximum likelihood estimation; ONT: Oxford Nanopore Technology; FPR: false positive rate; FNR: false negative rate; QC: quality control; tsv: tab-separated values; VCF: variant call format;VGP: Vertebrate Genome Project.

Consent for publication

Not applicable

Competing Interests

The authors declare they have no competing interests.

Funding

This work is supported by the National Natural Science Foundation of China (No. 62332020).

Author's Contributions

J.X.W., F.N. and Z.T.X conceived and designed this project. Z.T.X implemented PISAD. Z.T.X and F.N. performed the evaluations and bioinformatics analysis. Z.T.X drafted the manuscript, J.X.W and F.N. contributed to proofreading. All authors read and approved the final manuscript.

Disclosure of use of AI-assisted tools including generative AI

The authors declare that AI tools (ChatGPT - 4o) were used solely for language refinement, and the manuscript has been reviewed by all authors to ensure the accuracy of its content[43].

Acknowledgments

We are grateful for resources from the High-Performance Computing Center of Central South University. The authors thank the anonymous reviewers for their valuable suggestions.

References

1. Yen EC, McCarthy SA, Galarza JA, Generalovic TN, Pelan S, Nguyen P, et al. A haplotype-resolved, de novo genome assembly for the wood tiger moth (Arctia plantaginis) through trio binning. GigaScience 2020;9(8):giaa088.
2. Cheng H, Concepcion GT, Feng X, Zhang H, Li H. Haplotype-resolved de novo assembly using phased assembly graphs with hifiasm. Nature methods 2021;18(2):170–175.
3. Kronenberg ZN, Rhie A, Koren S, Concepcion GT, Peluso P, Munson KM, et al. Extended haplotype-phasing of long-read de novo genome assemblies using Hi-C. Nature Communications 2021;12(1):1935.
4. Ondov BD, Treangen TJ, Melsted P, Mallonee AB, Bergman NH, Koren S, et al. Mash: fast genome and metagenome distance estimation using MinHash. Genome biology 2016;17:1–14.
5. Bergmann EA, Chen BJ, Arora K, Vacic V, Zody MC. Conpair: concordance and contamination estimator for matched tumor–normal pairs. Bioinformatics 2016;32(20):3196–3198.
6. Pedersen BS, Quinlan AR. Who's who? Detecting and resolving sample anomalies in human DNA sequencing studies with peddy. The American Journal of Human Genetics 2017;100(3):406–413.
7. Schröder J, Corbin V, Papenfuss AT. HYSYS: have you swapped your samples? Bioinformatics 2017;33(4):596–598.
8. Javed N, Farjoun Y, Fennell TJ, Epstein CB, Bernstein BE, Shoresh N. Detecting sample swaps in diverse NGS data types using linkage disequilibrium. Nature Communications 2020;11(1):3697.

9. Lee S, Lee S, Ouellette S, Park WY, Lee EA, Park PJ. NGSCheckMate: software for validating sample identity in next-generation sequencing studies within and across data types. Nucleic acids research 2017;45(11):e103–e103.
10. Pedersen BS, Bhetariya PJ, Brown J, Kravitz SN, Marth G, Jensen RL, et al. Somalier: rapid relatedness estimation for cancer and germline studies using efficient genome sketches. Genome medicine 2020;12:1–9.
11. Chu J, Rong J, Feng X, Li H. ntsm: an alignment-free, ultra-low-coverage, sequencing technology agnostic, intraspecies sample comparison tool for sample swap detection. GigaScience 2024;13:giae024.
12. Wang PP, Parker WT, Branford S, Schreiber AW. BAM-matcher: a tool for rapid NGS sample matching. Bioinformatics 2016;32(17):2699–2701.
13. Manichaikul A, Mychaleckyj JC, Rich SS, Daly K, Sale M, Chen WM. Robust relationship inference in genome-wide association studies. Bioinformatics 2010;26(22):2867–2873.
14. Bankevich A, Nurk S, Antipov D, Gurevich AA, Dvorkin M, Kulikov AS, et al. SPAdes: a new genome assembly algorithm and its applications to single-cell sequencing. Journal of computational biology 2012;19(5):455–477.
15. Li H, Durbin R. Fast and accurate short read alignment with Burrows–Wheeler transform. bioinformatics 2009;25(14):1754–1760.
16. Van der Auwera GA, Carneiro MO, Hartl C, Poplin R, Del Angel G, Levy-Moonshine A, et al. From FastQ data to high-confidence variant calls: the genome analysis toolkit best practices pipeline. Current protocols in bioinformatics 2013;43(1):11–10.
17. Uricaru R, Rizk G, Lacroix V, Quillery E, Plantard O, Chikhi R, et al. Reference-free detection of isolated SNPs. Nucleic acids research 2015;43(2):e11–e11.
18. Iqbal Z, Caccamo M, Turner I, Flicek P, McVean G. De novo assembly and genotyping of variants using colored de Bruijn graphs. Nature genetics 2012;44(2):226–232.
19. Peterlongo P, Riou C, Drezen E, Lemaitre C. DiscoSnp++: de novo detection of small variants from raw unassembled read set (s). BioRxiv 2017;p. 209965.
20. Prezza N, Pisanti N, Sciortino M, Rosone G. SNPs detection by eBWT positional clustering. Algorithms for Molecular Biology 2019;14:1–13.
21. Prezza N, Pisanti N, Sciortino M, Rosone G. Variable-order reference-free variant discovery with the Burrows-Wheeler Transform. BMC bioinformatics 2020;21:1–20.
22. Li Y, Patel H, Lin Y. Kmer2SNP: reference-free SNP calling from raw reads based on matching. In: 2020 IEEE International Conference on Bioinformatics and Biomedicine (BIBM) IEEE; 2020. p. 208–212.
23. Rizk G, Lavenier D, Chikhi R. DSK: k-mer counting with very low memory usage. Bioinformatics 2013;29(5):652–653.
24. Sun H, Ding J, Piednoël M, Schneeberger K. findGSE: estimating genome size variation within human and Arabidopsis using k-mer frequencies. Bioinformatics 2018;34(4):550–557.
25. Vurture GW, Sedlazeck FJ, Nattestad M, Underwood CJ, Fang H, Gurtowski J, et al. GenomeScope: fast reference-free genome profiling from short reads. Bioinformatics 2017;33(14):2202–2204.
26. Sarmashghi S, Bohmann K, P Gilbert MT, Bafna V, Mirarab S. Skmer: assembly-free and alignment-free sample identification using genome skims. Genome biology 2019;20:1–20.
27. Popovitch G, A family of header-only, very fast and memory-friendly hashmap and btree containers.p;. Accessed: 13/11/2024. https://github.com/greg7mdp/parallel-hashmap.
28. Fisher RA. On the mathematical foundations of theoretical statistics. Philosophical transactions of the Royal Society of London Series A, containing papers of a mathematical or physical character 1922;222(594-604):309–368.
29. Shen W, Le S, Li Y, Hu F. SeqKit: a cross-platform and ultrafast toolkit for FASTA/Q file manipulation. PloS one 2016;11(10):e0163962.
30. Ranallo-Benavidez TR, Jaron KS, Schatz MC. GenomeScope 2.0 and Smudgeplot for reference-free profiling of polyploid genomes. Nature communications 2020;11(1):1432.
31. Liao WW, Asri M, Ebler J, Doerr D, Haukness M, Hickey G, et al. A draft human pangenome reference. Nature 2023;617(7960):312–324.
32. Consortium GP, Auton A, Brooks L, Durbin R, Garrison E, Kang H. A global reference for human genetic variation. Nature 2015;526(7571):68–74.
33. Hemstrom W, Grummer JA, Luikart G, Christie MR. Next-generation data filtering in the genomics era. Nature Reviews Genetics 2024;p. 1–18.
34. Rhie A, McCarthy SA, Fedrigo O, Damas J, Formenti G, Koren S, et al. Towards complete and error-free genome assemblies of all vertebrate species. Nature 2021;592(7856):737–746.
35. Li Q, Qiao X, Li L, Gu C, Yin H, Qi K, et al. Haplotype-resolved T2T genome assemblies and pangenome graph of pear reveal diverse patterns of allele-specific expression and the genomic basis of fruit quality traits. Plant Communications 2024;5(10).
36. Albert TJ, Molla MN, Muzny DM, Nazareth L, Wheeler D, Song X, et al. Direct selection of human genomic loci by microarray hybridization. Nature methods 2007;4(11):903–905.
37. Wang Z, Gerstein M, Snyder M. RNA-Seq: a revolutionary tool for transcriptomics. Nature reviews genetics 2009;10(1):57–63.
38. Johnson DS, Mortazavi A, Myers RM, Wold B. Genome-wide mapping of in vivo protein-DNA interactions. Science 2007;316(5830):1497–1502.
39. Xu Z, PISAD - Phsaed Intraspecies Sample Anomalies Detection tool; 2025. https://doi.org/10.48546/WORKFLOWHUB.WORKFLOW.1322.1. WorkflowHub.
40. Human pangenomics stored in Amazon Simple Storage Service (Amazon S3);. Accessed: 13/11/2024. https://s3-us-west-2.amazonaws.com/human-pangenomics/index.html?prefix=working.
41. Ashkenazim trio data stored in the National Center for Biotechnology Information (NCBI);. Accessed: 13/11/2024. https://ftp-trace.ncbi.nlm.nih.gov/ReferenceSamples/giab/data/AshkenazimTrio.
42. The HG002 Hi-C sequencing data stored in Amazon Simple Storage Service (Amazon S3);. Accessed: 13/11/2024. https://s3-us-west-2.amazonaws.com/human-pangenomics/index.html?prefix=NHGRI_UCSC_panel/HG002/hpp_HG002_NA24385_son_v1/hic/downsampled.
43. OpenAI(2024), ChatGPT (GPT-4o, November 13 Version) [Large language model]. Polish the sentence;. Accessed: 13/11/2024. https://chat.openai.com/chat.

Supplement

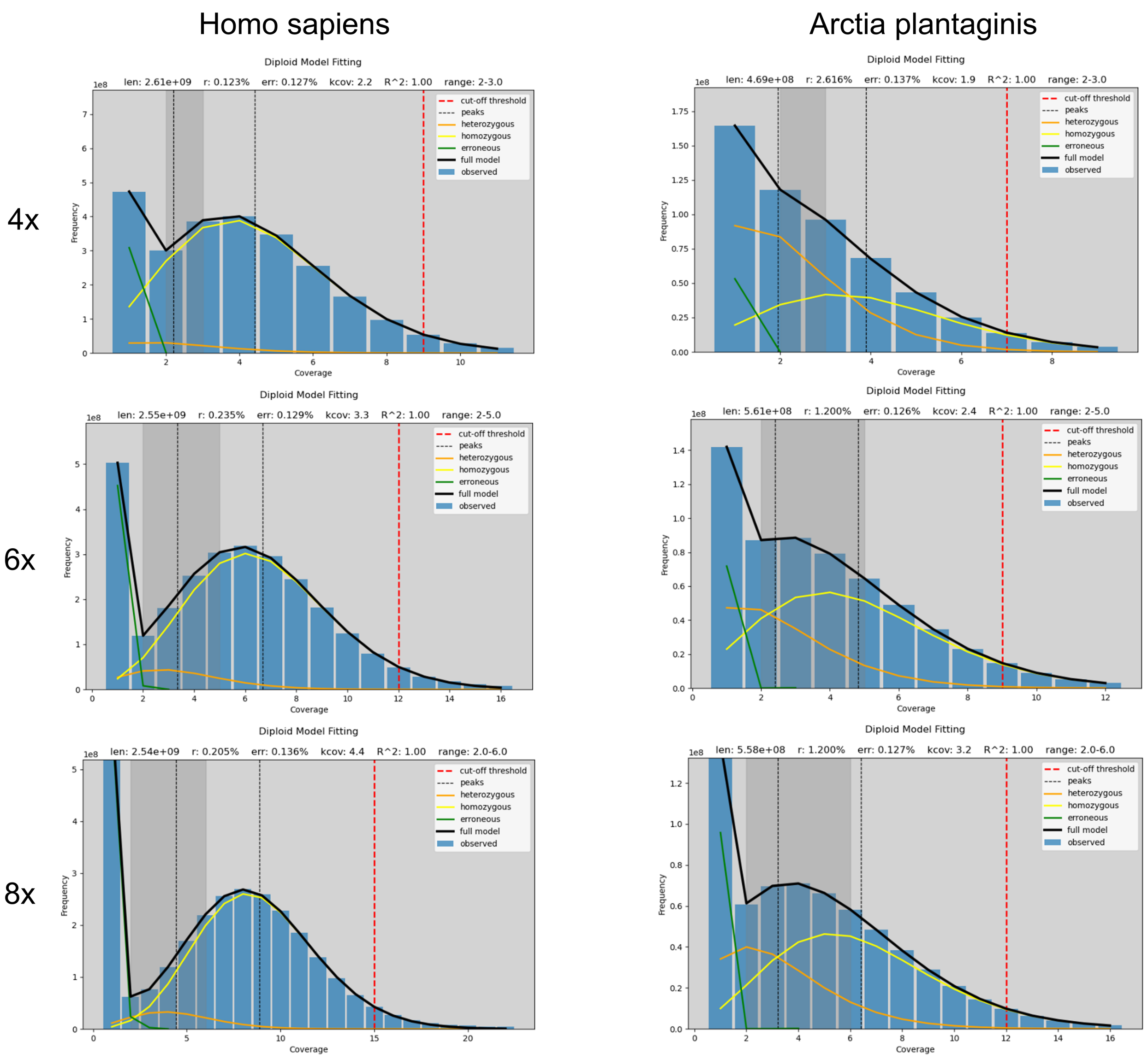

Figure S1. Schematic diagram of heterozygous region selection for *Homo sapiens* and *Arctia plantaginis* at 2×, 4×, and 6× coverage.

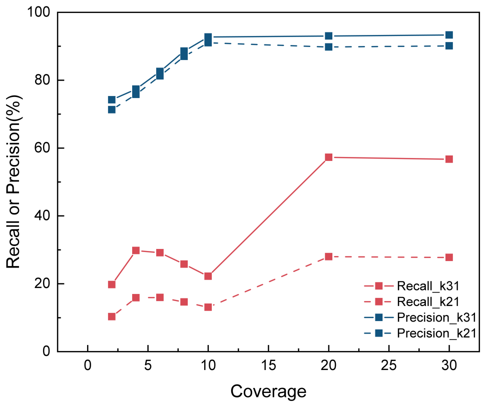

Figure S2. Heterozygous SNP calling results by DiscoSNP for HG002 PacBio HiFi sequencing data across varying coverage levels.

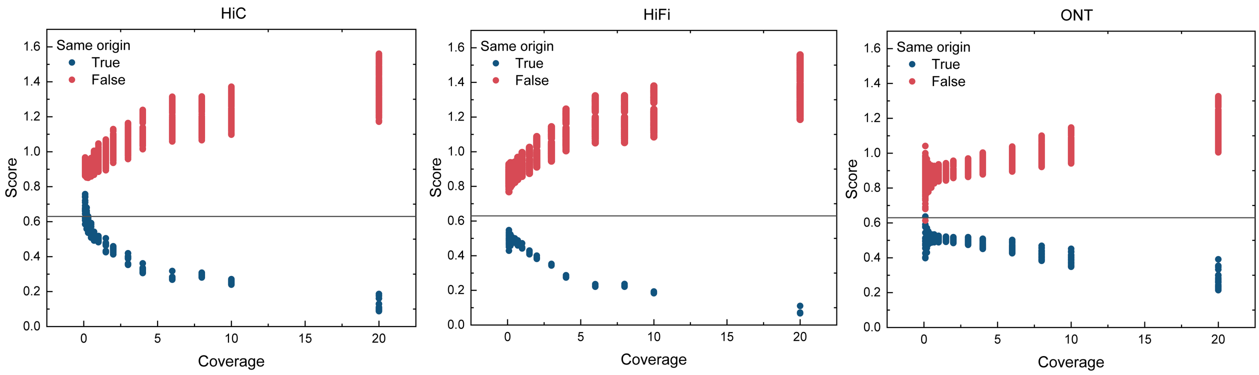

Figure S3. Detailed results of the sample swap scores for 20 human samples. Each sample includes sequencing data from three technologies: Hi-C, HiFi, and ONT. The horizontal line in the figure represents the threshold for determining whether the samples are identical, set at 0.63.

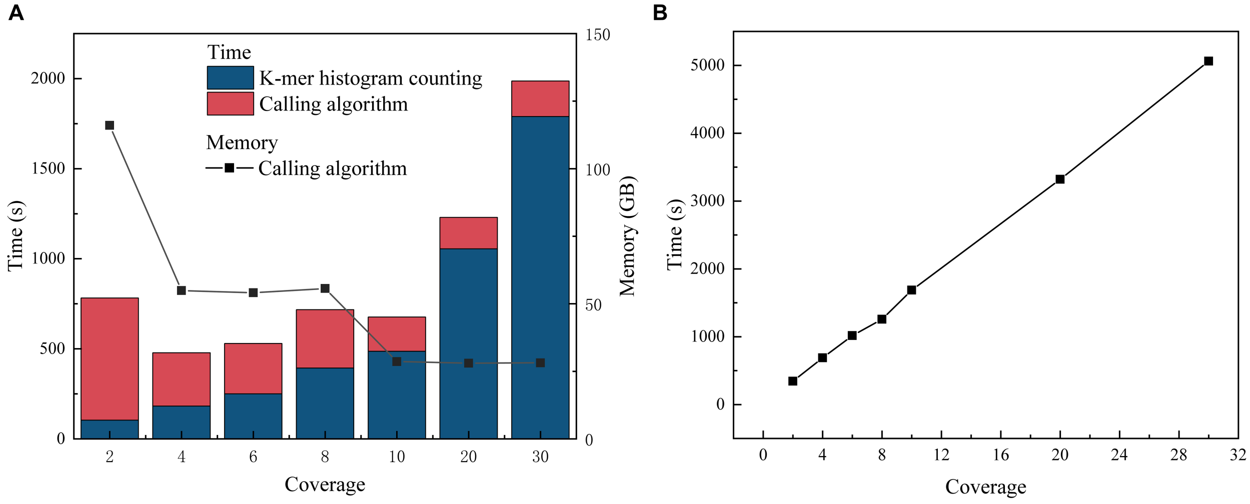

Figure S4. The time and memory consumption of the PISAD. Experiments are conducted on a platform with an Intel(R) Xeon(R) Gold 6348 CPU @ 2.60GHz. (A) The memory and time consumption for reference-free SNP calling with different sequencing depths in stage 1 (using 8 core). The memory consumption during the *k*-mer histogram counting phase depends on the maximum memory setting configured for DSK, which is set to 30 GB here. (B) The time consumption for *k*-mer counting on the sketch with different sequencing depths in stage 2 (using 1 core). The memory consumption is independent of the coverage and mainly depends on the size of the sketch, which is approximately 0.5GB in this case

Table S1. The performance of SNP calling under different *k*-mer sizes(HG002 PacBio HiFi data at 30x coverage)

k-mer	Precision(%)	Recall(%)	Memory(GB)	Time(s)
17	89.1	3.5	18.8	94
19	90.3	35.1	30.1	186
21	89.8	56.1	28.2	209
23	89.6	60.7	28.8	212
25	89.7	62.4	29.1	192
27	89.7	63.9	28.7	195
29	89.8	64.9	54.8	233
31	89.8	65.9	54.3	294

Table S2. The IDs of 20 human family trios form the HPRC and 1000 Genome Project

Child	Parents	
HG00438	HG00436	HG00437
HG00621	HG00619	HG00620
HG00673	HG00671	HG00672
HG00733	HG00731	HG00732
HG00735	HG01047	HG00734
HG01258	HG01256	HG01257
HG01361	HG01359	HG01360
HG01891	HG01890	HG01889
HG01928	HG01926	HG01927
HG01952	HG01950	HG01951
HG01978	HG01977	HG01976
HG02055	HG02053	HG02054
HG02080	HG02081	HG02082
HG02630	HG02628	HG02629
HG02717	HG02715	HG02716
HG02886	HG02884	HG02885
HG03492	HG03490	HG03491
HG03516	HG03515	HG03514
HG03540	HG03538	HG03539
HG03098	HG03096	HG03097

Manuscript

Click here to access/download
Supplementary Material
PISAD_Response_letter.docx
