# Supplementary material for: PISAD: reference-free intraspecies sample anomalies detection tool based on k-mer counting
Source: Gigascience. 2025 Jun 17;14:giaf061. doi: 10.1093/gigascience/giaf061 (PMC12202988; doi:10.1093/gigascience/giaf061)
Supplement: giaf061_Authors_Response_To_Reviewer_Comments_original_submission [file giaf061_authors_response_to_reviewer_comments_original_submission.pdf]

Author's Response To Reviewer Comments

Responses to the Reviewers' Comments

" PISAD: reference-free intraspecies sample anomalies detection tool based on k-mer counting "

By Zhantian Xu, Fan Nie, and Jianxin Wang

Dear Editors,

We would like to thank all the reviewers and AE for careful reading, constructive suggestions, and comments, which have significantly improved the presentation of our paper. We have revised the manuscript accordingly. The manuscript has also been double-checked, and the typos and grammar errors we found have been corrected. We hope that our revised manuscript is satisfactory.

In the following, we give our point-wise response. We copy the original review comments and mark them in italics and blue. Our responses are given in roman font. The changes in the manuscript are highlighted with red color.

All authors have read and approved the final version of the manuscript.

Thanks very much for your consideration!

Professor Jianxin Wang
School of Computer Science and Engineering,
Central South University
Changsha, Hunan, P.R. China
Email: jxwang@mail.csu.edu.cn

Answers to Reviewer #1

Comments:
"In PISAD, Xu et al. created a new software and algorithm for quickly detecting the similarity of two high-throughput sequencing samples for similar organisms at the sub-species level. This is similar to existing sketching approaches, but their innovation lies in using a reference-free SNP calling technique to avoid using a reference genome. This allows PISAD to work on various diploid organism sequencing projects that do not have good reference genomes -- an important task.
Overall, I found the paper relatively clear, although I was confused at some places. The methodology was well-motivated. The experimentation was thorough and well thought out. I have a few comments below that may improve the paper and its presentation.''
Response:
We greatly appreciate your careful consideration of our manuscript and their positive remarks. Thank you for your valuable comments, which motivate us to improve our work. Next, we will address your comments one by one.

Major Comments:
1."I feel strongly that software for a methodological paper should have a very simple install. This means either through (1) a package manager (conda), or (2) as a static binary (least preferable), or (3) have minimal dependencies for compiling. PISAD does not appear to be available in conda (the authors mention "recommend use conda to install", but I don't see PISAD on bioconda). Furthermore, it has non-trivial dependencies (hdf5, boost, DSK) that are not readily available on average linux machines. Please consider adding at least a self-contained bioconda install.
Some other notes:
- I think practitioners prefer dashed commands (--) versus positional commands. The commandline

interface is a bit cumbersome
- Based on the GitHub README, can variant calling be multithreaded?’’
Response:
We sincerely appreciate your feedback regarding the installation process. To simplify installation, PISAD is now available on Bioconda and can be installed directly using: Conda(conda install bioconda::pisad).
We have revised the command-line interface to adopt a standardized dashed command format for improved usability. The GitHub README has been comprehensively updated with clearer instructions and more detailed usage examples.
Variant calling supports multi-threading, and the README has been refined to explicitly clarify its usage, minimizing potential confusion.

2.“The authors confine themselves to diploid animal genomes. I think it would be very interesting if PISAD could work for 1) diploid plants and 2) allopolyploid genomes. Allopolyploids often "contain" two diploid genomes in a sense. Allopolyploid genomes may be difficult for this algorithm, but many plant geneticists would be interested in at least diploid plants. Effectiveness on diploid plants would allow this paper to reach a wider audience.. �spacing’
Response:
We sincerely appreciate your insightful suggestions. To broaden the applicability of PISAD, we have conducted additional experiments on the diploid plant Pyrus and extended the tool’s functionality to diploid plants. We have incorporated the Pyrus results into Figure 5C.
Regarding allopolyploid genomes, SNP calling in polyploids remains a complex challenge, even in reference-based approaches, making reference-free SNP calling even more difficult. As a result, this study primarily focuses on diploid species. However, we recognize the potential of extending PISAD to polyploid genomes and consider this an important direction for future research. We have expanded the discussion in Section Current Limitations to address these aspects more comprehensively.
“Additionally, our current study is exclusively focused on diploid species. Polyploid organisms have not been included in this investigation due to the inherent challenges in SNP calling associated with their complex genomic architectures, which warrant separate and more extensive future research.”

Minor Comments:
1.“I think Cortex (Iqbal et al., Nature Genetics 2012) should be cited in addition to DiscoSNP, since it's one of the first approaches. ‵’
Response:
We appreciate your suggestion and have incorporated a citation to Cortex in Section Introduction as follows:
"For example, Cortex and DiscoSnp++ construct a de Bruijn graph from raw data and detect specific patterns to call SNPs."

2.“The authors could make it more explicit that stage 1 does not work for Nanopore data ("high" error rate) but stage 2 works for Nanopore, perhaps at the end of the "Algorithm overview" section. ‵’
Response:
Thanks for your valuable suggestion. To enhance clarity, we have added the following statement at the end of Section Algorithm Overview:
"It is important to note that Stage 1 of our tool supports only low-error-rate sequencing data, meaning most ONT data (except duplex) cannot be processed. However, Stage 2 is compatible with various sequencing technologies. Therefore, at least one low-error-rate dataset is required for reliable sketch construction."

3.“The algorithm begins by reading the first 1000 points of the k-mer histogram…" what are the first 1000 points? Is the x-axis the k-mer frequency and y-axis is # of kmers? Is 1000 the k-mer count? Please clarify this sentence. ‵’
Response:
Thanks for your valuable suggestion. To prevent misunderstandings, we have revised the sentence in section SNP Calling as follows:

"reading the first 1000 entries from the histogram of k-mer abundances, which records the number of distinct k-mers for each occurrence frequency."
The x-axis and y-axis in Figure 2A represent k-mer occurrence frequency and the number of distinct k-mers, respectively.

4.“Then, FindGse[23] is employed to identify the frequency range of heterozygous k-mer…" the authors then state that they designed their own algorithm to detect heterozygous k-mers. I'm a bit confused about if FindGse is being used at all?”
Aside: perhaps the paper "Skmer: assembly-free and alignment-free sample identification using genome skims" may be of interest for low-coverage k-mer statistics.
Response:
Thanks for your valuable suggestion. We sincerely apologize for the confusion. FindGse was not used in our approach, as our evaluation showed that it is not suitable for low-coverage data. Instead, we developed a heuristic method to extract heterozygous regions from shallow sequencing data. To clarify this, we have revised the description in the SNP Calling section as follows:
"Existing genome analysis tools, such as FindGse, are typically designed for high-coverage data (>30×) and are not well-suited for shallow sequencing. Therefore, we developed a heuristic algorithm to estimate the range of heterozygous regions under low-coverage conditions."
We also appreciate your suggestion regarding Skmer. After reviewing this study, along with related works such as GenomeScope, we incorporated curve fitting using two negative binomial distributions to estimate genomic characteristics. Additionally, we visualized these results to assist users in making assessments (Figure 2A).

5.“The terms "SNPs supported by a length of…" and "isolated" SNPs need to be explained more. I believe these are terms used in the Kmer2SNP paper, but they are confusing without context. I'm not sure about the definition of "supported‵′
Response:
Thanks for your valuable suggestion. The phrase "SNPs supported by a length of" refers to the maximum possible expansion distance on both sides of an SNP. "Isolated SNP" refers to SNPs that are separated by at least (k−1)/2 bases, where k is the k-mer length.

To improve clarity, we have added a brief explanation of the complete SNP calling process and relevant terminology in Section SNP Calling. Additionally, Figure 2B has been included to illustrate key components. The revised description states:
"KmerSNP first identifies k-mers within heterozygous regions that differ by only a single nucleotide in the middle and designates them as potential SNPs. It then expands both sides of each potential SNP, where the maximum possible expansion distance is defined as the support length. Finally, the algorithm selects the final SNPs by computing the maximum-weight matching of all potential SNPs."

6.“We chose ONT data due to its high error rate, anticipating that it would present the greatest analytical challenge…" this paragraph appears to be slightly out of place since it's in the "SNP calling" section. But it talks about likelihood scores. ‵′
Response:
We acknowledge the misplaced placement of this paragraph within Section SNP Calling. To improve the logical flow, we have introduced a new section titled "Impact of SNP Site" and relocated the content accordingly.

7.“Can the authors mention the error rates of these ONT reads being used? ONT read error rates is highly variable and it can range from 95-99%, so a specific number is helpful ‵′
Response:
We appreciate your suggestion and have added the specific error rates associated with the ONT reads used in our study. The flow cell version and the accuracy of the ONT data are now specified, and more detailed experimental information has been included in Table 1.

8.“Fig. 4 is interesting but hard to read. Here are some issues:

- The blue and gray colours are not easy to differentiate for me. Using different markers (e.g. a square and circle) would help.
- The axis labels are hard to read, because they are flattened. E.g. "SNP call coverage" is not legible.
- If there's enough space, 2-d projections of Fig. 4 onto Coverage = 30 or SNP call coverage = 30 would help readers a lot and make the author's points more clear, since 3-d can be hard to visualize.‵′
Response:
Thank you for your feedback. We have made the following revisions to improve the clarity of Figure 4:
(1) The blue and gray colors have been replaced with more distinguishable colors (blue and red) to better differentiate the datasets.
(2) We have adjusted the axis labels to ensure they are more legible and added clear explanations of the x and y axes in the figure legend.
(3) A 2D projection of Figure 4A with the SNP call coverage axis at a value of 4 has been added as Figure 4C to assist readers in interpreting the data more easily.

9.‶As a comment, the 0.4 likelihood score seems arbitrary and unsatisfying. I'm not sure if the authors can do anything about this, however. I'm curious if the authors have thought about a theoretical basis for this value. ‵′
Response:
Thank you for your thoughtful comment. We recognize the concern regarding the 0.4 likelihood score, and we have made some modifications to address this.
As shown in Figure 2C, we revised the original sample swap score calculation to eliminate two empirical bias parameters and adjusted the input for the target individual's sketch to a sample with Ref/Alt values both set to 1. As shown in Figure5B, this change reduces the coverage requirement from 1.5x to 0.5x, which is lower than the 1x requirement of NTSM method. Details of this modification can be found in Section Calculation Score.
Additionally, to provide a more solid theoretical foundation, we have incorporated a simulation-based approach. By modeling scenarios with both identical and distinct samples, we derived a theoretical threshold for our method. This threshold of 0.63 was validated experimentally and is shown to perform well in real-world conditions, as demonstrated in Figures 3C and 4A, 4C.

10.‶Fig. 7: low likelihood scores indicate different origin. In Fig. 4: low likelihood scores indicate the same origin. Is this an error, or am I misunderstanding? ‵′
Response:
Thank you for pointing this out. You are correct that the labels in Figure 7 in the original manuscript were reversed. In fact, a low likelihood score represents the same origin. To resolve this inconsistency, we have corrected the labels and swapped the x and y axes in this figure to align with the other figures. The revised version is now shown in Figure 5C in the revised manuscript.

11.‶Our approach appears to mitigate the impact of high-error data by consolidating multiple counts at each site into a single value using the mode rather than the maximum, and by leveraging the ref/alt frequency distribution to classify genotypes." I didn't quite understand this. Maybe give an example to explain this further. ‵′
Response:
Thank you for your feedback. To make our methodology clearer, we have revised the description in Section Calculating Relatedness as following:
"Our method differs from ntsm in two key aspects. First, when merging multiple counts at each site, we use the mode instead of the maximum value. For example, if we observe counts like 8, 9, 8, 7 at a given site, we take the mode (which would be 8) rather than the maximum (which would be 9). Second, instead of using a fixed threshold to determine the genotype, we determine the threshold by identifying the minimum value of the Ref/(Ref+Alt) spectrum within a certain range. We believe these modifications help mitigate the impact of high error rates, leading to a more stable and compact genotype distribution."

Answers to Reviewer #2

Comments:
“This manuscript presents a new tool, PISAD, for detecting sample swaps in low-error-rate WGS sequencing data. This is an important problem and PISAD uses k-mer counting instead of read alignment for this task, making it potentially faster than other tools and more applicable to non-model species with no public genome assembly.
The associated code repository is well documented, and the code appears to be well written. I appreciated the authors providing a full report of how they used ChatGPT to refine their language. I tried to review it as much as I could and I found one case where chatgpt removed an important detail from the original writing, but the authors appeared to add the detail back into the submitted version of the manuscript. I take this as evidence that the chatgpt output was well scrutinized.
My major comments mostly refer to ensuring that claims in the abstract reflect the evidence in the paper and including more details in the methods. I think it is a little difficult to evaluate some of the claims in the paper without more detail in the methods. I hope my feedback is helpful. I didn't see line numbers, so I did my best to describe where my comments point to’’
Response:
Thank you for your thoughtful and constructive comments. We appreciate your recognition of our framework, PISAD, and its contributions to sample swaps detection.

Major Comments:
1.“One claim in the abstract is that PISAD works for low-error-rate data, but some other common types of low-error rate data were not tested in the manuscript (e.g. reduced-representation seq, GBS, pool-seq etc.). I think it would help to further clarify in the abstract the types of data PISAD was tested on, because lots of population-level datasets in non-model species (which seem to be the focus for PISAD) use RAD-seq or similar methods, which are technically low-error-rate but are generally low coverage. It is acknowledged in the current limitations section that extending to data types like RAD-seq is a future research direction, but most readers will only look at the abstract.’’
Response:
Thank you for your insightful comment. We apologize for the lack of clarity in the abstract. To address this, we have revised the abstract to specify that PISAD is currently applicable to WGS data only:
"We developed PISAD, a tool for validating sample identities in whole-genome sequencing (WGS) data without requiring reference information."
We also conducted preliminary tests on data types like RAD-seq and found that calling SNPs from such low-coverage data without reference posed greater challenges to precision. Specifically, the k-mer-based method resulted in a higher incidence of false SNPs, with a precision of only 70%–80%. As such, we acknowledge that extending PISAD to RAD-seq and similar low-coverage data types is a valuable direction for future research. This has also been clarified in Section Current Limitations.
"Meanwhile, calling SNPs from such data without reference poses greater challenges to precision compared to WGS data. Due to the absence of designated heterozygous regions, the k-mer-based method resulted in a higher incidence of false SNPs from homologous/repetitive sequences, yielding a precision of only 70%–80%. This will also constrain the tool’s resolution capability, warranting further investigation."

2.“The abstract also says that PISAD performs similarly to reference-based tools (plural), but as far as I can tell only one reference-based tool (ntsm) was investigated. It would be nice if the abstract said what tools were used for comparison.’’
Response:
Thank you for your comment. You are correct that we only compared PISAD with NTSM, as it is the only reference-based tool we are aware of that supports both fastq sequencing data and high-error-rate data such as ONT. To avoid any misunderstanding, we have revised it in Abstract as follows:
"Our evaluation showed that PISAD achieves a lower data coverage requirement (0.5×) compared to the reference-based tool NTSM and is broadly applicable to multiple diploid species."

3.“Under the "SNP calling" section in methods, 2nd paragraph, there is an algorithm to determine a range of coverage values that is likely predominated by k-mers from heterozygous regions and it is claimed that this algorithm helps identify heterozygous k-mer regions in lower coverage datasets. If I understand correctly, the algorithm is to define peaks as a sequence of 3 k-mer frequencies with an upward trend, followed by 3 frequencies with a downward trend and the first such peak is usually taken as the mean of

the heterozygous k-mer distribution. I am a little skeptical whether this works because it seems like a small fluctuation in k-mer frequencies due to sampling error could create a false peak. There is also a set of values for calculating the bounds of the heterozygous k-mer peak that seems somewhat arbitrary. Are these bounds configurable by a user? Will PISAD output any type of histogram with the coverage cutoffs marked so that users can confirm that the automaticheterozygous peak identification gives reasonable results? I think it would be helpful to have an example k-mer histogram from a low coverage dataset showing how this algorithm identifies peaks. I am also a little unsure whether it is necessary to only search for k-mers in the heterozygous peak, besides potentially a gain in computational speed. The distribution of heterozygous k-mer frequencies should follow a Poisson or negative binomial distribution from 0 to infinity, so there could be true heterozygous k-mers outside the peak.''

Response:

Thank you for your detailed comment. We appreciate your concerns regarding the algorithm for identifying heterozygous k-mer regions.

We first address your concern about the potential presence of false peaks. Our algorithm restricts peak identification to the region where the cumulative k-mer frequency sum remains below the 95% threshold. This design significantly reduces the probability of false positive peaks, as illustrated in Figure 2A. The rationale behind this approach is twofold: First, k-mers within this high-frequency region (constituting 95% of the cumulative spectrum) exhibit robust frequency signals that are inherently resistant to sampling variations. Second, while low-frequency regions (beyond the 95% threshold) may be prone to noise-induced fluctuations, these areas are explicitly excluded from our peak detection framework, thereby enhancing the overall reliability of our results.

Next, we sincerely apologize for any confusion caused regarding the selection of heterozygous regions and the reasoning behind it. We have now made comprehensive revisions in the SNP calling section, as detailed below.

"Theoretically, heterozygous region distribution adheres to a Poisson or negative binomial distribution ranging from 0 to infinity. However, directly selecting k-mers from these regions 1) greatly increases algorithm runtime and memory consumption and 2) generates numerous false SNPs from chimeric sequences (overlapping k-mers in the SNP sequence actually originating from different reads) and homologous/repetitive sequences. Inspired by the Kmer2SNP concept, we heuristically designate heterozygous peaks of 0.5×–1.5× as heterozygous regions (minimum left boundary of 2 to exclude erroneous k-mers), ensuring the capture of most k-mers in these regions while maximally reducing runtime, memory usage, and false SNPs."

Moreover, to further improve the reliability and usability of this algorithm module, we allow users to specify any parameters and provide histograms with clear annotations of heterozygous regions and other relevant information for user verification. Additionally, drawing inspiration from methods like Genomescope and skmer, we incorporated curve fitting using two negative binomial distributions to estimate genomic characteristics, facilitating further user validation (Figure 2A). For more detailed heterozygous region selection results across various coverage depths, please refer to Supplemental Figure 1.

"Finally, we fitted the k-mer frequency distribution using two negative binomial distributions and provided a rough estimate of genomic characteristics to assist users in evaluating the correctness of the algorithm's selection. The results of heterozygous region selection are shown in Figure 2A and Supplemental Figure 1."

4."I think describing the SNP calling process, once heterozygous k-mers are identified, in more detail would be helpful. It appears the manuscript presents an optimized version of a previous algorithm, kmer2snp, which sounds like a great advance by itself and perhaps warrants mentioning in the abstract. I read about kmer2snp and it seems the algorithm essentially identifies k-mer pairs that differ at their central bp as putative SNPs, but this is not really described in the current manuscript. Providing a brief description of the algorithm would be helpful.''

Response:

Thank you for your suggestion. We have added a brief explanation of the complete SNP calling process and relevant terminology in Section SNP Calling as following:

"KmerSNP first identifies k-mers within heterozygous regions that differ by only a single nucleotide in the middle, designating them as potential SNPs. It then expands both sides of each potential SNP, where the maximum possible expansion distance is defined as the support length. Finally, the algorithm refines SNP selection by computing the maximum-weight matching of all potential SNPs."

Additionally, Figure 2B has been included to illustrate key components.

5.“The methods section would benefit from having a section that describes the benchmarking datasets in more detail. Some details of the datasets are included in the "data availability" section and table 2, but I cannot tell just by reading the ID numbers what the underlying biology is of the samples. What tissue are we looking at? Are these cancer samples? How many samples are used for each test?’’
Response:
Thank you for your suggestion. We have revised the table to include more detailed biological characteristics of the samples, including tissue type, whether the samples are from cancer or non-cancer sources, and the number of samples used in each test. This updated table has now been relocated to Table 1 in the revised manuscript for better clarity.

6.“Table 1: What coverage level was used for the comparison in this table? This seems to be a main determinant of precision and recall.’’
Response:
Thank you for your comment. Table 1 in the original manuscript used data from HG002 PacBio HiFi with 30× coverage. This table focuses on illustrating the impact of k-mer length on the results. We have now added the coverage information to the table legend for clarity, and the table has been relocated to Supplementary Table 1.

7.“It is claimed that in Fig. 5A the new tool of the manuscript has lower variance in relatedness score than ntsm (under heading for "calculating relatedness" in results). This claim should be backed by some kind of statistical test. It's most likely significant just from a glance.’’
Response:
Thank you for your valuable suggestion. We have conducted a Levene’s test for variance in the data and updated Figure 5A to include the results after significance testing to confirm the observed differences.

Minor Comments:
1.“Does the name PISAD mean anything? I was expecting this name to be an acronym, but I didn't see one in the paper.”
Response:
Thank you for your observation. We have added an explanation of the meaning of PISAD in the Section Introduction as follows:
"In this work, we developed PISAD, a Phased Intraspecies Sample Anomalies Detection tool, suitable for multiple species without reference information."

2.“I am curious why the likelihood score cutoff of 0.4 appears to work so well. I don't think this question needs to be answered for the tool to be useful (significance score cutoffs are always somewhat arbitrary) but I am curious if the choice of cutoff has something to do with the coverage levels used and the error rates of the datasets?”
Response:
Thank you for your thoughtful comment. We recognize the concern regarding the 0.4 likelihood score, and we have made some modifications to address this.
As shown in Figure 2C, we revised the original sample swap score calculation to eliminate two empirical bias parameters and adjusted the input for the target individual's sketch to a sample with Ref/Alt values both set to 1. As shown in Figure 5B, this change reduces the coverage requirement from 1.5x to 0.5x, which is lower than the 1x requirement of NTSM method. Details of this modification can be found in Section Calculation Score.
Additionally, to provide a more solid theoretical foundation, we have incorporated a simulation-based approach. By modeling scenarios with both identical and distinct samples, we derived a theoretical threshold for our method. This threshold of 0.63 was validated experimentally and is shown to perform well in real-world conditions, as demonstrated in Figures 3C and 4A, 4C.
Regarding threshold selection, our analysis reveals two key observations from Figures 3C and 4C: (1) increasing sequencing coverage enhances the discriminative power between identical and different samples, while (2) higher error rates reduce this differentiation capability. Based on these findings, we established the identification threshold at 0.63, corresponding to the average similarity value between

same and different samples under the most challenging condition (0.1× coverage) in our simulations. This conservative approach ensures robust sample discrimination even in low-coverage scenarios. Furthermore, to comprehensively evaluate the impact of sequencing errors, we have incorporated additional analysis in Section Impact of Stage 2 Coverage, specifically examining how error rates influence identification accuracy.
"Meanwhile, we observsed that in sample swap detection, ONT data presented the greatest challenge in distinguishing samples. Its relatively high error rate reduces the number of effective sites, necessitating higher coverage for improved discrimination."

3."Low precision seems like it could be an issue, though perhaps it is not. PISAD has a recall/sensitivity of about 50 % even at high coverage (Fig. 3B). In other words, only 50 % of the SNPs that are truly present in the sample are being called. At first glance, this seems to be lower than similar reference free SNP calling (see discosnp papers). My expectation is that if enough SNPs can be called to distinguish samples this is not an issue. However, the manuscript mainly examines whole genome data, and I would expect this to be more of a problem for reduced representation datasets (RAD-seq, GBS, exome-seq, RNA-seq), where only a small portion of the genome is sequenced and thus only a small portion of SNPs are sampled."
Response:
Thank you for your comment. We appreciate the raised concerns about sufficient SNP sites and have introduced corresponding improvements to tackle this challenge.
First, we would like to clarify the performance of similar reference-free SNP calling tools in whole-genome sequencing, such as DiscoSnp. We conducted the evaluation under the same conditions as in Figure 3B, with the results presented in Supplementary Figure 2. The comparative analysis reveals that both DiscoSNP and Kmer2SNP demonstrate comparable performance in heterozygous SNP detection, with recall rates plateauing at 50-60% even at high sequencing coverage. Notably, this performance falls substantially below the 80% recall rate reported in the original DiscoSNP study. This discrepancy likely stems from fundamental differences in evaluation frameworks - while previous reports used simplified single-chromosome simulations, our whole-genome assessment more accurately reflects real-world complexity. We hypothesize that in whole-genome scenarios, the presence of cross-chromosomal k-mers interferes with the formation of characteristic symmetrical bubble structures, thereby impairing detection sensitivity for heterozygous variants.

Secondly, we acknowledge the potential concerns regarding the sufficiency of SNP sites. To address this issue, we have supplemented Section Impact of SNP Sites with a new experiment designed to systematically evaluate the tool's performance stability under varying SNP site quantities through simulation studies (Figure 4D):
"Meanwhile, we further investigated the number of SNPs used in the variant sketch in stage 1. From all heterozygous SNPs called at 30× coverage in HG002, we randomly sampled 5k, 10k, 20k, 40k, 60k, 100k, and 200k SNP sites, corresponding to 0.25%, 0.5%, 1%, 2%, 3%, 5%, and 10% of the total heterozygous SNPs in HG002. Subsequently, we tested the data from different sequencing technologies at 0.5× coverage. For each case, we performed ten repetitions and recorded the mean, minimum, and maximum values of the score. As shown in Figure 4D, the stability of the variant sketch for low-coverage data decreases as the number of SNPs decreases, particularly for ONT data. We attribute this to its high error rate, which significantly reduces the number of effective sites. Therefore, we conservatively estimate that at least approximately 20k sites are required to maintain basic stability, ensuring at least 50 effective sites even for ONT data at a coverage as low as 0.5×. When the number of effective sites falls below 50, we issue an unreliability warning, urging the reselection of more SNP sites for testing. Coincidentally, we observed that tools like NGSCheckMate and somalier also utilize approximately 20,000 SNP sites to assess sample relationships, further underscoring the significance of this site count."

Cookie Preference Center

We use cookies which are necessary to make our site work. We may also use additional cookies to analyze, improve and personalize our content and your digital experience. For more information, see our Cookie Policy.

You may choose not to allow some types of cookies. However, blocking some types may impact your experience of our site and the services we are able to offer. See the different category headings below to find out more or change your settings.

Allow all

Manage Consent Preferences

Strictly Necessary Cookies

Always active

These cookies are necessary for the website to function and cannot be switched off in our systems. They are usually only set in response to actions made by you which amount to a request for services, such as setting your privacy preferences, logging in or filling in forms. You can set your browser to block or alert you about these cookies, but some parts of the site will not then work. These cookies do not store any personally identifiable information.

Functional Cookies

These cookies enable the website to provide enhanced functionality and personalisation. They may be set by us or by third party providers whose services we have added to our pages. If you do not allow these cookies then some or all of these services may not function properly.

Performance Cookies

These cookies allow us to count visits and traffic sources so we can measure and improve the performance of our site. They help us to know which pages are the most and least popular and see how visitors move around the site.

Targeting Cookies

These cookies may be set through our site by our advertising partners. They may be used by those companies to build a profile of your interests and show you relevant adverts on other sites. If you do not allow these cookies, you will experience less targeted advertising.

Cookie List

Clear

Apply Cancel
Consent Leg.Interest

Confirm my choices

Powered by onetrus
