# Supplementary material for: PISAD: reference-free intraspecies sample anomalies detection tool based on k-mer counting
Source: Gigascience. 2025 Jun 17;14:giaf061. doi: 10.1093/gigascience/giaf061 (PMC12202988; doi:10.1093/gigascience/giaf061)
Supplement: giaf061_Authors_Response_To_Reviewer_Comments_revision_1 [file giaf061_authors_response_to_reviewer_comments_revision_1.pdf]

Author's Response To Reviewer Comments

Responses to the Reviewers' Comments

" PISAD: reference-free intraspecies sample anomalies detection tool based on k-mer counting "

By Zhantian Xu, Fan Nie, and Jianxin Wang

Dear Editors,

We would like to thank all the reviewers and AE for careful reading, constructive suggestions, and comments, which have significantly improved the presentation of our paper. We have revised the manuscript accordingly. The manuscript has also been double-checked, and the typos and grammar errors we found have been corrected. We hope that our revised manuscript is satisfactory.

In the following, we give our point-wise response. We copy the original review comments and mark them in italics and blue. Our responses are given in roman font. The changes in the manuscript are highlighted with red color.

All authors have read and approved the final version of the manuscript.

Thanks very much for your consideration!

Professor Jianxin Wang
School of Computer Science and Engineering,
Central South University
Changsha, Hunan, P.R. China
Email: jxwang@mail.csu.edu.cn

Answers to Reviewer #1

"I appreciate the thoughtful responses to my comments. The additional analyses presented strengthen the paper. I have one final minor comment.''
Response:
We greatly appreciate your careful consideration of our manuscript and their positive remarks. Thank you for your valuable comments, which motivate us to improve our work. Next, we will address your comments one by one.

Minor Comments:
1."For the statement: "Theoretically, heterozygous region distribution adheres to a Poisson or negative binomial distribution ranging from 0 to infinity." t would be good to provide a citation for this statement. Here is one suggestion: https://doi.org/10.1038/s41467-020-14998-3''
Response:
We sincerely appreciate your suggestion. We've added the Genomescope2 reference to this passage.
"Theoretically, heterozygous region distribution adheres to a Poisson or negative binomial distribution ranging from 0 to infinity[26]."
[26]Ranallo-Benavidez TR, Jaron KS, Schatz MC. GenomeScope2.0 and Smudgeplot for reference-free profiling of polyploid genomes. Nature communications 2020;11(1):1432

Cookie Preference Center

We use cookies which are necessary to make our site work. We may also use additional cookies to analyze, improve and personalize our content and your digital experience. For more information, see our Cookie Policy.

You may choose not to allow some types of cookies. However, blocking some types may impact your experience of our site and the services we are able to offer. See the different category headings below to find out more or change your settings.

Allow all

Manage Consent Preferences

Strictly Necessary Cookies

Always active

These cookies are necessary for the website to function and cannot be switched off in our systems. They are usually only set in response to actions made by you which amount to a request for services, such as setting your privacy preferences, logging in or filling in forms. You can set your browser to block or alert you about these cookies, but some parts of the site will not then work. These cookies do not store any personally identifiable information.

Functional Cookies

These cookies enable the website to provide enhanced functionality and personalisation. They may be set by us or by third party providers whose services we have added to our pages. If you do not allow these cookies then some or all of these services may not function properly.

Performance Cookies

These cookies allow us to count visits and traffic sources so we can measure and improve the performance of our site. They help us to know which pages are the most and least popular and see how visitors move around the site.

Targeting Cookies

These cookies may be set through our site by our advertising partners. They may be used by those companies to build a profile of your interests and show you relevant adverts on other sites. If you do not allow these cookies, you will experience less targeted advertising.

Cookie List

Clear

Apply Cancel
Consent Leg.Interest

Confirm my choices

Powered by onetrust
