# Supplementary material for: PISAD: reference-free intraspecies sample anomalies detection tool based on k-mer counting
Source: Gigascience. 2025 Jun 17;14:giaf061. doi: 10.1093/gigascience/giaf061 (PMC12202988; doi:10.1093/gigascience/giaf061)
Supplement: giaf061_Reviewer_1_Report_Original_submission — Jim Shaw -- 12/11/2024 [file giaf061_reviewer_1_report_original_submission.pdf]

Reviewer Report

Title: PISAD: reference-free intraspecies sample anomalies detection tool based on k-mer counting

Version: Original Submission **Date:** 12/11/2024

Reviewer name: Jim Shaw

Reviewer Comments to Author:

In PISAD, Xu et al. created a new software and algorithm for quickly detecting the similarity of two high-throughput sequencing samples for similar organisms at the sub-species level. This is similar to existing sketching approaches, but their innovation lies in using a reference-free SNP calling technique to avoid using a reference genome. This allows PISAD to work on various diploid organism sequencing projects that do not have good reference genomes -- an important task.

Overall, I found the paper relatively clear, although I was confused at some places. The methodology was well-motivated. The experimentation was thorough and well thought out. I have a few comments below that may improve the paper and its presentation.

Major:

1. I feel strongly that software for a methodological paper should have a very simple install. This means either through (1) a package manager (conda), or (2) as a static binary (least preferable), or (3) have minimal dependencies for compiling. PISAD does not appear to be available in conda (the authors mention "recommend use conda to install", but I don't see PISAD on bioconda). Furthermore, it has non-trivial dependencies (hdf5, boost, DSK) that are not readily available on average linux machines. Please consider adding at least a self-contained bioconda install.

Some other notes:

- I think practitioners prefer dashed commands (--) versus positional commands. The commandline interface is a bit cumbersome
- Based on the GitHub README, can variant calling be multithreaded?

2. The authors confine themselves to diploid animal genomes. I think it would be very interesting if PISAD could work for 1) diploid plants and 2) allopolyploid genomes. Allopolyploids often "contain" two diploid genomes in a sense. Allopolyploid genomes may be difficult for this algorithm, but many plant geneticists would be interested in at least diploid plants. Effectiveness on diploid plants would allow this paper to reach a wider audience.

Minor:

I think Cortex (Iqbal et al., Nature Genetics 2012) should be cited in addition to DiscoSNP, since it's one of the first approaches.

The authors could make it more explicit that stage 1 does not work for Nanopore data ("high" error rate) but stage 2 works for Nanopore, perhaps at the end of the "Algorithm overview" section.

"The algorithm begins by reading the first 1000 points of the k-mer histogram…" what are the first 1000 points? Is the x-axis the k-mer frequency and y-axis is # of kmers? Is 1000 the k-mer count? Please clarify this sentence.

"Then, FindGse[23] is employed to identify the frequency range of heterozygous k-mer…" the authors

then state that they designed their own algorithm to detect heterozygous k-mers. I'm a bit confused about if FindGse is being used at all?
Aside: perhaps the paper "Skmer: assembly-free and alignment-free sample identification using genome skims" may be of interest for low-coverage k-mer statistics.
The terms "SNPs supported by a length of…" and "isolated" SNPs need to be explained more. I believe these are terms used in the Kmer2SNP paper, but they are confusing without context. I'm not sure about the definition of "supported".
"We chose ONT data due to its high error rate, anticipating that it would present the greatest analytical challenge…" this paragraph appears to be slightly out of place since it's in the "SNP calling" section. But it talks about likelihood scores.
Can the authors mention the error rates of these ONT reads being used? ONT read error rates is highly variable and it can range from 95-99%, so a specific number is helpful.
Fig. 4 is interesting but hard to read. Here are some issues:
- The blue and gray colours are not easy to differentiate for me. Using different markers (e.g. a square and circle) would help.
- The axis labels are hard to read, because they are flattened. E.g. "SNP call coverage" is not legible.
- If there's enough space, 2-d projections of Fig. 4 onto Coverage = 30 or SNP call coverage = 30 would help readers a lot and make the author's points more clear, since 3-d can be hard to visualize.
As a comment, the 0.4 likelihood score seems arbitrary and unsatisfying. I'm not sure if the authors can do anything about this, however. I'm curious if the authors have thought about a theoretical basis for this value.
Fig. 7: low likelihood scores indicate different origin. In Fig. 4: low likelihood scores indicate the same origin. Is this an error, or am I misunderstanding?
"Our approach appears to mitigate the impact of high-error data by consolidating multiple counts at each site into a single value using the mode rather than the maximum, and by leveraging the ref/alt frequency distribution to classify genotypes." I didn't quite understand this. Maybe give an example to explain this further.

Methods

Are the methods appropriate to the aims of the study, are they well described, and are necessary controls included? Choose an item.

Conclusions

Are the conclusions adequately supported by the data shown? Choose an item.

Reporting Standards

Does the manuscript adhere to the journal's guidelines on minimum standards of reporting? Choose an item.

Choose an item.

Statistics

Are you able to assess all statistics in the manuscript, including the appropriateness of statistical tests used? Choose an item.

Quality of Written English

Please indicate the quality of language in the manuscript: Choose an item.

Declaration of Competing Interests

Please complete a declaration of competing interests, considering the following questions:

- Have you in the past five years received reimbursements, fees, funding, or salary from an organisation that may in any way gain or lose financially from the publication of this manuscript, either now or in the future?
- Do you hold any stocks or shares in an organisation that may in any way gain or lose financially from the publication of this manuscript, either now or in the future?
- Do you hold or are you currently applying for any patents relating to the content of the manuscript?
- Have you received reimbursements, fees, funding, or salary from an organization that holds or has applied for patents relating to the content of the manuscript?
- Do you have any other financial competing interests?
- Do you have any non-financial competing interests in relation to this paper?

If you can answer no to all of the above, write 'I declare that I have no competing interests' below. If your reply is yes to any, please give details below.

I declare that I have no competing interests.

I agree to the open peer review policy of the journal. I understand that my name will be included on my report to the authors and, if the manuscript is accepted for publication, my named report including any attachments I upload will be posted on the website along with the authors' responses. I agree for my report to be made available under an Open Access Creative Commons CC-BY license (http://creativecommons.org/licenses/by/4.0/). I understand that any comments which I do not wish to be included in my named report can be included as confidential comments to the editors, which will not be published.

Choose an item.

To further support our reviewers, we have joined with Publons, where you can gain additional credit to further highlight your hard work (see: https://publons.com/journal/530/gigascience). On publication of this paper, your review will be automatically added to Publons, you can then choose whether or not to claim your Publons credit. I understand this statement.

Yes Choose an item.
