# Supplementary material for: PISAD: reference-free intraspecies sample anomalies detection tool based on k-mer counting
Source: Gigascience. 2025 Jun 17;14:giaf061. doi: 10.1093/gigascience/giaf061 (PMC12202988; doi:10.1093/gigascience/giaf061)
Supplement: giaf061_Reviewer_2_Report_Original_submission — Miles Roberts -- 1/23/2025 [file giaf061_reviewer_2_report_original_submission.pdf]

Reviewer Report

Title: PISAD: reference-free intraspecies sample anomalies detection tool based on k-mer counting

Version: Original Submission **Date:** 1/23/2025

Reviewer name: Miles Roberts

Reviewer Comments to Author:

This manuscript presents a new tool, PISAD, for detecting sample swaps in low-error-rate WGS sequencing data. This is an important problem and PISAD uses k-mer counting instead of read alignment for this task, making it potentially faster than other tools and more applicable to non-model species with no public genome assembly.
The associated code repository is well documented, and the code appears to be well written. I appreciated the authors providing a full report of how they used ChatGPT to refine their language. I tried to review it as much as I could and I found one case where chatgpt removed an important detail from the original writing, but the authors appeared to add the detail back into the submitted version of the manuscript. I take this as evidence that the chatgpt output was well scrutinized.
My major comments mostly refer to ensuring that claims in the abstract reflect the evidence in the paper and including more details in the methods. I think it is a little difficult to evaluate some of the claims in the paper without more detail in the methods. I hope my feedback is helpful. I didn't see line numbers, so I did my best to describe where my comments point to.
Major comments:
1. One claim in the abstract is that PISAD works for low-error-rate data, but some other common types of low-error rate data were not tested in the manuscript (e.g. reduced-representation seq, GBS, pool-seq etc.). I think it would help to further clarify in the abstract the types of data PISAD was tested on, because lots of population-level datasets in non-model species (which seem to be the focus for PISAD) use RAD-seq or similar methods, which are technically low-error-rate but are generally low coverage. It is acknowledged in the current limitations section that extending to data types like RAD-seq is a future research direction, but most readers will only look at the abstract.
2. The abstract also says that PISAD performs similarly to reference-based tools (plural), but as far as I can tell only one reference-based tool (ntsm) was investigated. It would be nice if the abstract said what tools were used for comparison.
3. Under the "SNP calling" section in methods, 2nd paragraph, there is an algorithm to determine a range of coverage values that is likely predominated by k-mers from heterozygous regions and it is claimed that this algorithm helps identify heterozygous k-mer regions in lower coverage datasets. If I understand correctly, the algorithm is to define peaks as a sequence of 3 k-mer frequencies with an upward trend, followed by 3 frequencies with a downward trend and the first such peak is usually taken as the mean of the heterozygous k-mer distribution. I am a little skeptical whether this works because it seems like a small fluctuation in k-mer frequencies due to sampling error could create a false peak. There is also a set of values for calculating the bounds of the heterozygous k-mer peak that seems somewhat arbitrary. Are these bounds configurable by a user? Will PISAD output any type of histogram

with the coverage cutoffs marked so that users can confirm that the automatic heterozygous peak identification gives reasonable results? I think it would be helpful to have an example k-mer histogram from a low coverage dataset showing how this algorithm identifies peaks. I am also a little unsure whether it is necessary to only search for k-mers in the heterozygous peak, besides potentially a gain in computational speed. The distribution of heterozygous k-mer frequencies should follow a Poisson or negative binomial distribution from 0 to infinity, so there could be true heterozygous k-mers outside the peak.

4. I think describing the SNP calling process, once heterozygous k-mers are identified, in more detail would be helpful. It appears the manuscript presents an optimized version of a previous algorithm, kmer2snp, which sounds like a great advance by itself and perhaps warrants mentioning in the abstract. I read about kmer2snp and it seems the algorithm essentially identifies k-mer pairs that differ at their central bp as putative SNPs, but this is not really described in the current manuscript. Providing a brief description of the algorithm would be helpful.

5. The methods section would benefit from having a section that describes the benchmarking datasets in more detail. Some details of the datasets are included in the "data availability" section and table 2, but I cannot tell just by reading the ID numbers what the underlying biology is of the samples. What tissue are we looking at? Are these cancer samples? How many samples are used for each test?

6. Table 1: What coverage level was used for the comparison in this table? This seems to be a main determinant of precision and recall.

7. It is claimed that in Fig. 5A the new tool of the manuscript has lower variance in relatedness score than ntsm (under heading for "calculating relatedness" in results). This claim should be backed by some kind of statistical test. It's most likely significant just from a glance.

Minor comments:

1. Does the name PISAD mean anything? I was expecting this name to be an acronym, but I didn't see one in the paper.

2. I am curious why the likelihood score cutoff of 0.4 appears to work so well. I don't think this question needs to be answered for the tool to be useful (significance score cutoffs are always somewhat arbitrary) but I am curious if the choice of cutoff has something to do with the coverage levels used and the error rates of the datasets?

3. Low precision seems like it could be an issue, though perhaps it is not. PISAD has a recall/sensitivity of about 50 % even at high coverage (Fig. 3B). In other words, only 50 % of the SNPs that are truly present in the sample are being called. At first glance, this seems to be lower than similar reference free SNP calling (see discosnp papers). My expectation is that if enough SNPs can be called to distinguish samples this is not an issue. However, the manuscript mainly examines whole genome data, and I would expect this to be more of a problem for reduced representation datasets (RAD-seq, GBS, exome-seq, RNA-seq), where only a small portion of the genome is sequenced and thus only a small portion of SNPs are sampled.

Methods

Are the methods appropriate to the aims of the study, are they well described, and are necessary controls included? Choose an item.

Conclusions

Are the conclusions adequately supported by the data shown? Choose an item.

Reporting Standards

Does the manuscript adhere to the journal's guidelines on minimum standards of reporting? Choose an item.

Choose an item.

Statistics

Are you able to assess all statistics in the manuscript, including the appropriateness of statistical tests used? Choose an item.

Quality of Written English

Please indicate the quality of language in the manuscript: Choose an item.

Declaration of Competing Interests

Please complete a declaration of competing interests, considering the following questions:

- Have you in the past five years received reimbursements, fees, funding, or salary from an organisation that may in any way gain or lose financially from the publication of this manuscript, either now or in the future?
- Do you hold any stocks or shares in an organisation that may in any way gain or lose financially from the publication of this manuscript, either now or in the future?
- Do you hold or are you currently applying for any patents relating to the content of the manuscript?
- Have you received reimbursements, fees, funding, or salary from an organization that holds or has applied for patents relating to the content of the manuscript?
- Do you have any other financial competing interests?
- Do you have any non-financial competing interests in relation to this paper?

If you can answer no to all of the above, write 'I declare that I have no competing interests' below. If your reply is yes to any, please give details below.

I declare that I have no competing interests.

I agree to the open peer review policy of the journal. I understand that my name will be included on my report to the authors and, if the manuscript is accepted for publication, my named report including any attachments I upload will be posted on the website along with the authors' responses. I agree for my

report to be made available under an Open Access Creative Commons CC-BY license (http://creativecommons.org/licenses/by/4.0/). I understand that any comments which I do not wish to be included in my named report can be included as confidential comments to the editors, which will not be published.

Choose an item.

To further support our reviewers, we have joined with Publons, where you can gain additional credit to further highlight your hard work (see: https://publons.com/journal/530/gigascience). On publication of this paper, your review will be automatically added to Publons, you can then choose whether or not to claim your Publons credit. I understand this statement.

Yes Choose an item.
